# Features of Complementary Interactions of BmiRNAs with mRNAs of Human Genes

**DOI:** 10.3390/ijms27198773

**Published:** 2026-09-30

**Authors:** Anatoliy Ivashchenko, Anna Pyrkova, Saltanat Orazova, Raigul Niyazova

**Affiliations:** 1 Department of Biotechnology, Farabi University, Almaty 050040, Kazakhstan; saltanat.orazova@kaznu.kz; 2 Center for Bioinformatics and Nanomedicine, Almaty 050040, Kazakhstan; 3 Department of Computer Sciences, Farabi University, Almaty 050040, Kazakhstan; anna.pyrkova@kaznu.kz

**Keywords:** miRNA, mRNA, human genes, miRNA-mRNA interaction

## Abstract

More than three decades have passed since the discovery of miRNAs in human cells, yet many of their biological properties remain poorly understood. The aim of this study was to investigate whether miRNAs can interact with target gene mRNAs through fully complementary base pairing. Using the MirTarget program, we demonstrated that all nucleotides of miRNAs can form fully complementary interactions with nucleotides of target gene mRNAs. A total of 1036 miRNAs, which were identified by Backes et al. and we designated as BmiRNAs and mRNAs from 17,508 human genes, were analyzed, revealing several important characteristics. The vast majority of BmiRNAs were found to bind fully complementarily to the 3′-untranslated regions (3′UTRs) of target gene mRNAs. Many mRNAs contained binding sites (BSs) for multiple BmiRNAs, forming binding site clusters in which BmiRNAs may compete for interaction with the same mRNA. We also identified identical groups of up to four BmiRNAs capable of binding to the mRNAs of different genes. In addition, genes whose mRNAs contained up to eight BmiRNA binding sites were identified. These findings broaden our understanding of the mechanisms by which BmiRNAs regulate human gene expression at the mRNA translation stage and provide new insights into their potential role in post-transcriptional gene regulation.

## 1. Introduction

miRNAs were discovered more than three decades ago [1]. D.P. Bartel and other scientists pioneered the active study of miRNA properties [2,3]. The group led by C. Backes was also highly active in this field, identifying numerous new miRNAs and demonstrating their extensive diversity in the regulation of various metabolic processes [4,5,6]. Several databases containing from a thousand to several thousand miRNAs were identified. In this study, we refer to the miRNAs in the database created by Backes et al. as BmiRNAs [6]. Much attention has been paid to the interaction of miRNAs with mRNAs [7,8,9]. A significant part of research is devoted to the participation of miRNAs in cancer diseases [5,10,11]. The term microRNA has become firmly established in the scientific literature, although the molecules themselves are nanometer-sized, typically measuring approximately 6–9 nm in length. Based on this characteristic, it has been proposed that the abbreviation “miRNA” be interpreted as mRNA-inhibiting RNA. Another widely accepted concept is that miRNA–mRNA recognition is primarily determined by an 8–9-nucleotide sequence at the 5′ end of the miRNA, commonly referred to as the seed region [12,13,14]. Early prediction algorithms based on this concept identified numerous potential target genes. However, following the sequencing of the human genome, the number of predicted miRNA targets increased dramatically, raising concerns about the specificity of seed-based target prediction. In contrast, techniques such as the polymerase chain reaction (PCR) typically rely on primers of at least 20 nucleotides to achieve highly specific sequence recognition. Consequently, despite extensive research on miRNA–mRNA interactions over the past three decades, establishing unequivocal and highly specific miRNA–target relationships has remained challenging. This limitation has also affected the development of reliable miRNA-based biomarkers for disease prediction and the translation of miRNA research into effective therapeutic applications for oncological and cardiovascular diseases [14,15,16,17]. In light of these challenges, we investigated fully complementary interactions between BmiRNAs and the mRNAs of human genes. The translation process depends on the presence and concentration of miRNAs. The interaction energy of miRNA with mRNA depends on the miRNA length and the content of G-C pairs, the interaction energy of which is determined by three hydrogen bonds and A-U pairs by two. If the interaction of miRNA with mRNA is assessed only by seed, then selectivity will be virtually absent, and this is necessary. Identical miRNAs are known in objects that have diverged for millions of years [18,19]. Multiple target genes for miRNAs with completely complementary interactions with mRNAs pose difficulties in diagnosing diseases and creating drugs with such miRNAs. Therefore, the aim of this study is to determine which known miRNAs can interact with the mRNAs of target genes. The frequently arising requirement to confirm the interaction of miRNA with mRNA in wet assays is an overstatement, since the interaction of nucleotides in double-stranded DNA or RNA has long been proven. The aim of this study is to identify which BmiRNAs can interact with the mRNAs of which genes. Recently, such studies were performed with 3037 LmiRNAs from the London database [20].

## 2. Results

Our findings provide a framework for identifying the specific target genes regulated by individual BmiRNAs. Future studies will focus on a comprehensive analysis of BmiRNA–mRNA interactions to elucidate the regulatory relationships between target gene expression and their corresponding BmiRNAs. In the present study, we investigated which BmiRNAs influence mRNA translation. We demonstrated that many BmiRNA binding sites (BSs) are organized into clusters within target gene mRNAs, where binding sites for different BmiRNAs overlap by sharing common nucleotides. Such clustered organization creates competition among BmiRNAs for binding to the same mRNA molecule. Under these conditions, BmiRNAs with stronger binding affinity (i.e., more favorable free binding energy) are expected to exert a greater influence on translational regulation. However, the relative concentrations of competing BmiRNAs must also be considered.

### 2.1. Four BmiRNAs Binding with mRNA

In this study, we evaluated the potential competition among BmiRNAs for binding to target gene mRNAs. Figure 1 illustrates the interactions of BmiR-1441-3p, BmiR-2164-3p, BmiR-1189-3p, and BmiR-2289-3p with the mRNA of the *AASS* gene. The predicted binding sites are located within the BS cluster, beginning at nucleotide 3261, where the binding sites of different BmiRNAs overlap, resulting in potential competition for the same target region. Among the four BmiRNAs, BmiR-1441-3p exhibits the strongest predicted interaction, with a binding free energy (ΔG) of −123 kJ/mol, whereas the other three BmiRNAs have binding free energies of −113 kJ/mol. Because a more negative ΔG indicates a more stable miRNA–mRNA complex, BmiR-1441-3p is expected to have a higher probability of occupying the shared binding site and, consequently, a greater potential to influence the translation of the *AASS* mRNA. However, the relative intracellular concentrations of the competing BmiRNAs are also expected to contribute substantially to the final binding outcome.

Similar interaction patterns were identified in the 3′UTRs of mRNAs from numerous other human genes. The same four BmiRNAs—BmiR-1441-3p, BmiR-2164-3p, BmiR-1189-3p, and BmiR-2289-3p—were predicted to compete for binding to the mRNAs of 33 target genes (Tables S1–S5). These tables summarize the quantitative characteristics of the predicted interactions with the mRNAs of *ARGFX*, *ABCA13*, *AFMID*, *AIFM2*, *ALG1*, *AP3M1*, *APAF1*, *APOBEC3D*, *ATM*, *ATP1B4*, *BEND2*, *C11orf83*, *C22orf25*, *C3orf70*, *C9orf91*, *CARHSP1*, *CCL16*, *CENPI*, *CHP1*, *CLCC1*, *COX18*, *CRKL*, *CXADR*, *EIF2S3*, *ELK4*, *ELP3*, *ENPP1*, *EOGT*, *EXPH5*, *FAIM*, *FKBP5*, and *FXN*. Genes marked with ** and * contain BmiRNA binding sites within the 5′UTR and CDS, respectively.

The presence of identical nucleotide sequences capable of binding the same four BmiRNAs in the mRNAs of such a large number of genes suggests that these interactions have been evolutionarily conserved and may represent an important mechanism for the coordinated regulation of gene expression. Because only one BmiRNA can occupy a given binding site at a time, these four BmiRNAs are expected to compete for binding within the BS cluster, thereby influencing the efficiency of target gene translation.

The target genes identified in this study perform diverse biological functions and are associated with a wide range of physiological processes and diseases. For example, *ARGFX*, *ABCA13*, *AFMID*, *AIFM2*, *ALG1*, *AP3M1*, *APAF1*, *APOBEC3D*, *ATM*, and *ATP1B4* have distinct functional roles according to current gene annotation databases. This functional diversity makes it challenging to determine the individual contribution of each BmiRNA–mRNA interaction and to evaluate the potential use of specific BmiRNAs for the selective regulation of target gene expression.

To our knowledge, no previous studies have reported interactions between BmiR-1441-3p, miR-2164-3p, BmiR-1189-3p, or BmiR-2289-3p and the mRNAs of the target genes identified here. Consequently, the experimental validation of these predicted interactions remains an important objective for future research.

An additional challenge is the tissue-specific expression of both target genes and BmiRNAs. The expression levels of the target genes vary among different cell types, tissues, and organs, as do the expression levels of the four BmiRNAs. Moreover, extracellular BmiRNAs may be transported through the bloodstream and reach different tissues at varying concentrations. These factors substantially complicate the experimental investigation of BmiRNA–mRNA interactions under physiological conditions. Complexity is further increased because each BmiRNA may participate in multiple regulatory networks and interact with numerous target genes, making it difficult to distinguish the contribution of an individual BmiRNA from its broader regulatory functions.

Most of the predicted BmiRNA binding sites identified in this study are located within the 3′UTRs of target gene mRNAs. In contrast, the *AP3M1* and *CENPI* mRNAs contain BmiRNA binding sites within their 5′UTRs. Figure 2 illustrates the predicted interactions between the four BmiRNAs and the 5′UTR of the *AP3M1* mRNA.

A similar binding pattern was observed for BmiR-1441-3p, BmiR-2164-3p, BmiR-1189-3p, and BmiR-2289-3p in the 5′UTR of the *CENPI* mRNA (Figure 3).

Only one target gene, *BEND2*, was found to contain binding sites for all four BmiRNAs—BmiR-1441-3p, BmiR-2164-3p, BmiR-1189-3p, and BmiR-2289-3p—within the CDS of its mRNA (Figure 4). A distinctive feature of this BmiRNA group is that all four BmiRNAs are derived from the 3′ arm of their precursor hairpin structures (BmiRNA-3p). This common origin may reflect a functional characteristic of this group and could influence their loading into Argonaut proteins.

### 2.2. Three BmiRNAs Binding with mRNA

Groups consisting of three BmiRNAs were identified considerably more frequently than groups of four BmiRNAs. For example, the BmiRNA group comprising BmiR-531-5p, BmiR-1641-5p, and BmiR-1608-5p was predicted to target the mRNAs of 131 human genes, including *A2ML1*, *ACTR5*, *ADAL***,** *AKIRIN1*, *ANKRD16*, *ARFIP2*, *ASRGL1*, *ATCAY*, *ATP3F1*, *ATP5F1*, *BVES*, *C14orf132*, *C17orf85*, *C19orf40*, *C1QTNF2*, *C21orf62*, *C3orf62*, *CA5B*, *CALN1*, *CBFA2T2*, *CCDC90A*, *CCDC170*, *CCNB11P1*, *CENPL*, *CEB68*, *CERK*, *CFLAR*, *CISD2*, *CRTAM*, *CSNK1G1*, *CYP20A1*, *DAP3*, *DCAF16*, *DENR*, *DFFA* (*two BSs*), *DGKB*, *DPYD*, *DNAJC22*, *DSTYK*, *DDX51*, *ENPP6*, *ESYT2*, *F2RL3*, *FAM73A*, *FAM84B*, *FANCC*, *FBXL20*, *FBXO27*, *FGB*, *FOXK1*, *GALNT6*, *GK5*, *GMEB1*, *GRIPAP1*, *GTSE1*, *HRH1*, *IBA57*, *IL2RA*, *IL23R*, *ISPD*, *IYD*, *KCNA7*, *KCTD20*, *KIAA1143*, *KIAA1609*, *KRI1*, *LDLR*, *LIMS1*, *LIN52*, *LRAT*, *LRPAP1*, *MAFF*, *MCAM*, *MAP3K15*, *MCM8*, *MCUR1*, *METTL21A*, *METTL2A*, *MPPE1*, *MR1*, *MRI1*, *MTAP*, *MYO1C*, *MTDH*, *NAA16*, *NCMAP*, *NDUFAF7*, *NMNAT1*, *NOL9*, *NUPL1*, *OGFOD1*, *OLA1*, *PBLD*, *PCTP*, *PDDC1*, *PLA2G2D*, *PNPT1*, *PTCD3*, *PTPN14*, *RAB11FIP1*, *RBM41*, *RDM4B*, *SEC14L1*, *SMG1*, *SNAPC1*, *SOWAHC*, *SPN*, *SRSF10*, *ST3GAL1*, *STX3*, *STNRG*, *TBRG1*, *TCEANC2*, *TEP1*, *UBE2G2*, *VWA2*, *WDCP*, *WDR92*, *XAF1*, *XIAP*, and *YAF2* (Tables S1–S19).

Genes marked with ** contain BmiRNA binding sites in the 5′UTR. Figure 5 illustrates the predicted interactions of BmiR-531-5p, BmiR-1641-5p, and BmiR-1608-5p with the 3′UTR of the *ACTR5* mRNA.

Among the genes listed above, *DFFA*, *KCNA7*, and *UBE2G2* are notable because their mRNAs each contain two BS clusters for BmiR-531-5p, BmiR-1641-5p, and BmiR-1608-5p. In the 3′UTR of the *DFFA* mRNA, two identical BS clusters are present, with their starting positions separated by 302 nucleotides (nt). In the *KCNA7* mRNA, the corresponding clusters are separated by 413 nt, whereas in the *UBE2G2* mRNA, they are separated by 1037 nt. No binding site clusters for these three BmiRNAs were identified within the CDSs of the analyzed target genes. Among all target genes examined, only the *ADAL* mRNA contains binding sites for BmiR-531-5p, BmiR-1641-5p, and BmiR-1608-5p within its 5′UTR (Figure 6).

A characteristic feature of this BmiRNA group is that all three BmiRNAs are derived from the 5′ arm of their precursor hairpin structures (BmiRNA-5p). This common origin suggests that strand selection during BmiRNA biogenesis may contribute to their functional properties. It is conceivable that discrimination between BmiRNA-5p and BmiRNA-3p occurs during, or immediately before, the loading of the pre-BmiRNA-derived duplex into Argonaut proteins.

A group comprising BmiR-1441-3p, BmiR-1189-3p, and BmiR-2289-3p was predicted to form BS clusters within the mRNAs of 45 target genes, including *GGCX*, *GLRX3*, *GNF*, *GSDMA*, *HUNK*, *ICK*, *KLF8*, *LDHAL6A*, *LIN28A*, *LNX2*, *LOC100996*, *MOAP1*, *MPV17L*, *MTX3*, *MUC20*, *NDUFV3*, *NPAP1*, *PAPOLA*, *PCDHB16*, *PEX2*, *PEX26*, *PLEKHA2*, *POU5F1*, *PPM1K*, *RPP30*, *RTN4IP1*, *SEC62*, *SERBP1*, *SFXN1*, *SLC7A14*, *SPOP*, *SPRYD4*, *STEAP2*, *STX1B*, *TADA3*, *TERF2*, *TTC39C*, *TUFT1*, *TXNL4B*, *UBE2G1*, *ULK2*, *USP9X*, *VHL*, *VPS53*, *XKR5*, and *XPNPEP3* (Tables S7–S19).

Figure 7 illustrates the predicted interactions of BmiR-1441-3p, BmiR-1189-3p, and BmiR-2289-3p with the *LNX2* mRNA. Among the target genes identified for this BmiRNA group, only a single BS cluster was detected within the 5′UTR (Figure 8). In addition, binding sites for these three BmiRNAs were identified within the CDS of the *LOC100996* mRNA (Figure 9).

The BmiRNA group comprising BmiR-1441-3p, BmiR-2164-3p, and BmiR-1189-3p was predicted to bind to the mRNAs of 21 target genes, including *ACUL1*, *APAF1*, *BMP8A*, *BCL2L15*, *C2CD4A*, *MRPS27*, *CACUL1*, *CCL16*, *CD3EAP*, *CENPI*, *CHP1*, *COX18*, *CPPED1*, *CPSF2*, *EEF2K*, *FAM219A*, *FCAR*, *FKBP5*, *GATC*, and related targets. Figure 10 illustrates the predicted interactions of BmiR-1441-3p, BmiR-2164-3p, and BmiR-1189-3p with the *C2CD4A* mRNA.

Among these target genes, the *CHP1* mRNA contains two BS clusters of binding sites for BmiR-1441-3p, BmiR-2164-3p, and BmiR-1189-3p. The two clusters are separated by 344 nt, suggesting the presence of multiple potential regions where these three BmiRNAs may compete for binding.

A cluster in the 5′UTR of the *CENPI* gene mRNA was identified for BmiR-1441-3p, BmiR-2164-3p, and BmiR-1189-3p. The interaction patterns of these BmiRNAs are shown in Figure 11.

### 2.3. Single BmiRNA Binding

Particularly noteworthy are BmiRNAs whose binding sites are located outside BS clusters, allowing them to regulate target gene expression without direct competition from other BmiRNAs. One such example is BmiR-754-5p, which was predicted to interact with the mRNAs of 69 target genes, including *AAK1*, *ABCB5*, *ABHD2*, *ABHD11*, *ADM2*, *AGMAT (two BSs)*, *AIPL1*, *ANAPC16*, *AP1S3*, *AP3M1*, *AP5B1*, *ARL11*, *AS3MT*, *ATAD3C*, *ATCAY*, *BCL2L15*, *BMP7*, *BRCA1*, *C12orf65*, *C14orf132*, *C4orf34*, *C5orf28*, *C6orf225*, *CC2D1B*, *CCDC137*, *CD68*, *CDHR3*, *CENPL*, *CEP104*, *CHST6*, *CLN8*, *CMBL*, *CNNM1*, *CORO2A*, *CPM*, *CPPED1*, *CRKL*, *CRX*, *CSNK1G1*, *CXorf36*, *CYCS*, *CYP20A1*, *CYP27C1*, *DBT*, *DCAF*, *DCUN1D2*, *DENND6A*, *DESI1*, *DLGAP4*, *DNAJC18*, *DNASE2*, *DSTYK*, *EMC1*, *EMP2*, *EPGN*, *FAM126B*, *FAM200A*, *FBLIM1*, *FBXL13*, *FBXL20*, *FBXO27*, *FGD4*, and *FKBP14* (Tables S1–S6). Interestingly, no BmiR-754-5p binding sites were identified in the mRNAs of genes listed in the tables whose names begin with the letter G (Table S7).

Among all BmiRNAs analyzed, BmiR-531-5p exhibited the broadest predicted target spectrum, with binding sites identified in the mRNAs of 404 human genes. In most target genes, BmiR-531-5p is represented by a single binding site. However, in many mRNAs, it forms BS clusters together with BmiR-1641-5p, as described above. The *ORAI2* mRNA is particularly remarkable because it contains 17 BmiRNA binding sites, including six pairs of overlapping binding sites for BmiR-531-5p and BmiR-1641-5p.

The results of this study demonstrate the remarkable diversity of BmiRNA interactions with target gene mRNAs. At first glance, it might be expected that a single BmiRNA would be sufficient to regulate the expression of an individual target gene. However, our analysis revealed that some genes contain numerous BmiRNA binding sites. For example, the *ORAI2* mRNA harbors 17 predicted BmiRNA binding sites, raising the question of the functional significance of such an extensive regulatory architecture. One possible explanation is that these multiple binding sites serve as a reservoir for BmiRNAs, allowing their temporary sequestration and subsequent release under specific cellular conditions to regulate the expression of additional target genes.

The mRNA of the *BMP8A* gene has six binding sites for all different BmiRNAs. The mRNA of the *CBX5* gene has six binding sites, three of which belong to miR-2289-3p located in the 3′UTR at positions 4483 nt, 7059 nt, and 8494 nt. The mRNA of the *CFLAR* gene has eight binding sites, of which BmiR-531-5p and BmiR-1641-5p have two sites each. The mRNA of the *CYCS* gene has seven binding sites for different BmiRNAs. The mRNA of the *CYP20A1* gene has eight binding sites, of which BmiR-531-5p and BmiR-1641-5p have two sites each. The mRNA of the *FBXO27* gene has six binding sites, of which miR-531-5p and BmiR-1641-5p have two binding sites each. The mRNA of the *FKBP5* gene has eight BSs, of which BmiR-1441-3p, BmiR-2164-3p, BmiR-1189-3p, and BmiR-2289-3p have two BSs each. The mRNA of the *FOXK1* gene has seven BSs, of which BmiR-1169-3p and BmiR-2289-3p have two BSs each. The mRNA of the *NMT2* gene has eight BSs, of which BmiR-531-5p and BmiR-1641-5p have three BSs each. The mRNA of the *NOL9* gene has six BSs, of which all BmiRNAs have one BS each. The mRNA of the *ORAI2* gene has six BSs for BmiR-531-5p and six BSs for BmiR-1641-5p, which are paired in the 3′UTR in clusters of 2624 nt, 4032 nt, 4104 nt, 5198 nt, 8302 nt, and 9089 nt. The BS of miR-1608-5p is adjacent to the BS pair localized at the site starting with 9091 nt. The BSs for BmiR-1169-3p and BmiR-1379-5p are located at positions 6961 nt and 10,393 nt, respectively. As a result, the *ORAI2* gene with such connections with BmiRNAs is a great mystery of nature. The mRNA of the *QPCTL* gene has BSs for BmiR-531-5p, BmiR-1641-5p, and BmiR-1608-5p in one cluster; BmiR-531-5p and BmiR-1641-5p in another cluster and BSs for BmiR-1169-3p. The mRNA of the *PARD6B* gene has eight BSs which are divided into three clusters; in one they bind BmiR-531-5p and BmiR-1641-5p, in another BmiR-1441-3p and BmiR-1189-3p, and in the third BmiR-1169-3p and BmiR-2289-3p. The mRNA of the *PDDC1* gene has seven BSs which are divided into three clusters, in one BmiR-1441-3p, BmiR-1189-3p, and BmiR-2289-3p and in another BmiR-531-5p, 1641-5p and BmiR-1608-5p 1919. The mRNA of the *PHACTR4* gene has eight BSs that are divided into three clusters; in one they bind BmiR-531-5p (3797 nt) and BmiR-1641-5p (3798 nt); in another BmiR-531-5p (4869 nt), 1608-5p (4871 nt) and BmiR-1641-5p (4870 nt); and in the third BmiR-1608-5p (3333 nt) and BmiR-1641-5p (3332 nt). The BS of BmiR-1927-5p (5055 nt) is located last. The mRNA of the *SLC24A4* gene has six BSs that are divided into three clusters: one with BmiR-1441-3p and BmiR-1189-3p, another with BmiR-1169-3p and BmiR-2289-3p, and the third with BmiR-1169-3p and BmiR-2289-3p. The mRNA of the *SLC9A7* gene has eight BSs which are divided into three clusters, one with BmiR-1441-3p, BmiR-1189-3p and BmiR-2289-3p and the other with BmiR-1441-3p, BmiR-1189-3p and BmiR-2289-3p, and BmiR-1927-5p and BmiR-2289-3p have independent binding sites. The mRNA of the SSH1 gene has six BSs for cluster BmiR-531-5p and BmiR-1641-5p (7608 nt, 7609 nt) and for cluster BmiR-531-5p and BmiR-1641-5p (7775 nt, 7776 nt). BmiR-1441-3p and BmiR-1189-3p have their own cluster of BSs. The mRNA of the *TBC1D24* gene has six BSs which are divided into three clusters; in one they bind BmiR-1441-3p, BmiR-1189-3p and BmiR-2289-3p; in another BmiR-1441-3p and BmiR-2289-3p; and in the third BmiR-1169-3p and BmiR-2289-3p. The mRNA of the *UBE2G2* gene has six BSs of which BmiR-531-5p, BmiR-1641-5p and BmiR-1608-5p have two BSs each. The mRNA of the *VPS53* gene has seven BSs, of which BmiR-531-5p and BmiR-1641-5p have two BSs each. The mRNA of the *XPNPEP3* gene has seven BSs, of which only BmiR-1641-5p has two BSs.

### 2.4. Binding of BmiRNA-5p and BmiRNA-3p

A rare example of paired BmiRNA interactions was identified for BmiR-2455-5p and BmiR-2455-3p, both of which were predicted to bind the *CACNA2D4* mRNA. Their binding sites are located in the 5′UTR and 3′UTR, respectively, and are separated by 38 nucleotides (Figure 12). A similar pattern was observed for the BmiR-864-3p/BmiR-864-5p pair, whose binding sites were identified within the CDS of the *ARGLU1* mRNA.

## 3. Discussion

The results of this study indicate that BmiRNAs can suppress the translation of mRNA target genes. However, there are a number of properties of BmiRNAs and their target genes that are difficult to explain but undoubtedly necessary. The clusters bind BmiRNAs with the same pre-miRNA origin, i.e., only BmiRNA-5p or onlyBmiRNA-3p.

A constant property of BmiRNA-mRNA interactions is that only one type of BmiRNA-5p or BmiRNA-3p binds to miRNA-binding clusters. MiRNA binding to Argonauts occurs through electrical interactions, as Argonaut proteins contain an excess of positively charged amino acids. The non-interacting portions of the miRNA hairpin are truncated, and this action determines the length of the miRNA’s active region. The Argonaut-bound miRNA then interacts with the mRNA via hydrogen bonds across the entire miRNA sequence. The energy of this interaction is higher than that of the electrical interaction, which determines the direction of the process and the stability of the BmiRNA-mRNA complex. It is currently unknown how the binding site clusters distinguish miRNA-5p from BmiRNA-3p. It is possible that these BmiRNAs differ in their opposite helical structures.

It remains unclear why multiple BmiRNAs can bind within the BS cluster. This suggests the duplication of mRNA translation suppression. It is even less clear why identical BmiRNA groups bind to the mRNAs of multiple genes. One explanation for this phenomenon is that the miRNA group controls the expression of these genes.

The results obtained in this study significantly advance our understanding of the function of the miRNAs studied and, in combination with other miRNAs that interact fully complementarily with the mRNAs of multiple genes, demonstrate their important role in regulating the expression of the protein-coding region of the human genome.

The results of this study indicate that BmiRNAs are capable of suppressing the translation of target gene mRNAs. However, there are certain properties of BmiRNAs and their target genes that are difficult to explain yet require elucidation. Binding site clusters comprise either BmiRNAs originating from the same pre-BmiRNA or exclusively BmiRNA-5p or BmiRNA-3p species. A characteristic feature of BmiRNA interactions is that only one type of BmiRNA—either BmiRNA-5p or BmiRNA-3p—binds to the clusters. The binding of BmiRNAs to Argonaut family proteins is mediated by electrostatic interactions, as these proteins contain an excess of positively charged amino acids. The non-interacting segments of the BmiRNA strand are cleaved off, and it is precisely this process that determines the length of the active BmiRNA region. The BmiRNA, bound to the Argonaut protein, interacts with mRNA via hydrogen bonds along the entire length of its sequence. The energy of this interaction exceeds that of the electrostatic interaction, thereby determining the direction of the process and the stability of the BmiRNA–mRNA complex. It is currently unknown how binding site clusters distinguish between BmiRNA-5p and BmiRNA-3p. It is possible that these BmiRNAs differ in their helical structure (possessing opposite helical configurations).

It remains unclear why multiple BmiRNAs bind within a cluster of binding sites; this suggests a redundancy in the mechanism of mRNA translation suppression. It is also unclear why identical groups of BmiRNAs bind to the mRNAs of numerous different genes. One possible explanation is that a given group of BmiRNAs controls the expression of these genes.

## 4. Materials and Methods

The nucleotide (nt) sequences of 17,508 human genes were downloaded from National Center for Biotechnology Information (NCBI https://www.ncbi.nlm.nih.gov, 2022). The calculations used gene isoforms with the longest nucleotide sequences. The nucleotide sequences of 1036 BmiRNAs were taken from Backes et al. [2]. The BmiRNA binding sites (BSs) in mRNA were predicted using the MirTarget program [21]. This program predicts the following features of miRNA interaction with mRNA: (a) the initiation of miRNA binding to the mRNA from the first nucleotide of the mRNA; (b) the localization of the miRNA BSs in the 5′-untranslated region (5′UTR), coding domain sequence (CDS), and 3′-untranslated region (3′UTR) of the mRNAs; (c) the schemes of nucleotide interactions between miRNA with mRNA, which clearly demonstrate the interactions of all nucleotides between miRNA with mRNA; and (d) the Gibbs free energy (ΔG, kJ/mol) of the interaction between miRNA and the mRNA. The ratio ΔG/ΔGm (%) is determined for each site. ΔGm equals the free energy of miRNA binding with its fully complementary canonical nucleotide sequence. Only miRNAs whose nucleotides interacted with mRNA using canonical (G-C and A-U) nucleotides with a given ΔG value were selected from the calculated data [22,23]. The MirTarget program finds hydrogen bonds between miRNA and mRNA according to the physicochemical characteristics of nucleotide interactions. MirTarget differs from other programs in terms of finding miRNA BSs on mRNA in the following respects: it takes into account the interaction of miRNA with mRNA over the entire miRNA nucleotide sequence, and it calculates the free energy of the interaction of the miRNA with mRNA. Note that the G, A, C, and U nucleotides, which comprise the RNA structure of microorganisms, plants, and animals, interact identically under equal conditions. Therefore, considering the physicochemical properties of the canonical nucleotide pairs given above, additional proof of the previously established physicochemical characteristics of their interaction is not required. The reliability of translation suppression by miRNAs that are fully complementary to mRNAs was proven by A. Fire and C.C. Mello, who were awarded the Nobel Prize in 2006 for this research [24]. This year’s Nobel Prize focuses on the discovery of a vital regulatory mechanism used in cells to control gene activity. Genetic information flows from DNA to messenger RNA (mRNA), via process transcription, and then to the cellular machinery for protein production.

## 5. Conclusions

The results of this study significantly expand our understanding of the properties of the BmiRNAs investigated. Together with data on other BmiRNAs that interact with the mRNAs of multiple genes via perfect complementarity, these findings demonstrate the important role BmiRNAs play in regulating the expression of the protein-coding regions of the human genome. The most significant aspect of these results is the establishment of a definitive link between each BmiRNA and its target genes—and, conversely, the identification of the specific BmiRNA that regulates the expression of a given gene. A key strength of this study is the establishment of a definitive link between a specific miRNA and one or more target genes. This work presents the quantitative characteristics of miRNA–mRNA interactions and identifies which miRNAs compete with one another. Previously, these miRNA properties could not be determined due to the lack of methods for analyzing miRNA–mRNA interactions. The data obtained will significantly accelerate the elucidation of the functional roles of both individual miRNAs and groups of miRNAs [25,26,27].

## Figures and Tables

**Figure 1:**
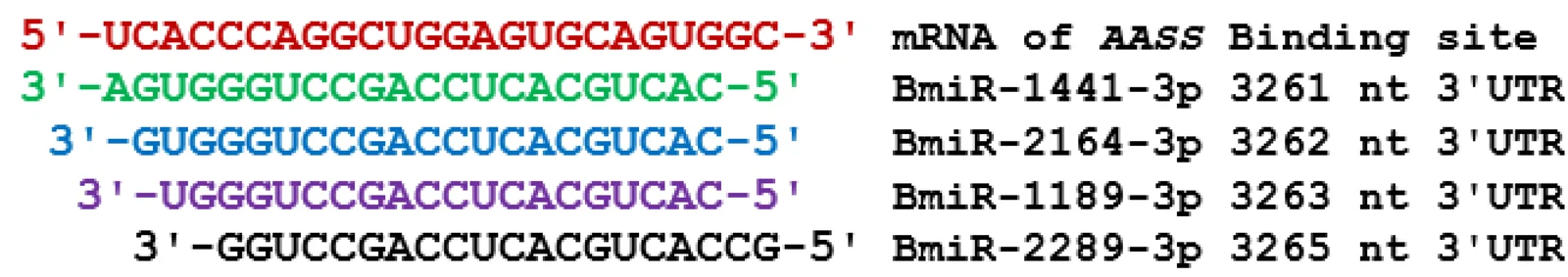
Schemes of the interaction of BmiR-1441-3p, BmiR-2164-3p, BmiR-1189-3p, and BmiR-2289-3p with the 3′UTR mRNA of the *AASS* gene. Here and below, the nucleotide sequence of mRNA is shown in red, and the corresponding BmiRNAs are shown in other colors.

**Figure 2:**
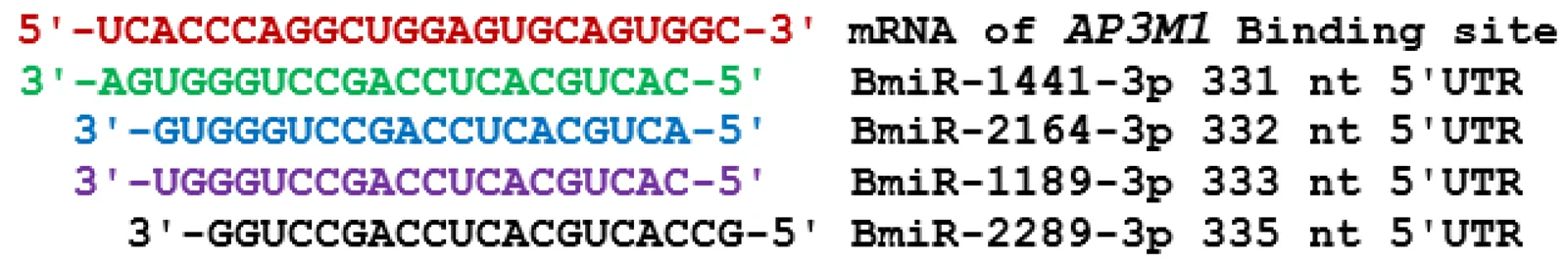
Schemes of the interaction of BmiR-1441-3p, BmiR-2164-3p, BmiR-1189-3p, and BmiR-2289-3p with the 5′UTR mRNA of the *AP3M1* gene.

**Figure 3:**
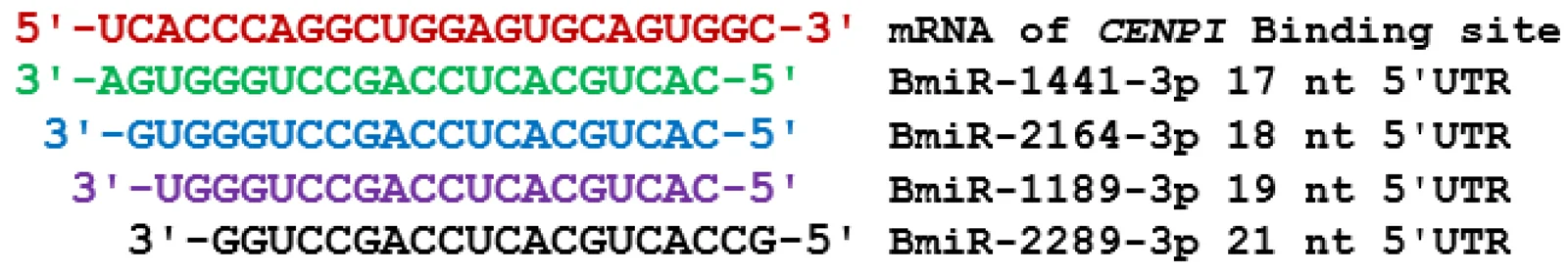
Schemes of the interaction of BmiR-1441-3p, BmiR-2164-3p, BmiR-1189-3p, and BmiR-2289-3p with the 5′UTR mRNA of the *CENPI* gene.

**Figure 4:**
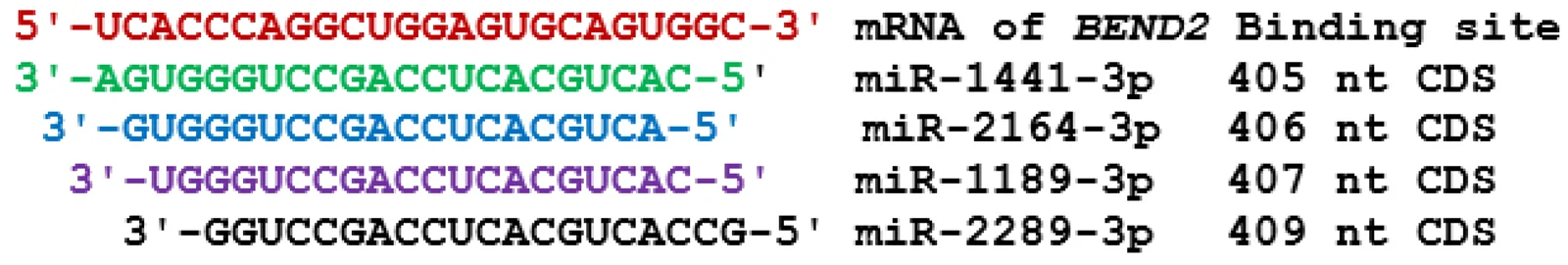
Schemes of the binding of BmiR-1441-3p, BmiR-2164-3p, BmiR-1189-3p, and BmiR-2289-3p in the CDS of the mRNA of the *BEND2* gene.

**Figure 5:**
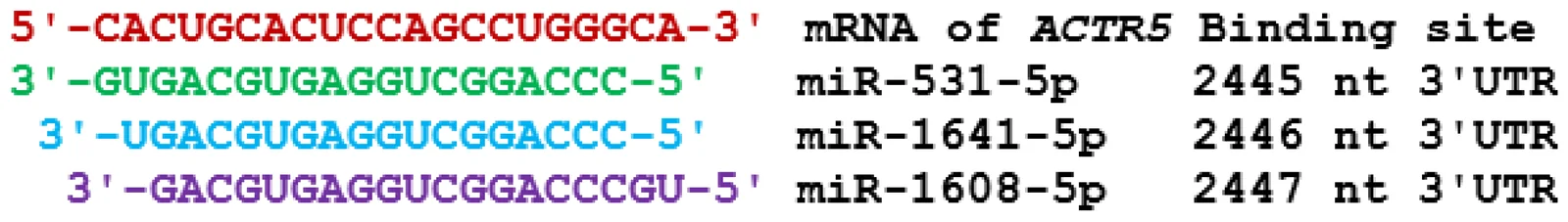
Schemes of the binding of BmiR-531-5p, BmiR-1641-5p, and BmiR-1608-5p in the 3′UTR of the mRNA of the *ACTR5* gene.

**Figure 6:**
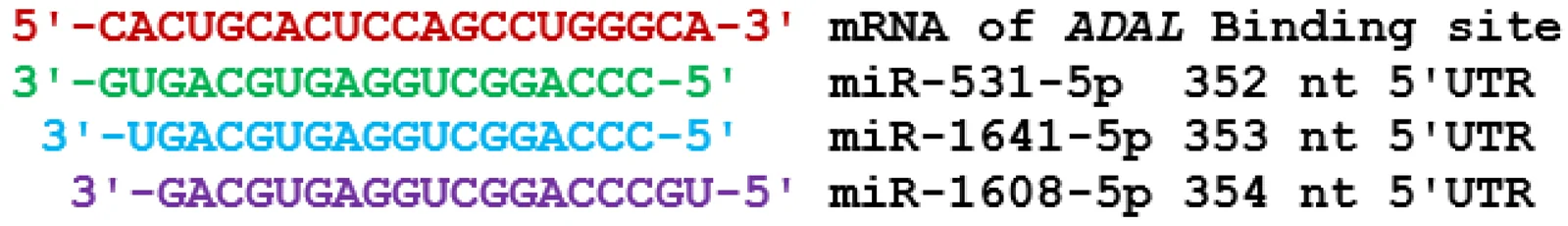
Schemes of the binding of BmiR-531-5p, BmiR-1641-5p, and BmiR-1608-5p in the 5′UTR of the mRNA of the *ADAL* gene.

**Figure 7:**
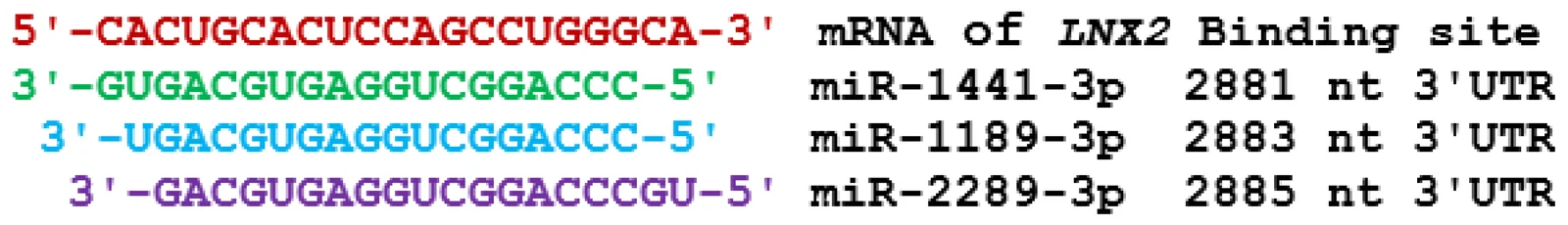
Schemes of the binding of BmiR-1441-3p, BmiR-1189-3p, and BmiR-2289-3p in the 3′UTR of the mRNA of the *LNX2* gene.

**Figure 8:**
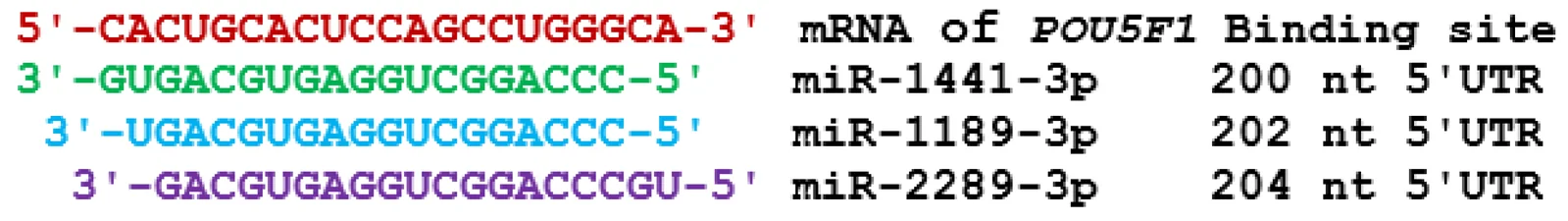
Schemes of the binding of BmiR-1441-3p, BmiR-1189-3p, and BmiR-2289-3p in the 5′UTR of the mRNA of the *POU5F1* gene.

**Figure 9:**
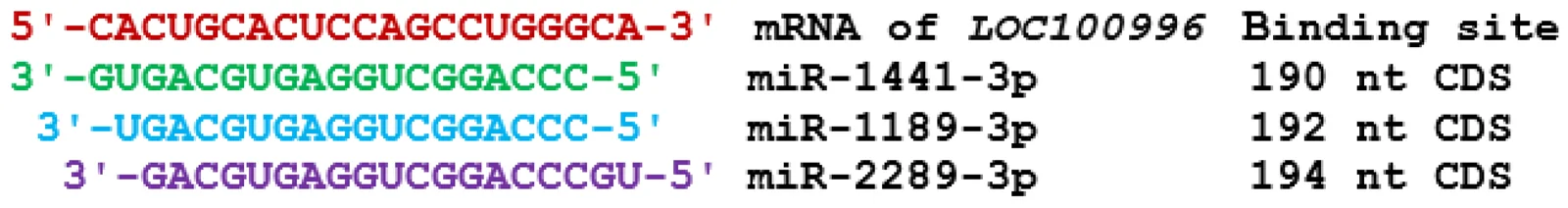
Schemes of the binding of BmiR-1441-3p, BmiR-1189-3p, and BmiR-2289-3p in the CDS of the mRNA of the *LOC100996* gene.

**Figure 10:**
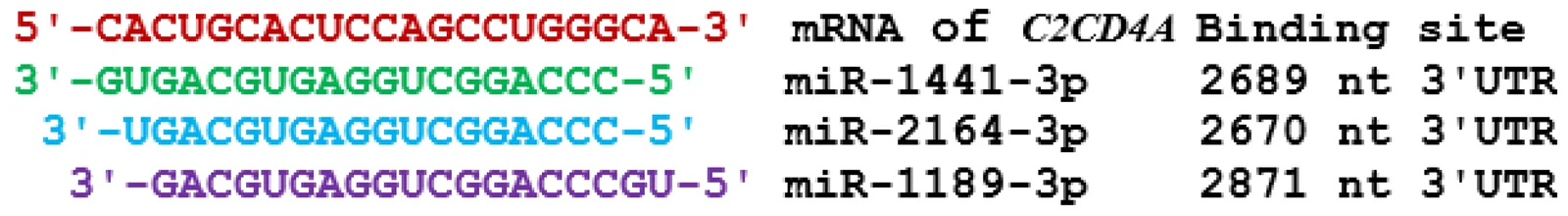
Schemes of the binding of BmiR-1441-3p, BmiR-2164-3p, and BmiR-1189-3p in the 3′UTR of the mRNA of the *C2CD4A* gene.

**Figure 11:**
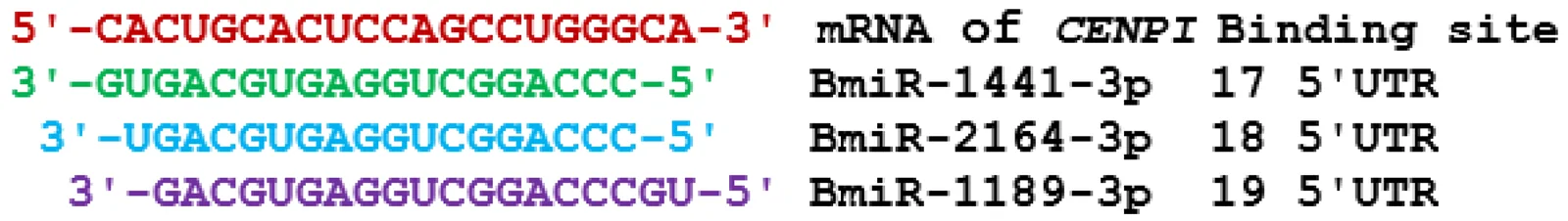
Schemes of the binding of BmiR-1441-3p, BmiR-2164-3p, and BmiR-1189-3p in the 5′UTR mRNA of the *CENPI* gene.

**Figure 12:**
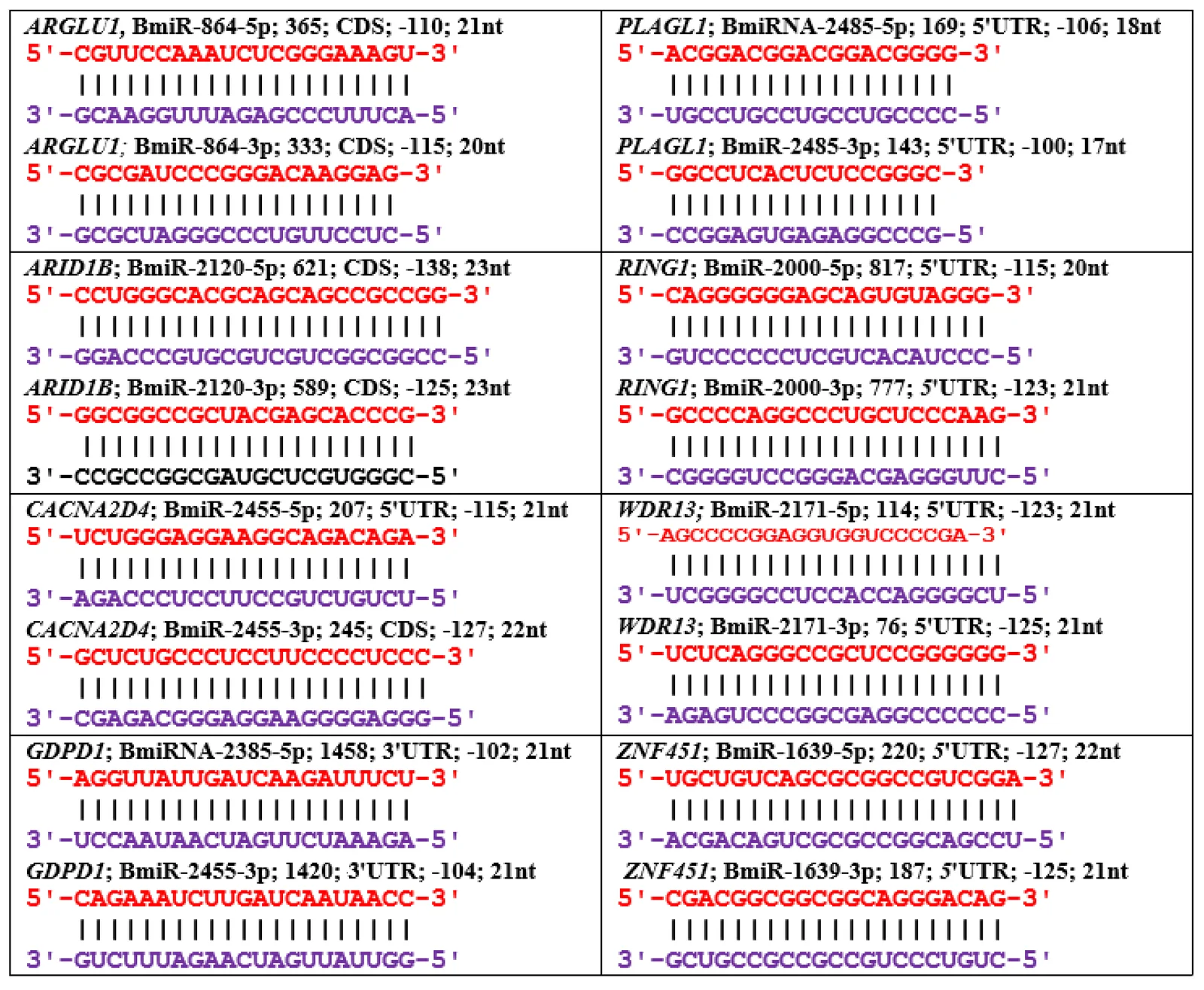
Schemes of BmiRNA-5p and BmiRNA-3p binding to gene mRNAs.

## Data Availability

The nucleotide sequences of 17,508 genes were downloaded from National Center for Biotechnology Information (NCBI) (https://www.ncbi.nlm.nih.gov, 2022). The nucleotide sequences of 1036 miRNAs were taken from NCBI (http://mirbase.org).

## Supplementary Materials

The following supporting information can be downloaded at https://www.mdpi.com/article/10.3390/ijms27198773/s1.

## Abbreviations

The following abbreviations are used in this manuscript:

3′UTRs	3′-untranslated regions
5′UTRs	5′-untranslated regions
CDS	Coding sequence
