# Supplementary material for: Features of Complementary Interactions of BmiRNAs with mRNAs of Human Genes
Source: Int J Mol Sci. 2026 Sep 30;27(19):8773. doi: 10.3390/ijms27198773 (PMC13665148; doi:10.3390/ijms27198773)
Supplement: Supplementary file 1 [file ijms-27-08773-s001.zip › Table S1.pdf]

Table S1. Quantitative Characteristics of BmiRNA Binding to mRNAs of Genes Beginning with “A”.

Gene; BmiRNA; BS,nt; ΔG, kj/mole; BmiRNA length, nt.

A2ML1; BmiR-531-5p; 5039; -108; 19	*APAF1*;BmiR-2289-3p;5232;-115;20
A2ML1; BmiR-1641-5p; 5040; -102;18	*APOBEC3A*;BmiR-1641-5p;1179;-102;18
A2ML1;BmiR-1608-5p;5041; -108;19	*APOBEC3D*;BmiR-1441-3p;33**;-123;22
AAK1;BmiR-754-5p;15533; -119;22	*APOBEC3D*;BmiR-2164-3p;34**;-113;20
AANAT;BmiR-1169-3p;732**;-115;20	*APOBEC3D*;BmiR-1189-3p;35**;-113;20
AARD;BmiR-531-5p;745; -108;19	*APOBEC3D*;BmiR-2289-3p;37**;-115;20
AARD;BmiR-1641-5p;746; -102;18	*APOBEC3F*;BmiR-1791-5p;1801;-102;19
AARS2;BmiR-531-5p;3470; -108;19	*APOL1*;BmiR-531-5p;2350;-108;19
AARS2;BmiR-1641-5p;3471; -102;18	*APOL1*;BmiR-1641-5p;2351; 102;18
AASS;BmiR-1189-3p;3263; -113;20	*APOL6*;BmiR-1169-3p;1535;-115;20
AASS;BmiR-1441-3p;3261; -123;22	*APOL6*;BmiR-2289-3p;1537;-115;20
AASS;BmiR-2164-3p;3262; -113;20	*APOOL*;BmiR-1169-3p;2296;-115;20
AASS;BmiR-2289-3p;3265;;-115;20	*APOOL*;BmiR-2289-3p;2298;-115;20
ABCA13;BmiR-1189-3p;16248; -113;20	*APPBP2*;BmiR-1927-5p;5559;-113;19
ABCA13;BmiR-1441-3p;16246; -123;22	*APPL1*;BmiR-1169-3p;5229;-115;20
ABCA13;BmiR-2164-3p;16247; -113;20	*AQP6*;BmiR-1189-3p;1789;-113;20
ABCA13;BmiR-2289-3p;16250;;-115;20	*AQP6*;BmiR-2289-3p;1791;-115;20
ABCA5;BmiR-1096-3p;44**;-108;20	*ARFIP2*; BmiR-531-5p; 2791; -108;; 19
ABCB5;BmiR-754-5p;4833; -119;22	*ARFIP2*; BmiR-1641-5p; 2792; -102; 18
ABCB5;BmiR-2086-3p;4975;-138;25	*ARFIP2*; BmiR-1608-5p; 2793; -108; 19
ABHD; BmiR-1641-5p4 296; -102; 18	*ARGFX*;BmiR-1189-3p;2141;-113;20
ABHD; BmiR-531-5p; 4295; -108; 19	*ARGFX*;BmiR-1441-3p;2139;-123;22
ABHD; BmiR-1131-5p; 4296; -102; 19	*ARGFX*;BmiR-2164-3p;2140;-113;20
ABHD2;BmiR-754-5p;7658; -119;22	*ARGFX*-;BmiR-2289-3p;2143;-115;20
ABHD2;BmiR-1608-5p;7816; -108;19	*ARGLU1*;BmiR-864-3p;333*;-115;20
ABHD5;BmiR-531-5p;4295; -108;19	*ARGLU1*;BmiR-864-5p;365*;-110;21
ABHD5;BmiR-1641-5p;4296; -102;18	*ARHGAP11A*;BmiR-2289-3p;4069;-115;20
ABHD11;BmiR-754-5p;1110; -119;22	*ARHGAP26*;BmiR-1169-3p;5261;-115;20
ABHD15;BmiR-531-5p;3313; -108;19	*ARHGEF39*;BmiR-2087-3p;3152;-102;19
ABHD15;BmiR-1641-5p;3314; -102;18	*ARID1B*v;BmiR-2120-3p;589;-125;23
ABL2; BmiR-531-5p; 9484; -108; 19	*ARID1B*;BmiR-2120-5p;621*;-138;23
ABL2; BmiR-1641-5p; 9485; -102; 18	*ARIH2OS*;BmiR-531-5p;1522;-108;19
ABL2; BmiR-754-5p; 9969; -119; 22	*ARIH2OS*;BmiR-1641-5p;1523;-102;18
ACAP2;BmiR-1189-3p;3847; -113;20	*ARL11*;BmiR-754-5p;1532;-119;22
ACAP2;BmiR-2164-3p;3846; -113;20	*ARL17A*;BmiR-2087-3p;4448;-102;19
ACAP2;BmiR-2289-3p;3849; -115; 20	*ARNTL2*;BmiR-1169-3p;4967;-115;20
ACBD7;BmiR-531-5p; 2284; -108; 19	*ARNTL2*;BmiR-2289-3p;4969;-115;20
ACBD7; BmiR-1641-5p; 2285; -102;18	*ARPP19*;BmiR-1169-3p;3932;-115;20
ACBD7; BmiR-2164-3p; 993; -113; 20	*ARSA*;BmiR-1096-3p;3363;-108;20
ACSL6;;BmiR-1096-3p;3091; -108;20	*ARSA*;BmiR-1927-5p;3230;-113;19
ACTR5; BmiR-531-5p; 2445; -108; 19	*ARSB*;BmiR-1096-3p;5625;-108;20
ACTR5; BmiR-1641-5p;2446; -102; 18	*ARSB*;BmiR-1189-3p;5455;-113;20
ACTR5; BmiR-1608-5p; 2447; -108; 19	*ARSB*;BmiR-1441-3p;5453;-123;22
ADAL; BmiR-531-5p; 352**; -108; 19	*ARSB*;BmiR-2164-3p;5454;-113;20
ADAL; BmiR-1641-5p; 353**; --102; 18	*ARSD*;BmiR-1927-5p;2096;-113;19
ADAL; BmiR-1608-5p; 354**; -108; 19	*AS3MT*;BmiR-754-5p;1455;-119;22
ADAM33;BmiR-531-5p;3455; -108;19	*ASB11*;BmiR-531-5p;2740;-108;19
ADAM33;BmiR-1641-5p;3456;-102;18	*ARIH2OS*;BmiR-1641-5p;1523;-102;18
ADAMTS17; BmiR-1169-3p; 5540; -115; 20	*ARL11*;BmiR-754-5p;1532;-119;22
ADAMTS17; BmiR-2289-3p; 5542; -115; 20	*ARL17A*;BmiR-2087-3p;4448;-102;19
ADAMTS17; BmiR-2083-3p; 5764; -106; 20	*ARNTL2*;BmiR-1169-3p;4967;-115;20
ADARB1;BmiR-1189-3p;1846*;-113;20	*ARNTL2*;BmiR-2289-3p;4969;-115;20
ADARB1;BmiR-2164-3p;184*;-113;20	*ARPP19*;BmiR-1169-3p;3932;-115;20
ADAT2;BmiR-1379-5p;3744;-102;19	*ARSA*;BmiR-1096-3p;3363;-108;20

ADCY2;BmiR-531-5p;6511;-108 ;19	*ARSA*;BmiR-1927-5p;3230;-113;19
ADCY2;BmiR-1641-5p;6512 ;-102;18	*ARSB*;BmiR-1096-3p;5625;-108;20
ADM2;BmiR-754-5p(;2809 ;-119;22	*ARSB*;BmiR-1189-3p;5455;-113;20
ADRA1A;;BmiR-2289-3p;1725*;-115;20	*ARSB*;BmiR-1441-3p;5453;-123;22
ADRBK2;BmiR-531-5p;5672;-108;19	*ARSB*;BmiR-2164-3p;5454;-113;20
A*DRBK2*;BmiR-1641-5p;5673;-102;18	*ARSD*;BmiR-1927-5p;2096;-113;19
AFF3; BmiR-1169-3p; 5828; -115; 20	*AS3MT*;BmiR-754-5p;1455;-119;22
AFF3; BmiR-2289-3p; 5830; -115; 20	*ASB11*;BmiR-531-5p;2740;-108;19
AFF3; BmiR-1379-5p; 6828; -102; 19	*ASB16*; BmiR-1169-3p; 1568; -115; 20
AIFM2; BmiR-1441-3p; 2007; -123; 22	*ASB16*; BmiR-2289-3p; 1570; -115; 20
AIFM2; BmiR-2164-3p; 2008; -113; 20	*ASB16*; BmiR-1096-3p; 2722; -108; 20
AIFM2; BmiR-2289-3p; 2011; -115; 20	*ASRGL1*; BmiR-531-5p; 1532; -108; 19
AFMID;BmiR-1189-3p;1367;-113;20	*ASRGL1*; BmiR-1641-5p; 1533; -102; 18
AFMID;BmiR-1441-3p;1365;-123;22	*ASRGL1*; BmiR-1608-5p; 1534; -108; 19
AFMID;BmiR-2164-3p;1366;-113;20	*ATAD3C*; BmiR-531-5p; 596**; -102; 18
AFMID;BmiR-2289-3p;1369;-115;20	*ATAD3C*; BmiR-1641-5p; 597**; -102; 18
AGMAT;BmiR-531-5p;1975;-10819	*ATAD3C*; BmiR-754-5p; 2766; -119; 22
AGMAT;BmiR-754-5p;2279 ;-119;22	*ATCAY*;BmiR-531-5p;3219;-108;19
AGMAT;BmiR-754-5p;2578;-119;22	*ATCAY*;BmiR-531-5p;4134;-108;19
AGMAT;BmiR-1641-5p;1976;-102;18	*ATCAY*;BmiR-754-5p;2764;-119;22
AGMAT;BmiR-2289-3p;2732;-115;20	*ATCAY*;BmiR-1608-5p;3221;-108;19
AGMO; BmiR-2087-3p; 2189; -102; 19	*ATCAY*;BmiR-1641-5p;3220;-102;18
AGMO; BmiR-531-5p; 2241; -108; 19	*ATCAY*;BmiR-1641-5p;4135;-102;18
AGMO; BmiR-1641-5p; 2242; -102; 18	*ATF6*;BmiR-531-5p;3105;-108;19
AIFM2;BmiR-1189-3p;2009;-113;20	*ATF6*;BmiR-1641-5p;3106;-102;18
AIFM2;BmiR-1441-3p;2007;-123;22	*ATF7IP2*;BmiR-1096-3p;2951;-108;20
AIFM2;BmiR-2164-3p;2008;-113;20	*ATG12*;BmiR-1096-3p;2604;-108;20
AIFM2;BmiR-2289-3p;2011;-115;20	*ATM*;BmiR-1189-3p;11075;-113;20
AIPL1;BmiR-754-5p;1931;-119;22	*ATM*;BmiR-1441-3p;11073;-123;22
AK1;BmiR-531-5p;1677;-108;19	*ATM*;BmiR-2164-3p;11074;-113;20
AK1;BmiR-1641-5p;1678;-102;18	*ATM*;BmiR-2289-3p;11077;-115;20
AK3;BmiR-531-5p;3496;-108;19	*ATP1B2*;BmiR-1449-3p;2255;-115;21
AKAP12;BmiR-1449-3p;7101;-115;21	*ATP1B4*; BmiR-1441-3p; 3012; -123; 22
AKAP5;BmiR-1169-3p;3176;-115;20	*ATP1B4*; BmiR-2164-3p; 3013; -113; 20
AKAP5;BmiR-2289-3p;3178;-115;20	*ATP1B4*; BmiR-1189-3p; 3014; -113; 20
AKIP1;BmiR-1169-3p;905;-115;20	*ATP5F1*; BmiR-531-5p; 2058; -108; 19
AKIP1;BmiR-2289-3p;907;-115;20	*ATP5F1*; BmiR-1641-5p; 2059; -102; 18
AKIRIN1; BmiR-531-5p; 1928; -108; 19	*ATP5F1*; BmiR-1608-5p; 2060; -108; 19
AKIRIN1; BmiR-1641-5p; 1929; -102; 18	*ATP5J2*-PT;BmiR-531-5p;4251;-108;19
AKIRIN1; BmiR-1608-5p; 1930; -108; 19	*ATP5J2*-PT;BmiR-1641-5p;4252;-102;18
AKNA;BmiR-1927-5p;5825;-113;19	*ARIH2OS*;BmiR-1641-5p;1523;-102;18
ALDH1B1-;BmiR-1169-3p;2759;-115;20	*ARL11*;BmiR-754-5p;1532;-119;22
ALG1;BmiR-1441-3p;3041 ;-123;22	*ARL17A*;BmiR-2087-3p;4448;-102;19
ALG1;BmiR-1189-3p;3043;-113 ;20	*ARNTL2*;BmiR-1169-3p;4967;-115;20
ALG1;BmiR-2164-3p;3042 ;-113 ;20	*ARNTL2*;BmiR-2289-3p;4969;-115;20
ALG1;BmiR-2289-3p;3045;-115 ;20	*ARPP19*;BmiR-1169-3p;3932;-115;20
ALG1;BmiR-2289-3p;3267;-115 ;20	*ARSA*;BmiR-1096-3p;3363;-108;20
ALG8;BmiR-1449-3p;1432* ;-115 ;21	*ARSA*;BmiR-1927-5p;3230;-113;19
ALG10;BmiR-1297-3p;2113;-100 ;20	*ARSB*;BmiR-1096-3p;5625;-108;20
ALG10B;BmiR-1297-3p;1933 ;-100;20	*ARSB*;BmiR-1189-3p;5455;-113;20
ALOX15;BmiR-1189-3p;2393;-113;20	*ARSB*;BmiR-1441-3p;5453;-123;22
ALOX15;BmiR-2164-3p;2392 ;-113 ;20	*ARSB*;BmiR-2164-3p;5454;-113;20
ANAPC16;BmiR-754-5p;2961; -119;22	*ARSD*;BmiR-1927-5p;2096;-113;19
ANGEL2;BmiR-1169-3p;3544 ;-115 ;20	*AS3MT*;BmiR-754-5p;1455;-119;22
ANGEL2;BmiR-2289-3p;3546;-115;20	*ASB11*;BmiR-531-5p;2740;-108;19
ANKDD1A;BmiR-1927-5p;1545*;-113 ;9	*ASB16*; BmiR-1169-3p; 1568; -115; 20
ANKRD5;BmiR-1297-3p;3403;-100;20	*ASB16*; BmiR-2289-3p; 1570; -115; 20

ANKRD12;BmiR-531-5p;10524 ;-108;19	*ASB16*; BmiR-1096-3p; 2722; -108; 20
ANKRD16;BmiR-531-5p;2394;-108 ;19	*ASRGL1*; BmiR-531-5p; 1532; -108; 19
ANKRD16;BmiR-1608-5p;2396;-108;19	*ASRGL1*; BmiR-1641-5p; 1533; -102; 18
ANKRD16;BmiR-1641-5p;2395;-102;18	*ASRGL1*; BmiR-1608-5p; 1534; -108; 19
ANKRD62;BmiR-531-5p;3104;-108;19	*ATAD3C*; BmiR-531-5p; 596**; -102; 18
ANKRD62;BmiR-1641-5p;3105;-102;18	*ATAD3C*; BmiR-1641-5p; 597**; -102; 18
ANKS4B;BmiR-1096-3p;2620;-108;20	*ATAD3C*; BmiR-754-5p; 2766; -119; 22
AP1S3;BmiR-531-5p;2120;-108;19	*ATCAY*;BmiR-531-5p;3219;-108;19
AP1S3;BmiR-754-5p;3587;-119;22	*ATCAY*;BmiR-531-5p;4134;-108;19
AP1S3;BmiR-1641-5p;2121;-102;18	*ATCAY*;BmiR-754-5p;2764;-119;22
A*P1S3*;BmiR-2087-3p;2068;-102;19	*ATCAY*;BmiR-1608-5p;3221;-108;19
AP3M1;BmiR-754-5p;4778;-119;22	*ATCAY*;BmiR-1641-5p;3220;-102;18
AP3M1;BmiR-1441-3p;331**;-123;22	*ATCAY*;BmiR-1641-5p;4135;-102;18
AP3M1;BmiR-2164-3p;332**;-113;20	*ATF6*;BmiR-531-5p;3105;-108;19
AP3M1;BmiR-1189-3p;333**;-113;20	*ATF6*;BmiR-1641-5p;3106;-102;18
AP3M1;BmiR-2289-3p;335**;;-115;20	*ATF7IP2*;BmiR-1096-3p;2951;-108;20
AP3M1;BmiR-1361-5p;4933;-125;22	*ATG12*;BmiR-1096-3p;2604;-108;20
AP4E1;BmiR-1791-5p;5837;-102;19	*ATM*;BmiR-1189-3p;11075;-113;20
AP4S1;BmiR-531-5p;2700;;-108;19	*ATM*;BmiR-1441-3p;11073;-123;22
AP4S1;BmiR-1641-5p;2701;-102;18	*ATM*;BmiR-2164-3p;11074;-113;20
AP4S1;BmiR-531-5p;4196;-108;19	*ATM*;BmiR-2289-3p;11077;-115;20
AP4S1;BmiR-1641-5p;4197;-102;18	*ATP1B2*;BmiR-1449-3p;2255;-115;21
AP4S1;BmiR-1791-5p;1691;-102;19	*ATP1B4*; BmiR-1441-3p; 3012; -123; 22
AP5B1; BmiR-531-5p; 4544; -108; 19	*ATP1B4*; BmiR-2164-3p; 3013; -113; 20
AP5B1; BmiR-1641-5p; 4545; -102; 18	*ATP1B4*; BmiR-1189-3p; 3014; -113; 20
AP5B1; BmiR-754-5p; 5870; -119; 22	*ATP5F1*; BmiR-531-5p; 2058; -108; 19
APAF1;BmiR-1441-3p;5228;-123;22	*ATP5F1*; BBmiR-1641-5p; 2059; -102; 18
APAF1;BBmiR-2289-3p;4934;-115;20	*ATP5F1*; BBmiR-1608-5p; 2060; -108; 19
APAF1;BBmiR-2164-3p;5229;-113;20	*ATP5J2*-PT;BBmiR-531-5p;4251;-108;19
APAF1;BBmiR-1189-3p;5230;-113;20	*ATP5J2*-PT;BBmiR-1641-5p;4252;-102;18

* - CDS; ** - 5'UTR.
