# Supplementary material for: Features of Complementary Interactions of BmiRNAs with mRNAs of Human Genes
Source: Int J Mol Sci. 2026 Sep 30;27(19):8773. doi: 10.3390/ijms27198773 (PMC13665148; doi:10.3390/ijms27198773)
Supplement: Supplementary file 1 [file ijms-27-08773-s001.zip › Table S10.pdf]

Table S10. Quantitative Characteristics of BmiRNA Binding to mRNAs of Genes Beginning with "K".

Gene; BmiRNA; BS, nt; ΔG, kJ/mole; BmiRNA length, nt	
KATNAL1; BmiR-1169-3p; 3925; ** -115; 20;	*KIAA1143*;BmiR-1441-3p; 4516; -123; 22;
KATNAL1; BmiR-1297-3p; 3498; -100; 20;	*KIAA1143*;BmiR-1608-5p; 4038; -108; 19;
KATNAL1; BmiR-2131-3p; 4105; -104; 20;	*KIAA1143*;BmiR-1641-5p; 4037; -102; 18;
KATNAL1; BmiR-2289-3p; 3927; -115; 20;	*KIAA1143*;BmiR-2289-3p; 4520; -115; 20;
KAZALD1; BmiR-1993-3p; 1297*; -115; 21;	*KIAA1324*;BmiR-1169-3p; 6305; -115; 20;
KCMF1;BmiR-531-5p; 6422; -108; 19;	*KIAA1456*;BmiR-2087-3p; 5237; -102; 19;
KCMF1;BmiR-1641-5p; 6423; -102; 18;	*KIAA1467*;BmiR-1169-3p; 3260; -115; 20;
KCMF1;BmiR-2087-3p; 5165; -102; 19;	*KIAA1467*;BmiR-2289-3p; 3262; -115; 20;
KCNA7;BmiR-531-5p; 3419; -108; 19;	*KIAA1549*;BmiR-531-5p; 11553; -108; 19;
KCNA7;BmiR-531-5p; 3832; -108; 19;	*KIAA1549*;BmiR-1641-5p; 11554; -102; 18;
KCNA7;BmiR-1608-5p; 3421; -108; 19;	*KIAA1586*;BmiR-1639-5p; 187**; -127; 22;
KCNA7;BmiR-1608-5p; 3834; -108; 19;	*KIAA1609*;BmiR-531-5p; 4441; -108; 19;
KCNA7;BmiR-1641-5p; 3420; -102; 18;	*KIAA1609*;BmiR-1608-5p; 4443; -108; 19;
KCNA7;BmiR-1641-5p; 3833; -102; 18;	*KIAA1609*;BmiR-1641-5p; 4442; -102; 18;
KCNE3;BmiR-531-5p; 2125; -108; 19;	*KIAA2018*;BmiR-1169-3p; 8512; -115; 20;
KCNE3;BmiR-1641-5p; 2126; -102; 18;	*KIAA2018*;BmiR-2289-3p; 8514; -115; 20;
KCNE4;BmiR-1169-3p; 2221; -115; 20;	*KIF18B*;BmiR-1169-3p; 3241; -115; 20;
KCNH6; BmiR-531-5p; 3362; -108; 19;	*KIF18B*;BmiR-1169-3p; 3574; -115; 20;
KCNH6;BmiR-1641-5p; 3363; -102; 18;	*KIF1C*;BmiR-531-5p; 5297; -108; 19;
KCNJ11;BmiR-1169-3p; 102**; -115; 20;	*KIF1C*;BmiR-1641-5p; 5298; -102; 18;
KCNJ11;BmiR-1791-5p; 157**; -102; 19;	*KIF1C*;BmiR-1791-5p; 6741; -102; 19;
KCNJ11;BmiR-2289-3p; 104**; -115; 20;	*KIF3A*;BmiR-531-5p; 3658; -108; 19;
KCNJ3;BmiR-531-5p; 4228; -108; 19;	*KIF3A*;BmiR-1169-3p; 4929; -115; 20;
KCNJ3;BmiR-1641-5p; 4229; -102; 18;	*KIF3A*;BmiR-1641-5p; 3659; -102; 18;
KCNJ5;BmiR-531-5p; 2857; -108; 19;	*KIF3A*;BmiR-2289-3p; 4931; -115; 20;
KCNJ5;BmiR-1641-5p; 2858; -102; 18;	*KLF8*;BmiR-1189-3p; 3724; -113; 20;
KCNMB4;BmiR-2289-3p; 2504; -115; 20;	*KLF8*;BmiR-1441-3p; 3722; -123; 22;
KCNN3;BmiR-1169-3p; 9705; -115; 20;	*KLF8*;BmiR-2289-3p; 3726; -115; 20;
KCNN3;BmiR-2289-3p; 9707; -115; 20;	*KLHL24*;BmiR-1169-3p; 5798; -115; 20;
KCTD20;BmiR-531-5p; 4972; -108; 19;	*KLHL24*;BmiR-2289-3p; 5800; -115; 20;
KCTD20;BmiR-1608-5p; 4974; -108; 19;	*KLHL5*; BmiR-531-5p; 4257; -108; 19;
KCTD20;BmiR-1641-5p; 4973; -102; 18;	*KLHL5*; BmiR-1641-5p; 4258; -102; 18;
KDELC2;BmiR-531-5p; 3302; -108; 19;	*KLHL6*;BmiR-1169-3p; 3068; -115; 20;
KDELC2;BmiR-1641-5p; 3303; -102; 18;	*KLHL6*;BmiR-2289-3p; 3070; -115; 20;
KDM4B;BmiR-531-5p; 5011; -108; 19;	*KLRD1*;BmiR-531-5p; 2084; -108; 19;
KDM4B;BmiR-1608-5p; 5013; -108; 19;	*KLRD1*;BmiR-1641-5p; 2085; -102; 18;
KDM4B;BmiR-1641-5p; 5012; -102; 18;	*KPNA4*;BmiR-1791-5p; 7765; -102; 19;
KIAA0226; BmiR-2087-3p; 5543; -102; 19;	*KPNA4*;BmiR-2131-3p; 7892; -104; 20;
KIAA0355;BmiR-531-5p; 5748; -108; 19;	*KREMEN1*;BmiR-531-5p; 2427; -108; 19;
KIAA0355;BmiR-1641-5p; 5749; -102; 18;	*KREMEN1*;BmiR-531-5p; 3019; -108; 19;
KIAA0408;BmiR-531-5p; 6147; -108; 19;	*KREMEN1*;BmiR-1641-5p; 2428; -102; 18;
KIAA0408;BmiR-1641-5p; 6148; -102; 18;	*KREMEN1*;BmiR-1641-5p; 3020; -102; 18;
KIAA0513;BmiR-1169-3p; 5113; -115; 20;	*KRI1*;BmiR-531-5p; 2456; -108; 19;
KIAA0825;BmiR-1379-5p; 5454; -102; 19;	*KRI1*;BmiR-1608-5p; 2458; -108; 19;
KIAA0825;BmiR-1379-5p; 5578; -102; 19;	*KRI1*;BmiR-1641-5p; 2457; -102; 18;
KIAA1143;BmiR-531-5p; 4036; -108; 19;	*KTN1*;BmiR-1189-3p; 167**; -113; 20;
KIAA1143;BmiR-1189-3p; 4518; -113; 20;	*KTN1*;BmiR-1441-3p; 165**; -123; 22;

* - CDS, ** - 5'UTR.
