# Supplementary material for: Features of Complementary Interactions of BmiRNAs with mRNAs of Human Genes
Source: Int J Mol Sci. 2026 Sep 30;27(19):8773. doi: 10.3390/ijms27198773 (PMC13665148; doi:10.3390/ijms27198773)
Supplement: Supplementary file 1 [file ijms-27-08773-s001.zip › Table S11.pdf]

Table S11. Quantitative Characteristics of BmiRNA Binding to mRNAs of Genes Beginning with "L".

**Gene; BmiRNA; BS, nt; ΔG, kJ/mole; BmiRNA length, nt**	
L2HGDH;BmiR-531-5p; 1749; -108; 19	*LLGL1*;BmiR-2289-3p; 3633; -115; 20
L2HGDH;BmiR-1641-5p; 1750; -102; 18	*LNX2*;BmiR-1189-3p; 2883; -113; 20
LAIR1;BmiR-531-5p; 2651; -108; 19	*LNX2*;BmiR-1441-3p; 2881; -123; 22
LAIR1;BmiR-1641-5p; 2652; -102; 18	*LNX2*;BmiR-2289-3p; 2885; -115; 20
LAMA4;BmiR-1608-5p; 6836; -108; 19	*LOC100996*; BmiR-1189-3p; 192*; -113; 20
LAMP3;BmiR-1927-5p; 2057; -113; 19	*LOC100996*; BmiR-1441-3p; 190*; -123; 22
LARS;BmiR-2087-3p; 4700; -102; 19	*LOC100996*; BmiR-2289-3p; 194*; -115; 20
LDHAL6A;BmiR-1189-3p; 2032; -113; 20	*LPCAT2*;BmiR-1189-3p; 2440; -113; 20
LDHAL6A;BmiR-1441-3p; 2030; -123; 22	*LPCAT2*;BmiR-1441-3p; 2438; -123; 22
LDHAL6A;BmiR-2289-3p; 2034; -115; 20	*LPIN1*;BmiR-1641-5p; 4473; -102; 18
LDLR;BmiR-531-5p; 4610; -108; 19	*LPIN3*;BmiR-1169-3p; 2954; -115; 20
LDLR;BmiR-1608-5p; 4612; -108; 19	*LPIN3*;BmiR-1927-5p; 2994; -113; 19
LDLR;BmiR-1641-5p; 4611; -102; 18	*LPIN3*;BmiR-2289-3p; 2956; -115; 20
LDLR;BmiR-2038-5p; 4611; -102; 18	*LPP*;BmiR-531-5p; 11298; -108; 19
LEPREL4;BmiR-531-5p; 2220; -108; 19	*LPP*;BmiR-1169-3p; 10384; -115; 20
LEPREL4;BmiR-1641-5p; 2221; -102; 18	*LPP*;BmiR-1641-5p; 11299; -102; 18
LEPREL4;BmiR-2087-3p; 1893; -102; 19	*LPP*;BmiR-2289-3p; 10386; -115; 20
LEPROT;BmiR-1169-3p; 4161; -115; 20	*LRAT*;BmiR-531-5p; 3463; -108; 19
LETM1;BmiR-1189-3p; 4598; -113; 20	*LRAT*;BmiR-1608-5p; 3465; -108; 19
LETM1;BmiR-1441-3p; 4596; -123; 22	*LRAT*;BmiR-1641-5p; 3464; -102; 18
LGMN;BmiR-1169-3p; 229**; -115; 20	*LRPAP1*;BmiR-531-5p; 6069; -108; 19
LGMN;BmiR-2289-3p; 231**; -115; 20	*LRPAP1*;BmiR-1608-5p; 6071; -108; 19
LILRB3;BmiR-531-5p; 2735; -108; 19	*LRPAP1*;BmiR-1641-5p; 6070; -102; 18
LILRB3;BmiR-1641-5p; 2736; -102; 18	*LRRC2*;BmiR-1641-5p; 2104; -102; 18
LIMD1;BmiR-1169-3p; 5373; -115; 20	*LRRC2*;BmiR-2087-3p; 2052; -102; 19
LIMD1;BmiR-2087-3p; 5938; -102; 19	*LRRC27*; BmiR-531-5p; 4833; -108; 19
LIMD1;BmiR-2289-3p; 5375; -115; 20	*LRRC274*; BmiR-1641-5p; 4834; -102; 18
LIMS1; BmiR-531-5p; 3706; -108; 19	*LRRC37A3*;BmiR-1791-5p; 86; -102; 19
LIMS1; BmiR-531-5p; 4163; -108; 19	*LRRC47*;BmiR-1791-5p; 1969; -102; 19
LIMS1; BmiR-1608-5p; 3708; -108; 19	*LRRC47*;BmiR-2289-3p; 1916; -115; 20
LIMS1; BmiR-1641-5p; 3707; -102; 18	*LRRFIP1*; BmiR-2087-3p; 3749; -102; 19
LIMS1; BmiR-1641-5p; 4164; -102; 18	*LRRN4CL*;BmiR-531-5p; 2258; -108; 19
LIN28A;BmiR-1189-3p; 2868; -113; 20	*LRRN4CL*;BmiR-1641-5p; 2259; -102; 18
LIN28A;BmiR-1441-3p; 2866; -123; 22	*LRRN4CL*;BmiR-1791-5p; 1783; -102; 19
LIN28A;BmiR-2289-3p; 2870; -115; 20	*LY75*;BmiR-1791-5p; 5849; -102; 19
LIN52;BmiR-531-5p; 1756; -108; 19	*LYRM7*;BmiR-531-5p; 2060; -108; 19
LIN52;BmiR-1608-5p; 1758; -108; 19	*LYRM7*;BmiR-1641-5p; 2061; -102; 18
LIN52;BmiR-1641-5p; 1757; -102; 18	*LYZ*;BmiR-1169-3p; 811; -115; 20
LIN54; BmiR-1791-5p; 3660; -102; 19	*LYZ*;BmiR-2289-3p; 813; -115; 20
LIPT1; BmiR-1791-5p; 353**; -102; 19	*LZTS1*;BmiR-2289-3p; 3328; -115; 20
LLGL1;BmiR-1169-3p; 3631; -115; 20	

* - CDS, ** - 5'UTR.
