# Supplementary material for: Features of Complementary Interactions of BmiRNAs with mRNAs of Human Genes
Source: Int J Mol Sci. 2026 Sep 30;27(19):8773. doi: 10.3390/ijms27198773 (PMC13665148; doi:10.3390/ijms27198773)
Supplement: Supplementary file 1 [file ijms-27-08773-s001.zip › Table S12 .pdf]

Table S12. Quantitative Characteristics of BmiRNA Binding to mRNAs of Genes Beginning with "N".

Gene; BmiRNA; BS, nt; ΔG, kJ/mole; BmiRNA length, nt	
NAA16;miR-531-5p;3565;-108;19	*NMT2*;miR-531-5p;3265;-108;19
NAA16;miR-1608-5p;3567;-108;19	*NMT2*;miR-531-5p;4723;-108;19
NAA16;miR-1641-5p;3566;-102;18	*NMT2*;miR-1608-5p;4725;-108;19
NAA16;miR-2087-3p;3513;-102;19	*NMT2*;miR-1641-5p;2531;-102;18
NACA;miR-2116-5p;2534;CDS;-83;15	*NMT2*;miR-1641-5p;3266;-102;18
NADKD1;miR-1791-5p;3074;-102;19	*NMT2*;miR-1641-5p;;4724;-102;18
NANOG;miR-1169-3p;1400;-115;20	*NMT2*;miR-2087-3p;2479;-102;19
NANOG;miR-2289-3p;1402;-115;20	*NMUR1*;miR-1791-5p;1956;-102;19
NAPEPLD;miR-531-5p;2545;-108;19	*NOL9*;miR-531-5p;3261;-108;19
NAPEPLD;miR-1641-5p;2546;-102;18	*NOL9*;miR-1169-3p;4502;-115;20
NAPG;miR-1169-3p;1979;-115;20	*NOL9*;miR-1608-5p;3263;-108;19
NAPG;miR-2289-3p;1981;-115;20	*NOL9*;miR-1641-5p;3262;-102;18
NARG2;miR-1169-3p;4001;-115;20	*NOL9*;miR-1791-5p;5586;-102;19
NARG2;miR-2289-3p;4003;-115;20	*NOL9*;miR-2289-3p;4504;-115;20
NAV1;miR-1791-5p;11805;-102;19	*NOM1*;miR-1791-5p;3398;-102;19
NCBP1;miR-531-5p;4720;-108;19	*NOM1*;miR-2087-3p;5632;-102;19
NCBP1;miR-1641-5p;4721;-102;18	*NOS1*;miR-531-5p;9412;-108;19
NCBP1;miR-2087-3p;4668;-102;19	*NOS1*;miR-1641-5p;9413;-102;18
NCMAP;miR-531-5p;2486;-108;19	*NOX4*;miR-531-5p;3549;-108;19
NCMAP;miR-1608-5p;2488;-108;19	*NOX4*;miR-1641-5p;3550;-102;18
NCMAP;miR-1641-5p;2487;-102;18	*NPAP1*;miR-1189-3p;6582;-113;20
NCOA7;miR-1169-3p;303;5'UTR;-115;20	*NPAP1*;miR-1441-3p;6580;-123;22
NDRG3;miR-1791-5p;2454;-102;19	*NPAP1*;miR-2289-3p;6584;-115;20
NDUFA4;miR-1189-3p;1063;-113;20	*NPHS1*;miR-531-5p;4392;-108;19
NDUFA4;miR-1441-3p;1061;-123;22	*NPHS1*;miR-1641-5p;4393;-102;18
NDUFAF7;miR-531-5p;1924;-108;19	*NQO1*;miR-1189-3p;1681;-113;20
NDUFAF7;miR-1608-5p;1926;-108;19	*NRAS*;miR-531-5p;3240;-108;19
NDUFAF7;miR-1641-5p;1925;-102;18	*NRAS*;miR-1641-5p;3241;-102;18
NDUFV3;miR-1189-3p;1648;-113;20	*NRIP3*;miR-1169-3p;2939;-115;20
NDUFV3;miR-1441-3p;1646;-123;22	*NRIP3*;miR-2289-3p;2941;-115;20
NDUFV3;miR-2289-3p;1650;-115;20	*NT5DC3*;miR-1169-3p;2984;-115;20
NF2;miR-1169-3p;4345;-115;20	*NT5DC3*;miR-1791-5p;3039;-102;19
NFAT5;miR-1791-5p;9736;-102;19	*NT5DC3*;miR-2289-3p;2986;-115;20
NFYB;miR-531-5p;3094;-108;19	*NUAK2*;miR-1791-5p;2650;-102;19
NFYB;miR-1641-5p;3095;-102;18	*NUDT19*;miR-1169-3p;1285;-115;20
NIPAL3;miR-1641-5p;3984;-102;18	*NUDT19*;miR-2289-3p;1287;-115;20
NKPD1;miR-531-5p;4285;-108;19	*NUDT19*;miR-2289-3p;1605;-115;20
NKPD1;miR-1641-5p;4286;-102;18	*NUDT4*;miR-1791-5p;2269;-102;19
NLN;miR-531-5p;6243;-108;19	*NUDT4*;miR-1927-5p;2252;-113;19
NLN;miR-1641-5p;6244;-102;18	*NUGGC*;miR-1169-3p;2635;-115;20
NLRC3;miR-1169-3p;4252;-115;20	*NUGGC*;miR-2289-3p;2637;-115;20
NLRC3;miR-1189-3p;4654;-113;20	*NUPL1*;miR-531-5p;2789;-108;19
NLRC3;miR-1441-3p;4652;-123;22	*NUPL1*;miR-1608-5p;2791;-108;19
NLRC3;miR-1927-5p;4290;-113;19	*NUPL1*;miR-1641-5p;2790;-102;18
NLRC3;miR-2289-3p;4254;-115;20	*NWD1*;miR-531-5p;6941;-108;19
NLRC3;miR-2289-3p;4656;-115;20	*NWD1*;miR-1641-5p;6942;-102;18
NLRP12;miR-531-5p;3812;-108;19	*NXN*;miR-1169-3p;2243;-115;20
NLRP12;miR-1641-5p;3813;-102;18	*NXN*;miR-2289-3p;2245;-115;20
NMNAT1;miR-531-5p;1597;-108;19	*NXNL2*;miR-1169-3p;655*;-115;20
NMNAT1;miR-1608-5p;1599;-108;19	*NXNL2*;miR-2289-3p;657*;-115;20
NMNAT1;miR-1641-5p;1598;-102;18	*NXPE3*;miR-1169-3p;5165;-115;20
NMNAT1;miR-1927-5p;2408;-113;19	*NXPE3*;miR-2289-3p;5167;-115;20
NMNAT1;miR-2087-3p;1846;-102;19	*NYAP2*;miR-531-5p;4550;-108;19
NMT2;miR-531-5p;2530;-108;19	*NYAP2*;miR-1641-5p;4551;-102;18

* - CDS, ** - 5UTR.
