# Supplementary material for: Features of Complementary Interactions of BmiRNAs with mRNAs of Human Genes
Source: Int J Mol Sci. 2026 Sep 30;27(19):8773. doi: 10.3390/ijms27198773 (PMC13665148; doi:10.3390/ijms27198773)
Supplement: Supplementary file 1 [file ijms-27-08773-s001.zip › Table S13.pdf]

Table S13. Quantitative Characteristics of BmiRNA Binding to mRNAs of Genes Beginning with "O".

**Gene; BmiRNA; BS, nt; ΔG, kJ/mole; BmiRNA length, nt**	
ODZ4-v-1; miR-1379-5p; 11328; -102; 19	*OR7D2*; miR-2289-3p; 2540; -115; 20
OGFOD1; miR-531-5p; 2613; -108; 19	*ORAI2*; miR-531-5p; 2624; -108; 19
OGFOD1; miR-1608-5p; 2615; -108; 19	*ORAI2*; miR-531-5p; 4032; -108; 19
OGFOD1; miR-1641-5p; 2614; -102; 18	*ORAI2*; miR-531-5p; 4104; -108; 19
OGFOD3; miR-1169-3p; 3724; -115; 20	*ORAI2*; miR-531-5p; 5108; -108; 19
OGFOD3; miR-2289-3p; 3726; -115; 20	*ORAI2*; miR-531-5p; 8302; -108; 19
OLA1; miR-531-5p; 3763; -108; 19	*ORAI2*; miR-531-5p; 9089; -108; 19
OLA1; miR-1608-5p; 3765; -108; 19	*ORAI2*; miR-1169-3p; 6961; -115; 20
OLA1; miR-1641-5p; 3764; -102; 18	*ORAI2*; miR-1379-5p; 10393; -102; 19
OLAH; miR-531-5p; 1601; -108; 19	*ORAI2*; miR-1608-5p; 9091; -108; 19
OLAH; miR-1641-5p; 1602; -102; 18	*ORAI2*; miR-1641-5p; 2625; -102; 18
OLFML1; miR-1379-5p; 2223; -102; 19	*ORAI2*; miR-1641-5p; 4033; -102; 18
OLFML2A; miR-1169-3p; 3860; -115; 20	*ORAI2*; miR-1641-5p; 4105; -102; 18
OLFML2A; miR-1791-5p; 3915; -102; 19	*ORAI2*; miR-1641-5p; 5109; -102; 18
OLFML2A; miR-2289-3p; 3862; -115; 20	*ORAI2*; miR-1641-5p; 8303; -102; 18
OPA3; miR-531-5p; 4615; -108; 19	*ORAI2*; miR-1641-5p; 9090; -102; 18
OPA3; miR-1641-5p; 4616; -102; 18	*ORAI2*; miR-2289-3p; 6799; -115; 20
OPRK1; miR-1791-5p; 3494; -102; 19	*ORAI2*; miR-2289-3p; 6963; -115; 20
OPTN; miR-531-5p; 2559; -108; 19	*ORC1*; miR-1169-3p; 2885; -115; 20
OPTN; miR-1641-5p; 2560; -102; 18	*ORC1*; miR-2289-3p; 2887; -115; 20
OR51E2; miR-2289-3p; 1628; -115; 20	*OSBPL2*; miR-1791-5p; 2805; -102; 19
OR7D2; miR-1169-3p; 30**; -115; 20	*OSBPL2*; miR-1927-5p; 2788; -113; 19
OR7D2; miR-1169-3p; 2538; -115; 20	*OSTC*; miR-1169-3p; 521*; -115; 20
OR7D2; miR-1791-5p; 2593; -102; 19	*OSTC*; miR-2289-3p; 523*; -115; 20
OR7D2; miR-1927-5p; 1403; -113; 19	*OTUD3*; miR-1791-5p; 5054; -102; 19

* - CDS, ** - 5UTR
