# Supplementary material for: Features of Complementary Interactions of BmiRNAs with mRNAs of Human Genes
Source: Int J Mol Sci. 2026 Sep 30;27(19):8773. doi: 10.3390/ijms27198773 (PMC13665148; doi:10.3390/ijms27198773)
Supplement: Supplementary file 1 [file ijms-27-08773-s001.zip › Table S14.pdf]

Table S14. Quantitative Characteristics of BmiRNA Binding to mRNAs of Genes Beginning with "P".

**Gene; BmiRNA; BS, nt; ΔG, kJ/mole; BmiRNA length, nt**	
PDDC1; miR-1189-3p; 2506; -113; 20	*PNPO*; miR-1441-3p; 1723; -123; 22
PDDC1; miR-1441-3p; 2504; -123; 22	*PNPT1*; miR-531-5p; 4028; -108; 19
PDDC1; miR-1608-5p; 1919; -108; 19	*PNPT1*; miR-1608-5p; 4030; -108; 19
PDDC1; miR-1641-5p; 1918; -102; 18	*PNPT1*; miR-1641-5p; 4029; -102; 18
PDDC1; miR-1791-5p; 2561; -102; 19	*PNRC1*; miR-531-5p; 2065; -108; 19
PDDC1; miR-2289-3p; 2508; -115; 20	*PNRC1*; miR-1641-5p; 2066; -102; 18
PDE4C; miR-531-5p; 3228; -108; 19	*POFUT1*; miR-2289-3p; 2934; -115; 20
PDE4C; miR-531-5p; 3555; -108; 19	*POLD4*; miR-531-5p; 1679; -108; 19
PDE4C; miR-531-5p; 4164; -108; 19	*POLD4*; miR-1641-5p; 1680; -102; 18
PDE4C; miR-1608-5p; 3230; -108; 19	*POLE3*; miR-1169-3p; 1815; -115; 20
PDE4C; miR-1608-5p; 4166; -108; 19	*PLEKHM3*; miR-531-5p; 7735; -108; 19
PDE4C; miR-1641-5p; 3229; -102; 18	*PLEKHM3*; miR-1641-5p; 7736; -102; 18
PDE4C; miR-1641-5p; 3556; -102; 18	*PLXDC1*; miR-531-5p; 2911; -108; 19
PDE4C; miR-1641-5p; 4165; -102; 18	*PLXDC1*; miR-1641-5p; 2912; -102; 18
PDE6A; miR-531-5p; 4715; -108; 19	*PNMA2*; miR-1189-3p; 3558; -113; 20
PDE6A; miR-1169-3p; 4841; -115; 20	*PNMA2*; miR-1441-3p; 3556; -123; 22
PDE6A; miR-1608-5p; 4717; -108; 19	*PNPO*; miR-1189-3p; 1725; -113; 20
PDE6A; miR-1641-5p; 4716; -102; 18	*PNPO*; miR-1441-3p; 1723; -123; 22
PDE6A; miR-2087-3p; 4663; -102; 19	*PNPT1*; miR-531-5p; 4028; -108; 19
PDE7A; miR-1169-3p; 4990; -115; 20	*PNPT1*; miR-1608-5p; 4030; -108; 19
PDE7B; miR-2087-3p; 4614; -102; 19	*PNPT1*; miR-1641-5p; 4029; -102; 18
PDHA1; miR-1169-3p; 2171; -115; 20	*PNRC1*; miR-531-5p; 2065; -108; 19
PDK3; miR-1189-3p; 5802; -113; 20	*PNRC1*; miR-1641-5p; 2066; -102; 18
PDK3; miR-1441-3p; 5800; -123; 22	*POFUT1*; miR-2289-3p; 2934; -115; 20
PDK3; miR-1927-5p; 5840; -113; 19	*POLD4*; miR-531-5p; 1679; -108; 19
PDLIM2; miR-1169-3p; 2412; -115; 20	*POLD4*; miR-1641-5p; 1680; -102; 18
PDLIM2; miR-1379-5p; 2569; -102; 19	*POLE3*; miR-1169-3p; 1815; -115; 20
PDLIM5; miR-531-5p; 2821; -108; 19	*POLE3*; miR-2289-3p; 1817; -115; 20
PDLIM5; miR-1641-5p; 2822; -102; 18	*POLH*; miR-531-5p; 4128; -108; 19
PDPR; miR-531-5p; 7958; -108; 19	*POLH*; miR-1641-5p; 4129; -102; 18
PDPR; miR-1641-5p; 7959; -102; 18	*POLH*; miR-1791-5p; 6614; -102; 19
PDZK1; miR-1169-3p; 99**; -115; 20	*POLH*; miR-1927-5p; 6597; -113; 19
PECAM1; miR-1169-3p; 2125; -115; 20	*POLH*; miR-2038-5p; 4129; -102; 18
PECAM1; miR-2289-3p; 2127; -115; 20	*POLR2M*; miR-1169-3p; 1940; -115; 20
PER2; miR-1169-3p; 5355; -115; 20	*POLR2M*; miR-2289-3p; 1942; -115; 20
PER2; miR-1791-5p; 5410; -102; 19	*POU2F3*; miR-1169-3p; 2549; -115; 20
PER2; miR-2289-3p; 5357; -115; 20	*POU2F3*; miR-1791-5p; 2604; -102; 19
PEX2; miR-1189-3p; 1856; -113; 20	*POU2F3*; miR-2289-3p; 2551; -115; 20
PEX2; miR-1441-3p; 1854; -123; 22	*POU5F1*; miR-1189-3p; 202**; -113; 20
PEX2; miR-2289-3p; 1858; -115; 20	*POU5F1*; miR-1441-3p; 200**; -123; 22
PEX26; miR-1189-3p; 2967; -113; 20	*POU5F1*; miR-1927-5p; 240**; -113; 19
PEX26; miR-1441-3p; 2965; -123; 22	*POU5F1*; miR-2289-3p; 204**; -115; 20
PEX26; miR-2289-3p; 2969; -115; 20	*PPFIBP1*; miR-1169-3p; 5687; -115; 20
PGAP1; miR-2289-3p; 5097; -115; 20	*PPFIBP1*; miR-2289-3p; 5689; -115; 20
PHACTR4; miR-531-5p; 3797; -108; 19	*PPIL6*; miR-1169-3p; 3155; -115; 20
PHACTR4; miR-531-5p; 4869; -108; 19	*PPIL6*; miR-2289-3p; 3157; -115; 20
PHACTR4; miR-1608-5p; 3333; -108; 19	*PPM1K*; miR-1189-3p; 5134; -113; 20
PHACTR4; miR-1608-5p; 4871; -108; 19	*PPM1K*; miR-1441-3p; 5132; -123; 22
PHACTR4; miR-1641-5p; 3332; -102; 18	*PPM1K*; miR-2289-3p; 5136; -115; 20
PHACTR4; miR-1641-5p; 3798; -102; 18	*PPM1K*; miR-2289-3p; 5595; -115; 20
PHACTR4; miR-1641-5p; 4870; -102; 18	*PPP1R12B*; miR-531-5p; 5383; -108; 19
PHACTR4; miR-1927-5p; 5055; -113; 19	*PPP1R12B*; miR-1169-3p; 7575; -115; 20
PHAX; miR-531-5p; 3259; -108; 19	*PPP1R12B*; miR-1641-5p; 5384; -102; 18
PHAX; miR-1641-5p; 3260; -102; 18	*PPP1R14D*; miR-1189-3p; 338*; -113; 20

PHKG2; miR-1169-3p; 2929; -115; 20	*PPP1R14D*; miR-1441-3p; 336; -123; 22
PHKG2; miR-2289-3p; 2447; -115; 20	*PRAMEF1*; miR-2451-3p; 1729; -102; 18
PHKG2; miR-2289-3p; 2931; -115; 20	*PRAMEF13*; miR-2451-3p; 1703; -102; 18
PHLDA1; miR-1189-3p; 3620; -113; 20	*PRAMEF13*; miR-2451-3p; 1731; -102; 18
PHLDA1; miR-2289-3p; 3622; -115; 20	*PRKCA*; miR-1791-5p; 6831; -102; 19
PHYHD1; miR-1791-5p; 312**; -102; 19	*PRKX*; miR-531-5p; 3990; -108; 19
PIGR; miR-1169-3p; 3272; -115; 20	*PRKX*; miR-1641-5p; 3991; -102; 18
PIGR; miR-2289-3p; 3274; -115; 20	*PRLR*; miR-531-5p; 7625; -108; 19
PIGX; miR-531-5p; 2342; -108; 19	*PRLR*; miR-1641-5p; 7626; -102; 18
PIGX; miR-1641-5p; 2343; -102; 18	*PRND*; miR-1169-3p; 3345; -115; 20
PIK3CD; miR-1169-3p; 4141; -115; 20	*PRND*; miR-2289-3p; 3347; -115; 20
PIK3R2; miR-531-5p; 3572; -108; 19	*PRR11*; miR-1441-3p; 3026; -123; 22
PIK3R2; miR-1355-3p; 783*; -136; 22	*PRR11*; miR-2289-3p; 3030; -115; 20
PIK3R2; miR-1641-5p; 3573; -102; 18	*PRRG4*; miR-1189-3p; 4287; -113; 20
PLA2G12A; miR-1361-5p; 2668; -125; 22	*PRRG4*; miR-1189-3p; 4606; -113; 20
PLA2G2D; miR-531-5p; 2544; -108; 19	*PRRG4*; miR-1441-3p; 4604; -123; 22
PLA2G2D; miR-1608-5p; 2546; -108; 19	*PSMB2*; miR-1927-5p; 2013; -113; 19
PLA2G2D; miR-1641-5p; 2545; -102; 18	*PSMD9*; miR-1189-3p; 1520; -113; 20
PLAC8; miR-1169-3p; 984; -115; 20	*PSMD9*; miR-1441-3p; 1518; -123; 22
PLAC8; miR-2289-3p; 986; -115; 20	*PSPH*; miR-1169-3p; 1662; -115; 20
PLAGL1; miR-2485-3p; 143**; -100; 17	*PSPH*; miR-2289-3p; 1664; -115; 20
PLAGL1; miR-2485-5p; 169**; -106; 18	*PTAFR*; miR-2087-3p; 2040; -102; 19
PLCXD1; miR-531-5p; 1961; -108; 19	*PTCD1*; miR-531-5p; 4218; -108; 19
PLCXD1; miR-1169-3p; 3614; -115; 20	*PTCD1*; miR-1641-5p; 4219; -102; 18
PLCXD1; miR-1641-5p; 1962; -102; 18	*PTCD3*; miR-531-5p; 4031; -108; 19
PLCXD1; miR-2289-3p; 3616; -115; 20	*PTCD3*; miR-1608-5p; 4033; -108; 19
PLD6; miR-1169-3p; 1608; -115; 20	*PTCD3*; miR-1641-5p; 4032; -102; 18
PLD6; miR-2289-3p; 1610; -115; 20	*PTGES3L*; miR-1169-3p; 1471; -115; 20
PLEKHA2; miR-531-5p; 3636; -108; 19	*PTK6*; miR-531-5p; 2467; -108; 19
PLEKHA2; miR-1189-3p; 4707; -113; 20	*PTK6*; miR-1641-5p; 2468; -102; 18
PLEKHA2; miR-1441-3p; 4705; -123; 22	*PTPN14*; miR-531-5p; 11540; -108; 19
PLEKHA2; miR-1641-5p; 3637; -102; 18	*PTPN14*; miR-1608-5p; 11542; -108; 19
PLEKHA2; miR-2289-3p; 4709; -115; 20	*PTPN14*; miR-1641-5p; 11541; -102; 18
PLEKHA5; miR-1189-3p; 3966; -113; 20	*PTPRB*; miR-1169-3p; 9774; -115; 20
PLEKHA5; miR-1441-3p; 3964; -123; 22	*PTPRB*; miR-2289-3p; 9776; -115; 20
PLEKHA5; miR-2087-3p; 6211; -102; 19	*PTRF*; miR-531-5p; 2398; -108; 19
PLEKHG4B; miR-531-5p; 5239; -108; 19	*PTRF*; miR-1641-5p; 2399; -102; 18
PLEKHG4B; miR-1641-5p; 5240; -102; 18	*PVR*; miR-1641-5p; 5336; -102; 18
PLEKHH2; miR-1169-3p; 5607; -115; 20	*PXMP4*; miR-531-5p; 1513; -108; 19
PLEKHM3; miR-531-5p; 7735; -108; 19	*PXMP4*; miR-1169-3p; 2885; -115; 20
PLEKHM3; miR-1641-5p; 7736; -102; 18	*PXMP4*; miR-1641-5p; 1514; -102; 18
PLXDC1; miR-531-5p; 2911; -108; 19	*PXMP4*; miR-1641-5p; 2485; -102; 18
PLXDC1; miR-1641-5p; 2912; -102; 18	*PXMP4*; miR-2087-3p; 2432; -102; 19
PNMA2; miR-1189-3p; 3558; -113; 20	*PXMP4*; miR-2289-3p; 2887; -115; 20
PNMA2; miR-1441-3p; 3556; -123; 22	*PYCRL*; miR-531-5p; 2638; -108; 19
PNPO; miR-1189-3p; 1725; -113; 20	*PYCRL*; miR-1641-5p; 2639; -102; 18

* - CDS, ** - 5'UTR;
