# Supplementary material for: Features of Complementary Interactions of BmiRNAs with mRNAs of Human Genes
Source: Int J Mol Sci. 2026 Sep 30;27(19):8773. doi: 10.3390/ijms27198773 (PMC13665148; doi:10.3390/ijms27198773)
Supplement: Supplementary file 1 [file ijms-27-08773-s001.zip › Table S15.pdf]

Table S15. Quantitative Characteristics of BmiRNA Binding to mRNAs of Genes Beginning with "Q".

Gene; BmiRNA; BS, nt; ΔG, kJ/mole; BmiRNA length, nt	
QPCTL; miR-531-5p; 2233; -108; 19	*QRFPR*; miR-531-5p; 2175; -108; 19
QPCTL; miR-1169-3p; 1691; -115; 20	*QRFPR*; miR-1641-5p; 2176; -102; 18
QPCTL; miR-1608-5p; 2235; -108; 19	*QRSL1*; miR-531-5p; 1977; -108; 19
QPCTL; miR-1641-5p; 2234; -102; 18	*QRSL1*; miR-1641-5p; 1978; -102; 18
QPRT; miR-531-5p; 1504; -108; 19	*QTRTD1*; miR-1169-3p; 3021; -115; 20
QPRT; miR-1641-5p; 1505; -102; 18	*QTRTD1*; miR-2289-3p; 3023; -115; 20
