# Supplementary material for: Features of Complementary Interactions of BmiRNAs with mRNAs of Human Genes
Source: Int J Mol Sci. 2026 Sep 30;27(19):8773. doi: 10.3390/ijms27198773 (PMC13665148; doi:10.3390/ijms27198773)
Supplement: Supplementary file 1 [file ijms-27-08773-s001.zip › Table S16.pdf]

**Table S16.** Quantitative Characteristics of BmiRNA Binding to mRNAs of Genes Beginning with "R".

| **Gene; BmiRNA; BS, nt; ΔG, kJ/mole; BmiRNA length, nt** | |
|---|---|
| *RAB11FIP1*; miR-531-5p; 5156; -108; 19 | *RGS12*; miR-1189-3p; 296**; -113; 20 |
| *RAB11FIP1*; miR-1608-5p; 5158; -108; 19 | *RGS12*; miR-1441-3p; 294**; -123; 22 |
| *RAB11FIP1*; miR-1641-5p; 5157; -102; 18 | *RGS12*; miR-2289-3p; 298**; -115; 20 |
| *RAB27A*; miR-531-5p; 2942; -108; 19 | *RHBDL2*; miR-531-5p; 1635; -108; 19 |
| *RAB27A*; miR-1641-5p; 2943; -102; 18 | *RHBDL2*; miR-1641-5p; 1636; -102; 18 |
| *RAB2B*; miR-2289-3p; 1387; -115; 20 | *RHBG*; miR-1791-5p; 323*; -102; 19 |
| *RAB33B*; miR-2289-3p; 1899; -115; 20 | *RHNO1*; miR-1169-3p; 1114; -115; 20 |
| *RAB39A*; miR-1189-3p; 1404; -113; 20 | *RHNO1*; miR-1791-5p; 1169; -102; 19 |
| *RAB39A*; miR-1441-3p; 1402; -123; 22 | *RHOBTB3*; miR-531-5p; 4260; -108; 19 |
| *RAB3D*; miR-1189-3p; 2085; -113; 20 | *RHOBTB3*; miR-1641-5p; 4261; -102; 18 |
| *RAB3D*; miR-1441-3p; 2083; -123; 22 | *RHPN2*; miR-1169-3p; 2996; -115; 20 |
| *RAB3D*; miR-1791-5p; 2805; -102; 19 | *RIMKLA*; miR-531-5p; 4235; -108; 19 |
| *RAB3D*; miR-1791-5p; 3284; -102; 19 | *RIMKLA*; miR-1641-5p; 4236; -102; 18 |
| *RAB42*; miR-531-5p; 1091; -108; 19 | *RING1*; miR-2000-3p; 777*; -123; 21 |
| *RAB42*; miR-1608-5p; 1093; -108; 19 | *RING1*; miR-2000-5p; 817*; -115; 20 |
| *RAB42*; miR-1641-5p; 1092; -102; 18 | *RLIM*; miR-1189-3p; 6541; -113; 20 |
| *RAB7L1*; miR-2087-3p; 2764; -102; 19 | *RLIM*; miR-1441-3p; 6539; -123; 22 |
| *RABL2A*; miR-2289-3p; 1454; -115; 20 | *RLIM*; miR-2289-3p; 6543; -115; 20 |
| *RABL2B*; miR-2289-3p; 1648; -115; 20 | *RNF125*; miR-1169-3p; 3871; -115; 20 |
| *RAD54B*; miR-1791-5p; 2858; -102; 19 | *RNF125*; miR-1927-5p; 3909; -113; 19 |
| *RASAL1*; miR-1246-5p; 3647; -117; 21 | *RNF135*; miR-1169-3p; 826*; -115; 20 |
| *RBBP4*; miR-531-5p; 4462; -108; 19 | *RNF135*; miR-2289-3p; 828*; -115; 20 |
| *RBBP4*; miR-1641-5p; 4463; -102; 18 | *RNF141*; miR-531-5p; 2024; -108; 19 |
| *RBBP4*; miR-1791-5p; 5459; -102; 19 | *RNF141*; miR-1641-5p; 2025; -102; 18 |
| *RBBP5*; miR-531-5p; 4199; -108; 19 | *RNF144A*; miR-1791-5p; 4523; -102; 19 |
| *RBBP5*; miR-1641-5p; 4200; -102; 18 | *RNF157*; miR-531-5p; 3515; -108; 19 |
| *RBL1*; miR-531-5p; 3759; -108; 19 | *RNF157*; miR-1641-5p; 3516; -102; 18 |
| *RBL1*; miR-1608-5p; 3761; -108; 19 | *RNF170*; miR-531-5p; 2125; -108; 19 |
| *RBL1*; miR-1641-5p; 3760; -102; 18 | *RNF170*; miR-1608-5p; 2127; -108; 19 |
| *RBM3*; miR-2289-3p; 3705; -115; 20 | *RNF170*; miR-1641-5p; 2126; -102; 18 |
| *RBM34*; miR-2289-3p; 1552; -115; 20 | *RNF207*; miR-1791-5p; 2259; -102; 19 |
| *RBM4*; miR-1169-3p; 1655; -115; 20 | *RNF213*; miR-1131-5p; 18498; -104; 19 |
| *RBM4*; miR-2289-3p; 1657; -115; 20 | *RNF222*; miR-531-5p; 2687; -108; 19 |
| *RBM41*; miR-531-5p; 3016; -108; 19 | *RNF222*; miR-1641-5p; 2688; -102; 18 |
| *RBM41*; miR-1608-5p; 3018; -108; 19 | *RNF41*; miR-531-5p; 4216; -108; 19 |
| *RBM41*; miR-1641-5p; 3017; -102; 18 | *RNF41*; miR-1608-5p; 4218; -108; 19 |
| *RBM43*; miR-1641-5p; 2693; -102; 18 | *RNF41*; miR-1641-5p; 4217; -102; 18 |
| *RBM43*; miR-2289-3p; 1553; -115; 20 | *RNF7*; miR-1189-3p; 1214; -113; 20 |
| *RBM48*; miR-1791-5p; 1385; -102; 19 | *RNF7*; miR-1927-5p; 1252; -113; 19 |
| *RBMS2*; miR-1169-3p; 5470; -115; 20 | *RNF7*; miR-2289-3p; 1216; -115; 20 |
| *RBMS2*; miR-1169-3p; 5876; -115; 20 | *ROCK2*; miR-531-5p; 6361; -108; 19 |
| *RBMS2*; miR-1189-3p; 5136; -113; 20 | *ROCK2*; miR-1608-5p; 6363; -108; 19 |
| *RBMS2*; miR-1441-3p; 5134; -123; 22 | *ROCK2*; miR-1641-5p; 6362; -102; 18 |
| *RBMS2*; miR-2289-3p; 5472; -115; 20 | *RP2*; miR-1791-5p; 3042; -102; 19 |
| *RBMS2*; miR-2289-3p; 5878; -115; 20 | *RPL15*; miR-1791-5p; 1625; -102; 19 |
| *RC3H1*; miR-1169-3p; 5718; -115; 20 | *RPL15*; miR-1791-5p; 2458; -102; 19 |
| *RC3H1*; miR-1791-5p; 5773; -102; 19 | *RPL22L1*; miR-1189-3p; 759; -113; 20 |
| *REEP3*; miR-2289-3p; 2830; -115; 20 | *RPL22L1*; miR-1441-3p; 757; -123; 22 |
| *RELL1*; miR-1169-3p; 2097; -115; 20 | *RPL27A*; miR-1169-3p; 2675; -115; 20 |
| *RELL1*; miR-1791-5p; 1693; -102; 19 | *RPL27A*; miR-2289-3p; 1280; -115; 20 |
| *RELL1*; miR-2289-3p; 2099; -115; 20 | *RPL28*; miR-1791-5p; 2617; -102; 19 |
| *RFFL*; miR-1169-3p; 2503; -115; 20 | *RPP30*; miR-1189-3p; 846*; -113; 20 |
| *RFT1*; miR-1169-3p; 4771; -115; 20 | |
| * - CDS, ** - 5'UTR. | |
