# Supplementary material for: Features of Complementary Interactions of BmiRNAs with mRNAs of Human Genes
Source: Int J Mol Sci. 2026 Sep 30;27(19):8773. doi: 10.3390/ijms27198773 (PMC13665148; doi:10.3390/ijms27198773)
Supplement: Supplementary file 1 [file ijms-27-08773-s001.zip › Table S17.pdf]

Table S17. Quantitative Characteristics of BmiRNA Binding to mRNAs of Genes Beginning with "S".

Gene; BmiRNA; BS, nt; ΔG, kJ/mole; BmiRNA length, nt	
S1PR2; miR-1169-3p; 1553; -115; 20	*SMAD5*; miR-531-5p; 3373; -108; 19
S1PR2; miR-1791-5p; 2839; -102; 19	*SMAD5*; miR-1641-5p; 3374; -102; 18
S1PR2; miR-2289-3p; 1555; -115; 20	*SMARCA5*; miR-1189-3p; 6126; -113; 20
SAR1B; miR-1169-3p; 4944; -115; 20	*SMARCA5*; miR-1441-3p; 6124; -123; 22
SAR1B; miR-1189-3p; 5778; -113; 20	*SMC5*; miR-1169-3p; 5357; -115; 20
SAR1B; miR-1441-3p; 5776; -123; 22	*SMC5*; miR-1791-5p; 5412; -102; 19
SBNO1; miR-531-5p; 9294; -108; 19	*SMC5*; miR-1927-5p; 5395; -113; 19
SBNO1; miR-1608-5p; 9296; -108; 19	*SMC5*; miR-2289-3p; 5359; -115; 20
SBNO1; miR-1641-5p; 9295; -102; 18	*SMCR7L*; miR-531-5p; 4229; -108; 19
SBSPON; miR-1169-3p; 2584; -115; 20	*SMCR7L*; miR-1641-5p; 4230; -102; 18
SBSPON; miR-2289-3p; 2586; -115; 20	*SMG1*; miR-531-5p; 13100; -108; 19
SC5DL; miR-2289-3p; 4108; -115; 20	*SMG1*; miR-1608-5p; 13102; -108; 19
SCAMP1; miR-531-5p; 5510; -108; 19	*SMG1*; miR-1641-5p; 13101; -102; 18
SCAMP1; miR-1641-5p; 5511; -102; 18	*SMPDL3B*; miR-531-5p; 1725; -108; 19
SCAMP4; miR-1169-3p; 1650; -115; 20	*SMPDL3B*; miR-1641-5p; 1726; -102; 18
SCD5; miR-1189-3p; 1551; -113; 20	*SMU1*; miR-531-5p; 6049; -108; 19
SCD5; miR-1441-3p; 1549; -123; 22	*SMU1*; miR-1169-3p; 3120; -115; 20
SCD5; miR-2289-3p; 1553; -115; 20	*SMU1*; miR-1641-5p; 6050; -102; 18
SCD5; miR-2289-3p; 1858; -115; 20	*SMU1*; miR-1791-5p; 3175; -102; 19
SCNM1; miR-1169-3p; 1072; -115; 20	*SMUG1*; miR-1169-3p; 2093; -115; 20
SCNM1; miR-2289-3p; 1074; -115; 20	*SMUG1*; miR-2289-3p; 2095; -115; 20
SDE2; miR-1791-5p; 2541; -102; 19	*SMYD4*; miR-531-5p; 3189; -108; 19
SDE2; miR-2289-3p; 1925; -115; 20	*SMYD4*; miR-1641-5p; 3190; -102; 18
SDSL; miR-1189-3p; 86**; -113; 20	*SMYD4*; miR-1641-5p; 3594; -102; 18
SDSL; miR-1441-3p; 84**; -123; 22	*SNAPC1*; miR-531-5p; 2506; -108; 19
SDSL; miR-2289-3p; 88**; -115; 20	*SNAPC1*; miR-1608-5p; 2508; -108; 19
SEC14L4; miR-531-5p; 2130; -108; 19	*SNAPC1*; miR-1641-5p; 2507; -102; 18
SEC14L4; miR-1608-5p; 2132; -108; 19	*SNIP1*; miR-531-5p; 3187; -108; 19
SEC14L4; miR-1641-5p; 2131; -102; 18	*SNIP1*; miR-1169-3p; 3684; -115; 20
SEC62; miR-1189-3p; 5673; -113; 20	*SNIP1*; miR-1641-5p; 3188; -102; 18
SEC62; miR-1441-3p; 5671; -123; 22	*SNIP1*; miR-2289-3p; 3686; -115; 20
SEC62; miR-2289-3p; 5675; -115; 20	*SNTB2*; miR-2289-3p; 3640; -115; 20
SEMA4F; miR-531-5p; 4628; -108; 19	*SNX11*; miR-1558-3p; 1755; -127; 23
SEMA4F; miR-1608-5p; 4630; -108; 19	*SNX22*; miR-531-5p; 1455; -108; 19
SEMA4F; miR-1641-5p; 4629; -102; 18	*SPOP*; miR-1189-3p; 250**; -113; 20
SENP5; miR-1169-3p; 5234; -115; 20	*SPOP*; miR-1441-3p; 248**; -123; 22
SENP5; miR-1791-5p; 4704; -102; 19	*SPOP*; miR-2289-3p; 252**; -115; 20
SEPT14; miR-531-5p; 2620; -108; 19	*SPRED1*; miR-1791-5p; 5056; -102; 19
SEPT14; miR-1641-5p; 2621; -102; 18	*SPRYD4*; miR-1189-3p; 1486; -113; 20
SERAC1; miR-1521-5p; 3157; -108; 20	*SPRYD4*; miR-1441-3p; 1484; -123; 22
SERBP1; miR-1189-3p; 5368; -113; 20	*SPRYD4*; miR-2289-3p; 1488; -115; 20
SERBP1; miR-1441-3p; 5366; -123; 22	*SRFBP1*; miR-531-5p; 2802; -108; 19
SERBP1; miR-2289-3p; 5370; -115; 20	*SRFBP1*; miR-1641-5p; 2803; -102; 18
SERF1A; miR-1927-5p; 967; -113; 19	*SRL*; miR-1791-5p; 2054; -102; 19
SERF1B; miR-1927-5p; 961; -113; 19	*SRSF10*; miR-531-5p; 1720; -108; 19
SERPINB1; miR-1189-3p; 2209; -113; 20	*SRSF10-*; miR-1608-5p; 1722; -108; 19
SERPINB1; miR-2289-3p; 2211; -115; 20	*SRSF10*; miR-1641-5p; 1721; -102; 18
SERPINB9; miR-1169-3p; 1743; -115; 20	*SS18*; miR-1169-3p; 2988; -115; 20
SERPINB9; miR-2289-3p; 1745; -115; 20	*SS18*; miR-2289-3p; 2990; -115; 20
SFTPB; miR-531-5p; 2422; -108; 19	*SSH1*; miR-531-5p; 6046; -108; 19
SFTPB; miR-1641-5p; 2423; -102; 18	*SSH1*; miR-1641-5p; 6047; -102; 18
SFXN1; miR-1189-3p; 2332; -113; 20	*SSH1*; miR-2087-3p; 5994; -102; 19
SFXN1; miR-1441-3p; 2330; -123; 22	*SSR1*; miR-531-5p; 7608; -108; 19
SFXN1; miR-2289-3p; 2334; -115; 20	*SSR1*; miR-531-5p; 7775; -108; 19

SGCB; miR-1791-5p; 3394; -102; 19	*SSR1*; miR-1189-3p; 4023; -113; 20
SGCB; miR-2289-3p; 3334; -115; 20	*SSR1*; miR-1441-3p; 4021; -123; 22
SGK494; miR-1169-3p; 1712; -115; 20	*SSR1*; miR-1641-5p; 7609; -102; 18
SGOL1; miR-531-5p; 3879; -108; 19	*SSR1*; miR-1641-5p; 7776; -102; 18
SGOL1; miR-1641-5p; 3880; -102; 18	*ST3GAL1*; miR-531-5p; 2875; -108; 19
SGOL1; miR-1791-5p; 2259; -102; 19	*ST3GAL1*; miR-1608-5p; 2877; -108; 19
SGTB; miR-531-5p; 3370; -108; 19	*ST3GAL1*; miR-1641-5p; 2876; -102; 18
SGTB; miR-1641-5p; 3371; -102; 18	*ST3GAL2*; miR-531-5p; 4205; -108; 19
SGTB; miR-2289-3p; 2664; -115; 20	*ST3GAL2*; miR-1641-5p; 4206; -102; 18
SH3GLB1; miR-1131-5p; 5084; -104; 19	*ST8SIA1*; miR-531-5p; 3891; -108; 19
SH3TC2; miR-1169-3p; 12492; -115; 20	*ST8SIA1*; miR-1641-5p; 3892; -102; 18
SH3TC2; miR-1246-5p; 12478; -117; 21	*STAT2*; miR-1246-5p; 3566; -117; 21
SHISA2; miR-1169-3p; 1428; -115; 20	*STAT3*; miR-2087-3p; 3302; -102; 19
SHOX; miR-1169-3p; 2870; -115; 20	*STEAP2*; miR-1189-3p; 3945; -113; 20
SHOX; miR-2289-3p; 2872; -115; 20	*STEAP2*; miR-1441-3p; 3943; -123; 22
SHROOM1; miR-531-5p; 3738; -108; 19	*STEAP2*; miR-2289-3p; 3947; -115; 20
SHROOM1; miR-1641-5p; 3739; -102; 18	*STK4*; miR-1169-3p; 4623; -115; 20
SHROOM1; miR-2289-3p; 321*; -115; 20	*STK4*; miR-1927-5p; 3859; -113; 19
SIGLEC11; miR-531-5p; 3109; -108; 19	*STK4*; miR-2289-3p; 4625; -115; 20
SIGLEC11; miR-1608-5p; 3111; -108; 19	*SNX22*; miR-1608-5p; 1457; -108; 19
SIGLEC11; miR-1641-5p; 3110; -102; 18	*SNX22*; miR-1641-5p; 1456; -102; 18
SIGLEC8; miR-1169-3p; 1910; -115; 20	*SNX27*; miR-1379-5p; 3618; -102; 19
SIKE1; miR-1791-5p; 4667; -102; 19	*SNX27*; miR-1791-5p; 4746; -102; 19
SIRT5; miR-1169-3p; 1760; -115; 20	*SOAT1*; miR-1246-5p; 4965; -117; 21
SIRT5; miR-1927-5p; 1798; -113; 19	*SOAT1*; miR-1791-5p; 5540; -102; 19
SIX4; miR-1169-3p; 4050; -115; 20	*SOGA3*; miR-531-5p; 9841; -108; 19
SIX4; miR-2289-3p; 4052; -115; 20	*SOGA3*; miR-1641-5p; 9842; -102; 18
SKAP2; miR-531-5p; 2315; -108; 19	*SOWAHC*; miR-531-5p; 3646; -108; 19
SKAP2; miR-1641-5p; 2316; -102; 18	*SOWAHC*; miR-1608-5p; 3648; -108; 19
SKIDA1; miR-1169-3p; 5883; -115; 20	*SOWAHC*; miR-1641-5p; 3647; -102; 18
SKIDA1; miR-2289-3p; 5885; -115; 20	*SP4*; miR-1791-5p; 3923; -102; 19
SLC12A6; miR-531-5p; 6102; -108; 19	*SPAG6*; miR-1189-3p; 428*; -113; 20
SLC12A6; miR-1641-5p; 6103; -102; 18	*SPAG6*; miR-1441-3p; 426*; -123; 22
SLC12A6; miR-1791-5p; 6465; -102; 19	*SPAG6*; miR-1791-5p; 357*; -102; 19
SLC13A1; miR-1169-3p; 3269; -115; 20	*SPAG6*; miR-2289-3p; 430*; -115; 20
SLC13A1; miR-2289-3p; 3271; -115; 20	*SPAG9*; miR-531-5p; 5594; -108; 19
SLC14A2; miR-531-5p; 4113; -108; 19	*SPAG9*; miR-1641-5p; 5595; -102; 18
SLC14A2; miR-1608-5p; 4115; -108; 19	*SPAG9*; miR-2131-3p; 6021; -104; 20
SLC14A2; miR-1641-5p; 4114; -102; 18	*SPATA13*; miR-531-5p; 5248; -108; 19
SLC16A4; miR-1169-3p; 1876; -115; 20	*SPATA13*; miR-1641-5p; 5249; -102; 18
SLC16A4; miR-2289-3p; 1878; -115; 20	*SPATA5*; miR-531-5p; 5875; -108; 19
SLC16A4; miR-531-5p; 2616; -108; 19	*SPATA5*; miR-1641-5p; 5876; -102; 18
SLC16A4; miR-1641-5p; 2617; -102; 18	*SPATS2*; miR-531-5p; 3558; -108; 19
SLC22A10; n-miR-137-5; 12**; -76; 15	*SPATS2*; miR-1641-5p; 3559; -102; 18
SLC24A2; miR-1521-5p; 9704; -108; 20	*SPATS2L*; miR-1169-3p; 5778; -115; 20
SLC24A4; miR-1169-3p; 7841; -115; 20	*SPATS2L*; miR-2289-3p; 5780; -115; 20
SLC24A4; miR-1169-3p; 8159; -115; 20	*SPCS3*; miR-1791-5p; 4034; -102; 19
SLC24A4; miR-1189-3p; 5127; -113; 20	*SPIB*; miR-531-5p; 2145; -108; 19
SLC24A4; miR-1441-3p; 5125; -123; 22	*SPIB*; miR-531-5p; 2439; -108; 19
SLC24A4; miR-2289-3p; 7843; -115; 20	*SPIB*; miR-1641-5p; 2146; -102; 18
SLC24A4; miR-2289-3p; 8161; -115; 20	*SPIB*; miR-1641-5p; 2440; -102; 18
SLC25A32; miR-1169-3p; 1779; -115; 20	*SPIB*; miR-2087-3p; 3127; -102; 19
SLC25A32; miR-2289-3p; 1781; -115; 20	*SPIRE2*; miR-531-5p; 2970; -108; 19
SLC26A4; miR-531-5p; 4446; -108; 19	*SPIRE2*; miR-1641-5p; 2971; -102; 18
SLC26A4; miR-1641-5p; 4447; -102; 18	*SPN*; miR-531-5p; 6565; -108; 19
SLC28A2; miR-531-5p; 2423; -108; 19	*SPN*; miR-1608-5p; 6567; -108; 19
SLC28A2; miR-1641-5p; 2424; -102; 18	*SPN*; miR-1641-5p; 6566; -102; 18

SLC30A4; miR-1189-3p; 2918; -113; 20	*STON2*; miR-1927-5p; 10114; -113; 19
SLC30A4; miR-1441-3p; 2916; -123; 22	*STX11*; miR-1169-3p; 2331; -115; 20
SLC30A4; miR-2289-3p; 2920; -115; 20	*STX11*; miR-2289-3p; 2333; -115; 20
SLC30A7; miR-2289-3p; 2028; -115; 20	*STX16*; miR-531-5p; 4040; -108; 19
SLC35E2; miR-1169-3p; 1797; -115; 20	*STX16*; miR-1641-5p; 4041; -102; 18
SLC35E2; miR-2289-3p; 1799; -115; 20	*STX1B*; miR-1189-3p; 1305; -113; 20
SLC35E3; miR-531-5p; 1820; -108; 19	*STX1B*; miR-1441-3p; 1303; -123; 22
SLC35E3; miR-1641-5p; 1821; -102; 18	*STX1B*; miR-2289-3p; 1307; -115; 20
SLC35E4; miR-1641-5p; 2027; -102; 18	*STX3*; miR-531-5p; 5729; -108; 19
SLC36A2; miR-1791-5p; 1736; -102; 19	*STX3*; miR-1608-5p; 5731; -108; 19
SLC36A3; miR-1189-3p; 842*; -113; 20	*STX3*; miR-1641-5p; 5730; -102; 18
SLC36A3; miR-1441-3p; 840*; -123; 22	*SULT2A1*; miR-531-5p; 1551; -108; 19
SLC36A3; miR-2289-3p; 844*; -115; 20	*SULT2A1*; miR-1641-5p; 1552; -102; 18
SLC44A4-v-3; miR-1131-5p; 2546; -104; 19	*SUPT7L*; miR-1791-5p; 3002; -102; 19
SLC4A1; miR-531-5p; 4425; -108; 19	*SUSD5*; miR-1169-3p; 2652; -115; 20
SLC4A1; miR-1608-5p; 4427; -108; 19	*SWSAP1*; miR-531-5p; 1621; -108; 19
SLC4A1; miR-1641-5p; 4426; -102; 18	*SWSAP1*; miR-1641-5p; 1622; -102; 18
SLC4A8; miR-531-5p; 6405; -108; 19	*SYAP1*; miR-531-5p; 2011; -108; 19
SLC4A8; miR-1169-3p; 7451; -115; 20	*SYAP1*; miR-531-5p; 2473; -108; 19
SLC4A8; miR-1641-5p; 6406; -102; 18	*SYAP1*; miR-1641-5p; 2012; -102; 18
SLC5A5; miR-1189-3p; 2589; -113; 20	*SYAP1*; miR-1641-5p; 2474; -102; 18
SLC5A5; miR-1441-3p; 2587; -123; 22	*SYNJ2BP*; miR-531-5p; 2085; -108; 19
SLC7A14; miR-1189-3p; 6978; -113; 20	*SYNJ2BP*; miR-1641-5p; 2086; -102; 18
SLC7A14; miR-1441-3p; 6976; -123; 22	*SYNJ2BP*; miR-1791-5p; 6071; -102; 19
SLC7A14; miR-2289-3p; 6980; -115; 20	*SYNPO2L*; miR-531-5p; 3921; -108; 19
SLC7A6; miR-1791-5p; 232**; -102; 19	*SYNPO2L*; miR-1641-5p; 3922; -102; 18
SLC9A7; miR-1189-3p; 3143; -113; 20	*SYNRG*; miR-531-5p; 7451; -108; 19
SLC9A7; miR-1189-3p; 4107; -113; 20	*SYNRG*; miR-1608-5p; 7453; -108; 19
SLC9A7; miR-1441-3p; 3141; -123; 22	*SYNRG*; miR-1641-5p; 7452; -102; 18
SLC9A7; miR-1441-3p; 4105; -123; 22	*SYNRG*; miR-1791-5p; 5831; -102; 19
SLC9A7; miR-1927-5p; 4145; -113; 19	*SYNRG*; miR-1927-5p; 5814; -113; 19
SLC9A7; miR-2289-3p; 3145; -115; 20	*SYS1*; miR-1169-3p; 468*; -115; 20
SLC9A7; miR-2289-3p; 4109; -115; 20	*SYS1*; miR-2289-3p; 470*; -115; 20
SLC9A7; miR-2289-3p; 5461; -115; 20	*SYT13*; miR-1169-3p; 3130; -115; 20
SLFN11; miR-2289-3p; 405*; -115; 20	*SYT13*; miR-2289-3p; 3132; -115; 20
SLX4; miR-1927-5p; 6491; -113; 19	*SYT15*; miR-1791-5p; 2562; -102; 19
* - CDS, ** - 5'UTR	
