# Supplementary material for: Features of Complementary Interactions of BmiRNAs with mRNAs of Human Genes
Source: Int J Mol Sci. 2026 Sep 30;27(19):8773. doi: 10.3390/ijms27198773 (PMC13665148; doi:10.3390/ijms27198773)
Supplement: Supplementary file 1 [file ijms-27-08773-s001.zip › Table S18.pdf]

Table S18. Quantitative Characteristics of BmiRNA Binding to mRNAs of Genes Beginning with "T".

**Gene; BmiRNA; BS, nt; ΔG, kJ/mole; BmiRNA length, nt**	
TADA3; miR-1189-3p; 1926; -113; 20	*TEP1*; miR-531-5p; 8691; -108; 19
TADA3; miR-1441-3p; 1924; -123; 22	*TEP1*; miR-1608-5p; 8693; -108; 19
TADA3; miR-2289-3p; 1928; -115; 20	*TEP1*; miR-1641-5p; 8692; -102; 18
TAF1; miR-531-5p; 6352; -108; 19	*TEP1*; miR-1791-5p; 9075; -102; 19
TAF1; miR-1641-5p; 6353; -102; 18	*TERF1*; miR-1169-3p; 1743; -115; 20
TAF8; miR-531-5p; 2498; -108; 19	*TERF1*; miR-2289-3p; 1745; -115; 20
TAF8; miR-1641-5p; 2499; -102; 18	*TERF2*; miR-1189-3p; 1749; -113; 20
TAPBP; miR-1169-3p; 2509; -115; 20	*TERF2*; miR-1441-3p; 1747; -123; 22
TAPBP; miR-1169-3p; 2814; -115; 20	*TERF2*; miR-2289-3p; 1751; -115; 20
TAPBP; miR-2289-3p; 2511; -115; 20	*TES*; miR-2131-3p; 2330; -104; 20
TAPBP; miR-2289-3p; 2816; -115; 20	*TEX11*; miR-2289-3p; 143**; -115; 20
TBC1D15 miR-531-5p; 3962; -108; 19	*TSPAN14*; miR-531-5p; 4827; -108; 19
TBC1D15; miR-1169-3p; 3383; -115; 20	*TSPAN14*; miR-1641-5p; 4828; -102; 18
TBC1D15; miR-1641-5p; 3963; -102; 18	*TSPYL1*; miR-1791-5p; 2293; -102; 19
TBC1D15; miR-1791-5p; 3438; -102; 19	*TSTD3*; miR-2087-3p; 887; -102; 19
TBC1D15; miR-2289-3p; 3385; -115; 20	*TTC21B*; miR-1169-3p; 4515; -115; 20
TBC1D20; miR-1169-3p; 2844; -115; 20	*TTC21B*; miR-2289-3p; 4517; -115; 20
TBC1D24; miR-1169-3p; 5320; -115; 20	*TTC22*; miR-1379-5p; 1132*; -102; 19
TBC1D24; miR-1189-3p; 4457; -113; 20	*TTC23*; miR-1791-5p; 514**; -102; 19
TBC1D24; miR-1441-3p; 4455; -123; 22	*TTC39C*; miR-1169-3p; 4672; -115; 20
TBC1D24; miR-1791-5p; 4512; -102; 19	*TTC39C*; miR-1189-3p; 3943; -113; 20
TBC1D24; miR-2289-3p; 4459; -115; 20	*TTC39C*; miR-1441-3p; 3941; -123; 22
TBC1D24; miR-2289-3p; 5322; -115; 20	*TTC39C*; miR-2289-3p; 3945; -115; 20
TBC1D5; miR-1169-3p; 594**; -115; 20	*TTI2*; miR-1169-3p; 333**; -115; 20
TBCCD1; miR-1169-3p; 2322; -115; 20	*TTI2*; miR-2289-3p; 335**; -115; 20
TBCCD1; miR-2289-3p; 2324; -115; 20	*TTPAL*; miR-531-5p; 2668; -108; 19
TBRG1; miR-531-5p; 3530; -108; 19	*TTPAL*; miR-1641-5p; 2669; -102; 18
TBRG1; miR-1608-5p; 3532; -108; 19	*TTPAL*; miR-2087-3p; 5578; -102; 19
TBRG1; miR-1641-5p; 3531; -102; 18	*TUBD1*; miR-531-5p; 1898; -108; 19
TBXA2R; miR-1791-5p; 1805; -102; 19	*TUBD1*; miR-1641-5p; 1899; -102; 18
TBXA2R; miR-1927-5p; 2087; -113; 19	*TUFT1*; miR-1189-3p; 2503; -113; 20
TBXA2R; miR-2289-3p; 1752; -115; 20	*TUFT1*; miR-1441-3p; 2501; -123; 22
TCEANC2; miR-531-5p; 4057; -108; 19	*TUFT1*; miR-2289-3p; 2505; -115; 20
TCEANC2; miR-1297-3p; 2661; -100; 20	*TVP23C*; miR-1169-3p; 1136; -115; 20
TCEANC2; miR-1608-5p; 4059; -108; 19	*TVP23C*; miR-1169-3p; 1626; -115; 20
TCEANC2; miR-1641-5p; 4058; -102; 18	*TVP23C*; miR-1169-3p; 2639; -115; 20
TCEB1; miR-531-5p; 2193; -108; 19	*TVP23C*; miR-2289-3p; 1628; -115; 20
TCEB1; miR-1641-5p; 2194; -102; 18	*TVP23C*; miR-2289-3p; 2641; -115; 20
TCTN2; miR-1189-3p; 2557; -113; 20	*TXNL4B*; miR-1189-3p; 1417; -113; 20
TCTN2; miR-1441-3p; 2555; -123; 22	*TXNL4B*; miR-1441-3p; 1415; -123; 22
TCTN2; miR-1791-5p; 2315; -102; 19	*TXNL4B*; miR-2289-3p; 1419; -115; 20
TDRD1; miR-531-5p; 4113; -108; 19	*TYSND1*; miR-1189-3p; 2704; -113; 20
TDRD1; miR-1641-5p; 4114; -102; 18	*TYSND1*; miR-1441-3p; 2702; -123; 22
TENM4; miR-1379-5p; 11694; -102; 19	*TYW5*; miR-1641-5p; 4248; -102; 18
* - CDS, ** - 5'UTR	
