# Supplementary material for: Features of Complementary Interactions of BmiRNAs with mRNAs of Human Genes
Source: Int J Mol Sci. 2026 Sep 30;27(19):8773. doi: 10.3390/ijms27198773 (PMC13665148; doi:10.3390/ijms27198773)
Supplement: Supplementary file 1 [file ijms-27-08773-s001.zip › Table S19.pdf]

Table S19. Quantitative Characteristics of BmiRNA Binding to mRNAs of Genes Beginning with "U, V, W, X and Y".

**Gene; BmiRNA; BS, nt; ΔG, kJ/mol; BmiRNA length, nt**	
UACA;miR-2087-3p;6296;-102;19	*ULK2*;miR-1441-3p;7011;-123;22
UBA52;miR-1169-3p;1137;-115;20	*ULK2*;miR-2289-3p;7015;-115;20
UBA52;miR-2289-3p;1139;-115;20	*UNC13A*;miR-1608-5p;7298;-108;19
UBAP1;miR-1791-5p;311;CDS;-102;19	*UNC13A*;miR-1641-5p;7297;-102;18
UBE2G1;miR-1189-3p;2897;-113;20	*UNC45B*;miR-1379-5p;5002;-102;19
UBE2G1;miR-1441-3p;2895;-123;22	*UNKL*;miR-2087-3p;3192;-102;19
UBE2G1;miR-2289-3p;2899;-115;20	*UQCRQ*;miR-1189-3p;761;-113;20
UBE2G2;miR-531-5p;1602;-108;19	*UQCRQ*;miR-1441-3p;759;-123;22
UBE2G2;miR-531-5p;2639;-108;19	*UQCRQ*;miR-2131-3p;942;-104;20
UBE2G2;miR-1608-5p;1604;-108;19	*USP14*;miR-531-5p;2980;-108;19
UBE2G2;miR-1608-5p;2641;-108;19	*USP14*;miR-1641-5p;2981;-102;18
UBE2G2;miR-1641-5p;1603;-102;18	*USP15*;miR-1169-3p;6427;-115;20
UBE2G2;miR-1641-5p;2640;-102;18	*USP3*;miR-1169-3p;2920;-115;20
UBN2;miR-531-5p;8852;-108;19	*USP3*;miR-2289-3p;2922;-115;20
UBN2;miR-1641-5p;8853;-102;18	*USP33*;miR-1791-5p;3515;-102;19
UGDH;miR-1169-3p;1943;-115;20	*USP46*;miR-1189-3p;6185;-113;20
UGGT1;miR-531-5p;6919;-108;19	*USP46*;miR-1441-3p;6183;-123;22
UGGT1;miR-1189-3p;8395;-113;20	*USP49*;miR-2087-3p;2438;-102;19
UGGT1;miR-1441-3p;8393;-123;22	*USP9X*;miR-1189-3p;10423;-113;20
UGGT1;miR-1641-5p;6920;-102;18	*USP9X*;miR-1441-3p;10421;-123;22
UHMK1;miR-1169-3p;3840;-115;20	*USP9X*;miR-2289-3p;10425;-115;20
UHMK1;miR-2289-3p;3842;-115;20	*UST*;miR-531-5p;2095;-108;19
ULK2;miR-1189-3p;7013;-113;20	*UST*;miR-1641-5p;2096;-102;18
**Gene; BmiRNA; BS, nt; ΔG, kJ/mol; BmiRNA length, nt**	
VHL;miR-531-5p;2910;-108;19	*VPS53*;miR-1441-3p;3027;-123;22
VHL;miR-1169-3p;3422;-115;20	*VPS53*;miR-1641-5p;4197;-102;18
VHL;miR-1189-3p;1823;-113;20	*VPS53*;miR-1641-5p;5922;-102;18
VHL;miR-1441-3p;1821;-123;22	*VPS53*;miR-2289-3p;3031;-115;20
VHL;miR-1641-5p;2911;-102;18	*VSIG1*;miR-1131-5p;2494;-104;19
VHL;miR-2289-3p;1825;-115;20	*VSIG1*;miR-1791-5p;1746;-102;19
VMA21;miR-1169-3p;1788;-115;20	*VSIG10*;miR-1791-5p;2427;-102;19
VMA21;miR-1927-5p;3341;-113;19	*VSTM4*;miR-1608-5p;5464;-108;19
VMAC;miR-1791-5p;992;-102;19	*VWA2*;miR-531-5p;3593;-108;19
VPS13B;miR-1379-5p;13567;-102;19	*VWA2*;miR-1608-5p;3595;-108;19
VPS53;miR-531-5p;4196;-108;19	*VWA2*;miR-1641-5p;3594;-102;18
VPS53;miR-531-5p;5921;-108;19	*VWA2*;miR-2086-3p;3579;-138;25
VPS53;miR-1189-3p;3029;-113;20	
**Gene; BmiRNA; BS, nt; ΔG, kJ/mol; BmiRNA length, nt**	
WDR13;miR-2171-3p;76;5'UTR;-125;21	*WDR76*;miR-1791-5p;2329;-102;19
WDR13;miR-2171-5p;114;5'UTR;-123;21	*WDR87*;miR-1927-5p;9073;-113;19
WDR17;miR-531-5p;5361;-108;19	*WDR92*;miR-531-5p;1455;-108;19
WDR17;miR-1608-5p;5363;-108;19	*WDR92*;miR-1608-5p;1457;-108;19
WDR17;miR-1641-5p;5362;-102;18	*WDR92*;miR-1641-5p;1456;-102;18
WDR17;miR-1641-5p;6120;-102;18	*WNK3*;miR-1791-5p;6518;-102;19
WDR55;miR-1927-5p;2038;-113;19	*WWC1*;miR-531-5p;4901;-108;19
WDR5B;miR-1169-3p;2454;-115;20	*WWC1*;miR-1641-5p;4902;-102;18
WDR5B;miR-2289-3p;2456;-115;20	*WWC3*;miR-1169-3p;5518;-115;20
WDR74;miR-1169-3p;112;5'UTR;-115;20	*WWTR1*;miR-1791-5p;2482;-102;19
WDR74;miR-2289-3p;114;5'UTR;-115;20	
**Gene; BmiRNA; BS, nt; ΔG, kJ/mol; BmiRNA length, nt**	
XAF1;miR-531-5p;3080;-108;19	*XKR5*;miR-2289-3p;3322;-115;20
XAF1;miR-1608-5p;3082;-108;19	*XPNPEP3*;miR-531-5p;3024;-108;19
XAF1;miR-1641-5p;3081;-102;18	*XPNPEP3*;miR-1189-3p;5359;-113;20
XIAP;miR-531-5p;5299;-108;19	*XPNPEP3*;miR-1441-3p;5357;-123;22

XIAP;miR-1189-3p;3540;-113;20	*XPNPEP3*;miR-1641-5p;2624;-102;18
XIAP;miR-1441-3p;3538;-123;22	*XPNPEP3*;miR-1641-5p;3025;-102;18
XIAP;miR-1608-5p;5301;-108;19	*XPNPEP3*;miR-1791-5p;4476;-102;19
XIAP;miR-1641-5p;5300;-102;18	*XPNPEP3*;miR-2289-3p;5361;-115;20
XKR5;miR-1189-3p;3320;-113;20	*XRCC2*;miR-2289-3p;1009;-115;20
XKR5;miR-1441-3p;3318;-123;22	
**Gene; BmiRNA; BS, nt; ΔG, kJ/mole; BmiRNA length, nt**	
YAF2;miR-531-5p;2749;-108;19	*YIPF6*;miR-1189-3p;4821;-113;20
YAF2;miR-1608-5p;2751;-108;19	*YIPF6*;miR-1441-3p;4819;-123; 22
YAF2;miR-1641-5p;2750;-102;18	*YWHAB*;miR-1608-5p;1997;-108;19
YES1;miR-1169-3p;3207;-115;20	*YWHAB*;miR-1641-5p;1996;-102;18
YES1;miR-2289-3p;3209;-115;20	
