# Supplementary material for: Features of Complementary Interactions of BmiRNAs with mRNAs of Human Genes
Source: Int J Mol Sci. 2026 Sep 30;27(19):8773. doi: 10.3390/ijms27198773 (PMC13665148; doi:10.3390/ijms27198773)
Supplement: Supplementary file 1 [file ijms-27-08773-s001.zip › Table S2.pdf]

Table S2. Quantitative Characteristics of BmiRNA Binding to mRNAs of Genes Beginning with "B".

**Gene; BmiRNA; BS,nt; ΔG,kJ/mol; BmiRNA length,nt**	
B3GNT6; BmiR-531-5p; 2456; -108; 19	*BMP3*;BmiR-2289-3p;4581; 3'UTR -115; 20
BBS5; BmiR-1169-3p; 1768; -115; 20	*BMP7*; BmiR-754-5p; 2813; -119; 22
BBS5; BmiR-2289-3p; 1770; -115; 20	*BMP8A*; BmiR-1441-3p; 2133; -123; 22
BCAS4; BmiR-1927-5p; 585*; -113; 19	*BMP8A*; BmiR-2164-3p; 2134; -113; 20
BCDIN3D; BmiR-531-5p; 1946; -108; 19	*BMP8A*; BmiR-1189-3p; 2135; -113; 20
BCDIN3D; BmiR-1641-5p; 1947; -102; 18	*BMP8A*; BmiR-1169-3p; 4500; -115; 20
BCL10; BmiR-1169-3p; 2024; -115; 20	*BMP8A*; BmiR-2289-3p; 4502; -115; 20
BCL10; BmiR-2289-3p; 2026; -115; 20	*BMP8A*; BmiR-1379-5p; 4649; -102; 19
BCL2L15; BmiR-754-5p; 2722; -119; 22	*BMS1*; BmiR-531-5p; 5495; -108; 19
BCL2L15; BmiR-2164-3p; 1182; -113; 20	*BMS1*; BmiR-1641-5p; 5496; -102; 18
BCL2L15; BmiR-1441-3p; 1181; -123; 22	*BPI*; BmiR-1449-3p; 630*; -115; 21
BCL2L15; BmiR-1189-3p; 1183; -113; 20	*BRCA1*; BmiR-754-5p; 6484; -119; 22
BEND2; BmiR-1441-3p; 405*; -123; 22	*BRIP1*; BmiR-531-5p; 6814; -108; 19
BEND2; BmiR-2164-3p; 406*; -113; 20	*BRIP1*; BmiR-1641-5p; 6815; -102; 18
BEND2; BmiR-1189-3p; 407*; -113; 20	*BRIP1*; BmiR-1096-3p; 7202; -108; 20
BEND2; BmiR-2289-3p; 409*; -115; 20	*BRWD3*; BmiR-531-5p; 11807; -108;19
BIVM; BmiR-531-5p; 2969; -108; 19	*BRWD3*;BmiR-1641-5p;11808; -102;18
BIVM; BmiR-1641-5p; 2970; -102; 18	*BTN3A2*; BmiR-531-5p; 1956; -108; 19
BLOC1S3; BmiR-1379-5p; 1249; -102; 19	*BTN3A2*;BmiR-1641-5p;1957; -102; 18
BLOC1S2; BmiR-1791-5p; 2185; -102; 19	*BVES*; BmiR-531-5p; 3466; -108; 19
BLZF1; BmiR-531-5p; 2289; -108; 19	*BVES*; BmiR-1608-5p; 3468; -108; 19
BLZF1; BmiR-1641-5p; 2290; -102; 18	*BVES*; BmiR-1641-5p; 3467; -102; 18
BMP3; BmiR-1169-3p; 4579; -115; 20	

* - CDS; ** - 5'UTR.
