# Supplementary material for: Features of Complementary Interactions of BmiRNAs with mRNAs of Human Genes
Source: Int J Mol Sci. 2026 Sep 30;27(19):8773. doi: 10.3390/ijms27198773 (PMC13665148; doi:10.3390/ijms27198773)
Supplement: Supplementary file 1 [file ijms-27-08773-s001.zip › Table S3.pdf]

Table S3. Quantitative Characteristics of BmiRNA Binding to mRNAs of Genes Beginning with "C".

**Gene; BmiRNA; BS,nt; ΔG, kj/mol; BmiRNA length, nt.**	
C10orf111; BmiRNA; -1449-3p; 806; -115; 21	*CD68*; BmiRNA; -754-5p; 1470; -119; 22
C10orf32; BmiRNA; -531-5p; 768; -108; 19	*CD68*; BmiRNA; -1641-5p; 1649; -102; 18
C10orf32; BmiRNA; -1641-5p; 769; -102; 18	*CD82*; BmiRNA; -1641-5p; 1650; -102; 18
C10orf76; BmiRNA; -1791-5p; 2817; -102; 19	*CD84*; BmiRNA;-531-5p;3326;-108;19
C11orf31; BmiRNA; -1096-3p; 1252; -108; 20	*CD84*; BmiRNA;-531-5p;5165;-108;19
C11orf58; BmiRNA;-1169-3p;1935;-115;20	*CD84*; BmiRNA;-1641-5p;3327;-102;18
C11orf58; BmiRNA;-2289-3p;1937;-115;20	*CD84*; BmiRNA;-1641-5p;5166;-102;18
C11orf58; BmiRNA;-1791-5p;1990;-102;19	*CDC40*; BmiRNA;-531-5p;3092;-108;19
C11orf83; BmiRNA;-1441-3p;802;-123;22	*CDC40*; BmiRNA;-1641-5p;3093;-102;18
C11orf83; BmiRNA;-2164-3p;803;-113;20	*CDC42*; BmiRNA;-1169-3p;135**;-115;20
C11orf83; BmiRNA;-1189-3p;804;-113;20	*CDC42*; BmiRNA;-2289-3p;137**;-115;20
C11orf83; BmiRNA;-2289-3p;806;-115;20	*CDC6*; BmiRNA;-1449-3p;2284;-115;21
C12orf5; BmiRNA;-754-5p;6425;-119;22	*CDHR3*; BmiRNA;-531-5p;5107;-108;19
C12orf65; BmiRNA;-754-5p;1260;-119;22	*CDHR3*; BmiRNA;-754-5p;4950;-119;22
C14orf105; BmiRNA;-531-5p;1996;-108;19	*CDHR3*; BmiRNA;-1641-5p;5108;-102;18
C14orf105; BmiRNA;-1641-5p;1997;-102;18	*CEACAM8*; BmiRNA;-1169-3p;1488;-115;20
C14orf119; BmiRNA;-1169-3p;2088;-115;20	*CEACAM8*; BmiRNA;-2289-3p;1490;-115;20
C14orf132; BmiRNA;-754-5p;3889;-119;22	*CENPI*; BmiRNA;-1189-3p;19**;-113;20
C14orf132; BmiRNA;-531-5p;4044;-108;19	*CENPI*; BmiRNA;-1441-3p;17**;-123;22
C14orf132; BmiRNA;-1641-5p;4045;-102;18	*CENPI*; BmiRNA;-2164-3p;18**;-113;20
C14orf132; BmiRNA;-1608-5p;4046;-108;19	*CENP*; BmiRNA;-2289-3p;2**1;-115;20
C*15orf38*; BmiRNA;-1379-5p;1126;-102;19	*CENPL*; BmiRNA;-531-5p;2304;-108;19
C16orf54; BmiRNA;-1246-5p;2119;-117;21	*CENPL*; BmiRNA;-754-5p;2150;-119;22
C17orf75; BmiRNA;-531-5p;2116;-108;19	*CENPL*; BmiRNA;-1608-5p;2306;-108;19
C17orf75; BmiRNA;-1641-5p;2117;-102;18	*CENPL*; BmiRNA;-1641-5p;2305;-102;18
C17orf85; BmiRNA;-531-5p;6147;-108;19	*CENPN*; BmiRNA;-1620-3p;3654;-119;22
C17orf85; BmiRNA;-1641-5p;6148;-102;18	*CEP104*; BmiRNA;-754-5p;5080;-119;22
C17orf85; BmiRNA;-1608-5p;6149;-108;19	*CEP104*; BmiRNA;-1096-3p;5894;-108;20
C17orf85; BmiRNA;-1791-5p;5306;-102;19	*CEP104*; BmiRNA;-1927-5p;5759;-113;19
C19orf40; BmiRNA;-531-5p;1084;-108;19	*CEP44*; BmiRNA;-1096-3p;2477;-108;20
C19orf40; BmiRNA;-1641-5p;1085;-102;18	*CEP44*; BmiRNA;-1449-3p;2305;-115;21
C19orf40; BmiRNA;-1608-5p;1086;-108;19	*CEP68*; BmiRNA;-531-5p;4624;-108;19
C1QTNF2; BmiRNA;-531-5p;2347;-108;19	*CEP68*; BmiRNA;-1608-5p;4626;-108;19
C1QTNF2; BmiRNA;-1641-5p;2348;-102;18	*CEP68*; BmiRNA;-1641-5p;4625;-102;18
C1QTNF2; BmiRNA;-1608-5p;2349;-108;19	*CERK*; BmiRNA;-531-5p;3730;-108;19
C1QTNF3; BmiRNA;-1169-3p;2578;-115;20	*CERK*; BmiRNA;-1608-5p;3732;-108;19
C1QTNF3; BmiRNA;-2289-3p;2580;-115;20	*CERK*; BmiRNA;-1641-5p;3731;-102;18
C1orf174; BmiRNA;-1096-3p;1298;-108;20	*CFLAR*; BmiRNA;-531-5p;4589;-108;19
C1orf210; BmiRNA;-1449-3p;1117;-115;21	*CFLAR*; BmiRNA;-531-5p;6136;-108;19
C1orf210; BmiRNA;-1791-5p;1173;-102;19	*CFLAR*; BmiRNA;-1169-3p;3245;-115;20
C1orf212; BmiRNA;-1379-5p;1975;-102;19	*CFLAR*; BmiRNA;-1608-5p;4591;-108;19
C1orf212; BmiRNA;-1169-3p;2412;-115;20	*CFLAR*; BmiRNA;-1641-5p;4590;-102;18
C1orf212; BmiRNA;-2289-3p;2414;-115;20	*CFLAR*; BmiRNA;-1641-5p;6137;-102;18
C1orf212; BmiRNA;-1791-5p;2467;-102;19	CFLAR; BmiRNA;-1791-5p;3300;-102;19
C21orf62; BmiRNA;-2087-3p;1416;-102;19	CFLAR; BmiRNA;-2289-3p;3247;-115;20
C21orf62; BmiRNA;-531-5p;2523;-108;19	*CHDH*; BmiRNA;-531-5p;3099;-108;19
C21orf62; BmiRNA;-1641-5p;2524;-102;18	*CHDH*; BmiRNA;-1641-5p;3100;-102;18
C21orf62; BmiRNA;-1608-5p;2525;-108;19	*CHM*; BmiRNA;-531-5p;3471;-108;19
C22orf25; BmiRNA;-1441-3p;1726;-123;22	*CHM*; BmiRNA;-1641-5p;3472;-102;18
C22orf25; BmiRNA;-2164-3p;1727;-113;20	*CHP1*; BmiRNA;-1189-3p;1897;-113;20
C22orf25; BmiRNA;-1189-3p;1728;-113;20	*CHP1*; BmiRNA;-1189-3p;2241;-113;20
C22orf25; BmiRNA;-2289-3p;1730;-115;20	*CHP1*; BmiRNA;-1441-3p;1895;-123;22
C2CD2; BmiRNA;-1449-3p;4506;-115;21	*CHP1*; BmiRNA;-1441-3p;2239;-123;22
C2CD2; BmiRNA;-1791-5p;4554;-102;19	*CHP1*; BmiRNA;-2164-3p;1896;-113;20

C2CD4A; BmiRNA;-1441-3p;2869;-123;22	*CHP1*; BmiRNA;-2164-3p;2240;-113;20
C2CD4A; BmiRNA;-2164-3p;2870;-113;20	*CHP1*; BmiRNA;-2289-3p;2243;-115;20
C2CD4A; BmiRNA;-1189-3p;2871;-113;20	*CHRFAM7A*; BmiRNA;-1169-3p;3305;-115;20
C2orf18; BmiRNA;-1169-3p;1658;-115;20	*CHRFAM7A*; BmiRNA;-2289-3p;3307;-115;20
C2orf18; BmiRNA;-2289-3p;1660;-115;20	*CHRNA5*; BmiRNA;-2087-3p;2939;-102;19
C2orf18; BmiRNA;-1096-3p;1820;-108;20	*CHRNA7*; BmiRNA;-1169-3p;3037;-115;20
C3orf33; BmiRNA;-1449-3p;1400;-115;21	*CHRNA7*; BmiRNA;-2289-3p;3039;-115;20
C3orf33; BmiRNA;-1791-5p;1458;-102;19	*CHST6*; BmiRNA;-754-5p;3948;-119;22
C3orf62; BmiRNA;-531-5p;3362;-108;19	*CHST6*; BmiRNA;-1169-3p;3470;-115;20
C3orf62; BmiRNA;-1641-5p;3363;-102;18	*CHST6*; BmiRNA;-2289-3p;3472;-115;20
C3orf62; BmiRNA;-1608-5p;3364;-108;19	*CHST6*; BmiRNA;-2289-3p;5858;-115;20
C3orf70; BmiRNA;-1441-3p;4690;-123;22	*CHURC1*; BmiRNA;-531-5p;2554;-108;19
C3orf70; BmiRNA;-2164-3p;4691;-113;20	*CHURC1*; BmiRNA;-1641-5p;2555;-102;18
C3orf70; BmiRNA;-1189-3p;4692;-113;20	*CISD2*; BmiRNA;-531-5p;3884;-108;19
C3orf70; BmiRNA;-2289-3p;4694;-115;20	*CISD2*; BmiRNA;-1608-5p;3886;-108;19
C3orf72; BmiRNA;-1169-3p;3146;-115;20	*CISD2*; BmiRNA;-1641-5p;3885;-102;18
C3orf72; BmiRNA;-2289-3p;3148;-115;20	*CLCC1*; BmiRNA;-1189-3p;3303;-113;20
C4orf19; BmiRNA;-531-5p;2296;-108;19	*CLCC1*; BmiRNA;-1441-3p;3301;-123;22
C4orf19; BmiRNA;-1641-5p;2297;-102;18	*CLCC1*; BmiRNA;-2164-3p;3302;-113;20
C4orf34; BmiRNA;-754-5p;1459;-119;22	*CLCC1*; BmiRNA;-2289-3p;3305;-115;20
C3orf62; BmiRNA;-531-5p;3362;-108;19	*CHST6*; BmiRNA;-1169-3p;3470;-115;20
C3orf62; BmiRNA;-1641-5p;3363;-102;18	*CHST6*; BmiRNA;-2289-3p;3472;-115;20
C3orf62; BmiRNA;-1608-5p;3364;-108;19	*CHST6*; BmiRNA;-2289-3p;5858;-115;20
C3orf70; BmiRNA;-1441-3p;4690;-123;22	*CHURC1*; BmiRNA;-531-5p;2554;-108;19
C3orf70; BmiRNA;-2164-3p;4691;-113;20	*CHURC1*; BmiRNA;-1641-5p;2555;-102;18
C3orf70; BmiRNA;-1189-3p;4692;-113;20	*CISD2*; BmiRNA;-531-5p;3884;-108;19
C3orf70; BmiRNA;-2289-3p;4694;-115;20	*CISD2*; BmiRNA;-1608-5p;3886;-108;19
C3orf72; BmiRNA;-1169-3p;3146;-115;20	*CISD2*; BmiRNA;-1641-5p;3885;-102;18
C3orf72; BmiRNA;-2289-3p;3148;-115;20	*CLCC1*; BmiRNA;-1189-3p;3303;-113;20
C4orf19; BmiRNA;-531-5p;2296;-108;19	*CLCC1*; BmiRNA;-1441-3p;3301;-123;22
C4orf19; BmiRNA;-1641-5p;2297;-102;18	*CLCC1*; BmiRNA;-2164-3p;3302;-113;20
C4orf34; BmiRNA;-754-5p;1459;-119;22	*CLCC1*; BmiRNA;-2289-3p;3305;-115;20
C5orf24; BmiRNA;-531-5p;1948;-108;19	*CLEC7A*; BmiRNA;-1189-3p;1230;-113;20
C5orf24; BmiRNA;-1641-5p;1949;-102;18	*CLEC7A*; BmiRNA;-2289-3p;1232;-115;20
C5orf28; BmiRNA;-754-5p;2524;-119;22	*CLN8*; BmiRNA;-754-5p;4688;-119;22
C5orf64; BmiRNA;-1169-3p;1192;-115;20	*CLUAP1*; BmiRNA;-531-5p;2180;-108;19
C5orf64; BmiRNA;-2289-3p;1194;-115;20	*CLUAP1*; BmiRNA;-1641-5p;2181;-102;18
C6orf225; BmiRNA;-754-5p;2168;-119;22	*CMBL*; BmiRNA;-754-5p;1888;-119;22
C6orf25; BmiRNA;-1169-3p;1204;-115;20	*CNDP2*; BmiRNA;-531-5p;2403;-108;19
C7orf73; BmiRNA;-1169-3p;2050;-115;20	*CNDP2*; BmiRNA;-1641-5p;2404;-102;18
C7orf73; BmiRNA;-2289-3p;2052;-115;20	*CNEP1R1*; BmiRNA;-1791-5p;1242;-102;19
C8orf31; BmiRNA;-531-5p;1835;-108;19	*CNGA1*; BmiRNA;-1169-3p;159**;-115;20
C8orf31; BmiRNA;-1641-5p;1836;-102;18	*CNGA1*; BmiRNA;-2289-3p;161**;-115;20
C8orf86; BmiRNA;-2087-3p;1474;-102;19	*CNN2*; BmiRNA;-1169-3p;2007;-115;20
C9orf3; BmiRNA;-1169-3p;2688;-115;20	*CNN2*; BmiRNA;-2289-3p;2009;-115;20
C9orf3; BmiRNA;-2289-3p;2690;-115;20	*CNNM1*; BmiRNA;-754-5p;4423;-119;22
C9orf5; BmiRNA;-531-5p;4338;108;19	*CNNM1*; BmiRNA;-2087-3p;4494;-102;19
C9orf5; BmiRNA;-1641-5p;4339;-102;18	*COMMD2*; BmiRNA;-531-5p;1054;-108;19
C9orf91; BmiRNA;-1441-3p;2767;-123;22	*COMMD2*; BmiRNA;-1641-5p;1055;-102;18
C9orf91; BmiRNA;-2164-3p;2768;-113;20	*COMMD2*; BmiRNA;-2087-3p;1002;-102;19
C9orf91; BmiRNA;-1189-3p;2769;-113;20	*COQ10B*; BmiRNA;-1608-5p;1494;-108;19
C9orf91; BmiRNA;-2289-3p;2771;-115;20	*CORO2A*; BmiRNA;-754-5p;2613;-119;22
CA13; BmiRNA;-1096-3p;2916;-108;20	*CORO2A*; BmiRNA;-754-5p;2749;-119;22
CA5B; BmiRNA;-531-5p;2157;-108;19	*COX18*; BmiRNA;-754-5p;1336;-119;22
CA5B; BmiRNA;-1641-5p;2158;-102;18	*COX18*; BmiRNA;-1189-3p;1818;-113;20
CA5B; BmiRNA;-1608-5p;2159;-108;19	*COX18*; BmiRNA;-1441-3p;1816;-123;22
CA5B; BmiRNA;-2289-3p;2336;-115;20	*COX18*; BmiRNA;-2087-3p;1442;-102;19

CABP4; BmiRNA;-531-5p;3190;-108;19	*COX18*; BmiRNA;-2164-3p;1817;-113;20
CABP4; BmiRNA;-1641-5p;3191;-102;18	*COX18*; BmiRNA;-2289-3p;1820;-115;20
CACNA2D4; BmiRNA;-2455-3p;207**;-115;21	*COX6B2*; BmiRNA;-1096-3p;1430;-108;20
CACNA2D4; BmiRNA;-2455-5p;245*; -127;22	*CPM*; BmiRNA;-754-5p;2770;-119;22
CACNG8; BmiRNA;-531-5p;5233;-108;19	*CPM*; BmiRNA;-2087-3p;2877;-102;19
CACNG8; BmiRNA;-1641-5p;5234;-102;18	*CPPED1*; BmiRNA;-531-5p;2822;-108;19
CACNG8; BmiRNA;-2083-3p;5870;-106;20	*CPPED1*; BmiRNA;-754-5p;2667;-119;22
CACUL1; BmiRNA;-1441-3p;4047;-123;22	*CPPED1*; BmiRNA;-1189-3p;4849;-113;20
CACUL1; BmiRNA;-2164-3p;4048;-113;20	*CPPED1*; BmiRNA;-1441-3p;4847;-123;22
CACUL1; BmiRNA;-1189-3p;4049;-113;20	*CPPED1*; BmiRNA;-1608-5p;2824;-108;19
CALN1; BmiRNA;-531-5p;6161;-108;19	*CPPED1*; BmiRNA;-1641-5p;2823;-102;18
CALN1; BmiRNA;-1641-5p;6162;-102;18	*CPPED1*; BmiRNA;-2164-3p;4848;-113;20
CALN1; BmiRNA;-1608-5p;6163;-108;19	*CPS1*; BmiRNA;-2289-3p;5287;-115;20
CAMK1D; BmiRNA;-531-5p;2198;-108;19	*CPSF2*; BmiRNA;-1096-3p;3987;-108;20
CAMK1D; BmiRNA;-1641-5p;2199;-102;18	*CPSF2*; BmiRNA;-1189-3p;3816;-113;20
CAMKK2; BmiRNA;-1641-5p;43**;-102;18	*CPSF2*; BmiRNA;-1441-3p;3814;-123;22
CAMKK2; BmiRNA;-1608-5p;44**;-108;19	*CPSF2*; BmiRNA;-1791-5p;4698;-102;19
CARD6; BmiRNA;-1449-3p;3371;-115;21	*CPSF2*; BmiRNA;-2164-3p;3815;-113;20
CARD8; BmiRNA;-1189-3p;4314;-113;20	*CPT1A*; BmiRNA;-531-5p;4668;-108;19
CARHSP1; BmiRNA;-1441-3p;1894;-123;22	*CPT1A*; BmiRNA;-1169-3p;4046;-115;20
CARHSP1; BmiRNA;-2164-3p;1895;-113;20	*CPT1A*; BmiRNA;-1641-5p;4669;-102;18
CARHSP1; BmiRNA;-1189-3p;1896;-113;20	*CPT1A*; BmiRNA;-1791-5p;4101;-102;19
CARHSP1; BmiRNA;-2289-3p;1898;-115;20	*CR1*; BmiRNA;-1189-3p;9582;-113;20
CASP8; BmiRNA;-1169-3p;1879;-115;20	*CR1*; BmiRNA;-2289-3p;8361;-115;20
CASP8; BmiRNA;-2289-3p;1881;-115;20	*CRCP*; BmiRNA;-754-5p;2097;-119;22
CBFA2T2; BmiRNA;-1169-3p;3749;-115;20	*CRKL*; BmiRNA;-1096-3p;2199;-108;20
CBFA2T2; BmiRNA;-2289-3p;3751;-115;20	*CRKL*; BmiRNA;-1189-3p;2029;-113;20
CBFA2T2; BmiRNA;-531-5p;4526;-108;19	*CRKL*; BmiRNA;-1441-3p;2027;-123;22
CBFA2T2; BmiRNA;-1641-5p;4527;-102;18	*CRKL*; BmiRNA;-2164-3p;2028;-113;20
CBFA2T2; BmiRNA;-1608-5p;4528;-108;19	*CRKL*; BmiRNA;-2289-3p;2031;-115;20
CBL; BmiRNA;-1169-3p;7748;-115;20	*CRTAM*; BmiRNA;-531-5p;1877;-108;19
CBLL1; BmiRNA;-1354-5p;228;5'UTR;-127;22	*CRTAM*; BmiRNA;-1608-5p;1879;-108;19
CBX5; BmiRNA;-2289-3p;4483;-115;20	*CRTAM*; BmiRNA;-1641-5p;1878;-102;18
CBX5; BmiRNA;-1791-5p;4536;-102;19	*CRTAM*; BmiRNA;-2087-3p;1825;-102;19
CBX5; BmiRNA;-2289-3p;7059;-115;20	*CRTAP*; BmiRNA;-1449-3p;1570;-115;21
CBX5; BmiRNA;-1169-3p;8492;-115;20	*CRX*; BmiRNA;-754-5p;3522;-119;22
CBX5; BmiRNA;-2289-3p;8494;-115;20	*CRX*; BmiRNA;-1169-3p;1578;-115;20
CBX5; BmiRNA;-1927-5p;8530;-113;19	*CRX*; BmiRNA;-2289-3p;1580;-115;20
CC2D1B; BmiRNA;-754-5p;5286;-119;22	*CSNK1G1*; BmiRNA;-531-5p;4389;-108;19
CC2D1B; BmiRNA;-1791-5p;4440;-102;19	*CSNK1G1*; BmiRNA;-754-5p;4233;-119;22
CC2D1B; BmiRNA;-531-5p;5443;-108;19	*CSNK1G1*; BmiRNA;-1608-5p;4391;-108;19
CC2D1B; BmiRNA;-1641-5p;5444;-102;18	*CSNK1G1*; BmiRNA;-1641-5p;4390;-102;18
CCBE1; BmiRNA;-531-5p;3536;-108;19	*CTSB*; BmiRNA;-2289-3p;2417;-115;20
CCBE1; BmiRNA;-1641-5p;3537;-102;18	*CTSS*; BmiRNA;-531-5p;2338;-108;19
CCDC125; BmiRNA;-2087-3p;2307;-102;19	*CTSS*; BmiRNA;-1096-3p;3108;-108;20
CCDC125; BmiRNA;-2451-3p;3031;-102;18	*CTSS*; BmiRNA;-1169-3p;2937;-115;20
CCDC137; BmiRNA;-754-5p;1552;-119;22	*CTSS*; BmiRNA;-1641-5p;2339;-102;18
CCDC152; BmiRNA;-531-5p;2930;-108;19	*CTSS*; BmiRNA;-2289-3p;2939;-115;20
CCDC152; BmiRNA;-1641-5p;2931;-102;18	*CWF19L1*; BmiRNA;-754-5p;2304;-119;22
CCDC170; BmiRNA;-531-5p;3050;-108;19	*CXADR*; BmiRNA;-1189-3p;3798;-113;20
CCDC170; BmiRNA;-1608-5p;3871;-108;19	*CXADR*; BmiRNA;-1441-3p;3796;-123;22
CCDC170; BmiRNA;-1641-5p;3051;-102;18	*CXADR*; BmiRNA;-2164-3p;3797;-113;20
CCDC170; BmiRNA;-1641-5p;3870;-102;18	*CXADR*; BmiRNA;-2289-3p;3800;-115;20
CCDC30; BmiRNA;-531-5p;3058;-108;19	*CXCL16*; BmiRNA;-1791-5p;1728;-102;19
CCDC30; BmiRNA;-1641-5p;3059;-102;18	*CXCL16*; BmiRNA;-2289-3p;1675;-115;20
CCDC8; BmiRNA;-1169-3p;2663;-115;20	*CXorf36*; BmiRNA;-754-5p;2933;-119;22
CCDC8; BmiRNA;-1246-5p;2649;-117;21	*CXorf38*; BmiRNA;-531-5p;2098;-108;19

CCDC8; BmiRNA;-2289-3p;2665;-115;20	*CXorf38*; BmiRNA;-1641-5p;2099;-102;18
CCDC90A; BmiRNA;-531-5p;1499;-108;19	*CXorf38*; BmiRNA;-2087-3p;1448;-102;19
CCDC90A; BmiRNA;-1608-5p;1501;-108;19	*CXorf56*; BmiRNA;-1927-5p;1086;-113;19
CCDC90A; BmiRNA;-1641-5p;1500;-102;18	*CXorf56*; BmiRNA;-2083-3p;1272;-106;20
CCDC90A; BmiRNA;-1791-5p;2696;-102;19	*CYB561D1*; BmiRNA;-1791-5p;4131;-102;19
CCL16; BmiRNA;-1189-3p;685;-113;20	*CYB561D1*; BmiRNA;-2289-3p;4078;-115;20
CCL16; BmiRNA;-1441-3p;683;-123;22	*CYB5R3*; BmiRNA;-1169-3p;2492;-115;20
CCL16; BmiRNA;-2164-3p;684;-113;20	*CYB5R3*; BmiRNA;-1791-5p;2371;-102;19
CCL22; BmiRNA;-1169-3p;1570;-115;20	*CYB5R3*; BmiRNA;-2289-3p;2494;-115;20
CCL22; BmiRNA;-2289-3p;1572;-115;20	*CYCS*; BmiRNA;-754-5p;4699;-119;22
CCL5; BmiRNA;-531-5p;741;-108;19	*CYCS*; BmiRNA;-1096-3p;2449;-108;20
CCL5; BmiRNA;-1641-5p;742;-102;18	*CYCS*; BmiRNA;-1449-3p;3085;-115;21
CCNB1IP1; BmiRNA;-531-5p;1611;-108;19	*CYCS*; BmiRNA;-1608-5p;4552;-108;19
CCNB1IP1; BmiRNA;-1608-5p;1613;-108;19	*CYCS*; BmiRNA;-1641-5p;4551;-102;18
CCNB1IP1; BmiRNA;-1641-5p;1612;-102;18	*CYCS*; BmiRNA;-1791-5p;3142;-102;19
CCNDBP1; BmiRNA;-1096-3p;2971;-108;20	*CYCS*; BmiRNA;-2087-3p;4802;-102;19
CCNYL1; BmiRNA;-2289-3p;2249;-115;20	*CYP1A2*; BmiRNA;-2289-3p;2385;-115;20
CCSER2; BmiRNA;-1791-5p;4675;-102;19	*CYP20A1*; BmiRNA;-531-5p;3452;-108;19
CD109; BmiRNA;-531-5p;7067;-108;19	*CYP20A1*; BmiRNA;-531-5p;3763;-108;19
CD109; BmiRNA;-1641-5p;7068;-102;18	*CYP20A1*; BmiRNA;-754-5p;2884;-119;22
CD209; BmiRNA;-1791-5p;2906;-102;19	*CYP20A1*; BmiRNA;-1379-5p;20**;-102;19
CD209; BmiRNA;-2289-3p;2853;-115;20	*CYP20A1*; BmiRNA;-1608-5p;3454;-108;19
CD300LG; BmiRNA;-1169-3p;2224;-115;20	*CYP20A1*; BmiRNA;-1641-5p;3453;-102;18
CD300LG; BmiRNA;-2289-3p;2226;-115;20	*CYP20A1*; BmiRNA;-1641-5p;3764;-102;18
CD302; BmiRNA;-1096-3p;3157;-108;20	*CYP20A1*; BmiRNA;-2087-3p;4045;-102;19
CD3EAP; BmiRNA;-1189-3p;2743;-113;20	*CYP27C1*; BmiRNA;-754-5p;3895;-119;22
CD3EAP; BmiRNA;-1441-3p;2741;-123;22	*CYP2B6*; BmiRNA;-1169-3p;1738;;-115;20
CD3EAP; BmiRNA;-2164-3p;2742;-113;20	*CYP2B6*; BmiRNA;-2289-3p;1740;-115;20
CD3G; BmiRNA;-1791-5p;1154;-102;19	*CYP51A1*; BmiRNA;-1169-3p;2550;-115;20
CD68; BmiRNA;-531-5p;1648;-108;19	

* - CDS; ** - 5'UTR.
