# Supplementary material for: Features of Complementary Interactions of BmiRNAs with mRNAs of Human Genes
Source: Int J Mol Sci. 2026 Sep 30;27(19):8773. doi: 10.3390/ijms27198773 (PMC13665148; doi:10.3390/ijms27198773)
Supplement: Supplementary file 1 [file ijms-27-08773-s001.zip › Table S4.pdf]

Table S4. Quantitative Characteristics of BmiRNA Binding to mRNAs of Genes Beginning with "D".

Gene; BmiRNA; BS,nt; ΔG,kJ/mol; BmiRNA length,nt	
DAND5; BmiRNA-1169-3p; 881; -115;2 0	*DFFB*; BmiRNANA-1096-3p; 1693; -108; 20
DAND5; BmiRNA-2289-3p; 883; -115; 20	*DGKB*; BmiRNA-531-5p; 6216;-108; 19
DAP3; BmiRNA-531-5p; 2070; -108; 19	*DGKB*; BmiRNA-1641-5p; 6217; -102; 18
DAP3; BmiRNA-1641-5p; 2071; -102; 18	*DGKE*; BmiRNA-1608-5p; 6657; -108; 19
DAP3; BmiRNA-1608-5p; 2072; -108;19	*DGKE*; BmiRNA-1641-5p; 6656; -102; 18
DBT;BmiRNA-754-5p;1858;-119;22	*DIP2A*;BmiRNA-1641-5p;5757;-102;18
DBT;BmiRNA-2087-3p;1962;-102;19	*DIS3*;BmiRNA-531-5p;5738;-108;19
DBT;BmiRNA-531-5p;3341;-108;19	*DIS3*;BmiRNA-1641-5p;5739;-102;18
DBT;BmiRNA-531-5p;10151;-108;19	*DIS3*;BmiRNA-2087-3p;5510;-102;19
DBT;BmiRNA-1641-5p;3342;-102;18	*DIS3*;BmiRNA-2087-3p;5686;-102;19
DBT;BmiRNA-1641-5p;10152;-102;18	*DLGAP4*;BmiRNA-754-5p;1125*;-119;22
DCAF16;BmiRNA-754-5p;3860;-119;22	*DMBX1*;BmiRNA-531-5p;2827;-108;19
DCAF16;BmiRNA-531-5p;4016;-108;19	*DMBX1*;BmiRNA-1641-5p;2828;-102;18
DCAF16;BmiRNA-1641-5p;4017;-102;18	*DNAJC18*;BmiRNA-754-5p;4322;-119;22
DCAF16;BmiRNA-1608-5p;4018;-108;19	*DNAJC21*;BmiRNA-531-5p;3256;-108;19
DCAF4L1;BmiRNA-1169-3p;2785;-115;20	*DNAJC21*;BmiRNA-1641-5p;3257;-102;18
DCAF4L1;BmiRNA-2289-3p;2787;-115;20	*DNAJC22*;BmiRNA-531-5p;1784;-108;19
DCAF7;BmiRNA-531-5p;4439;-108;19	*DNAJC22*;BmiRNA-1608-5p;1786;-108;19
DCAF7;BmiRNA-1641-5p;4440;-102;18	*DNAJC22*;BmiRNA-1641-5p;1785;-102;18
DCDC2;BmiRNA-1096-3p;3662;-108;20	*DNAJC24*;BmiRNA-1096-3p;1395;-108;20
DCP2;BmiRNA-1449-3p;4882;-115;21	*DNAJC30*;BmiRNA-1169-3p;1520;-115;20
DCP2;BmiRNA-531-5p;9329;-108;19	*DNAJC30*;BmiRNA-1791-5p;1575;-102;19
DCP2;BmiRNA-1641-5p;9330;-102;18	*DNAL1*;BmiRNA-1096-3p;4358;-108;20
DCTN5;BmiRNA-531-5p;6699;-108;19	*DNASE2*;BmiRNA-754-5p;1611;-119;22
DCTN5;BmiRNA-1641-5p;6700;-102;18	*DPPA4*;BmiRNA-1169-3p;1310;-115;20
DCUN1D2;BmiRNA-754-5p;1754;-119;22	*DPY19L4*;BmiRNA-1608-5p;5202;-108;19
DDA1;BmiRNA-2289-3p;1797;-115;20	*DPY19L4*;BmiRNA-2131-3p;4855;-104;20
DDA1;BmiRNA-2289-3p;1953;-115;20	*DSEL*;BmiRNA-531-5p;7075;-108;19
DDX19A;BmiRNA-531-5p;2714;-108;19	*DSEL*;BmiRNA-1641-5p;7076;-102;18
DDX19A;BmiRNA-1641-5p;2715;-102;18	*DSEL*;BmiRNA-1169-3p;8538;-115;20
DDX51;BmiRNA-531-5p;2634;-108;19	*DSTYK*;BmiRNA-531-5p;4825;-108;19
DDX51;BmiRNA-1641-5p;2635;-102;18	*DSTYK*;BmiRNA-1608-5p;4827;-108;19
DDX51;BmiRNA-531-5p;2941;-108; 19	*DSTYK*;BmiRNA-531-5p;7207;-108;19
DDX51;BmiRNA-1641-5p;2942;-102;18	*DSTYK*;BmiRNA-1608-5p;7209;-108;19
DDX51;BmiRNA-1608-5p;2636;-108;19	*DSTYK*;BmiRNA-754-5p;3625;-119;22
DDX51;BmiRNA-1608-5p;2943;-108;19	*DSTYK*;BmiRNA-1641-5p;4826;-102;18
DEM1;BmiRNA-1608-5p;2163;-108;19	*DSTYK*;BmiRNA-1641-5p;7208;-102;18
DENND5B;BmiRNA-1131-5p;6790;-104;19	*DSTYK*;BmiRNA-2087-3p;3730;-102;19
DENND6A;BmiRNA-754-5p;2754;-119;22	*DTWD2*;BmiRNA-531-5p;2435;-108;19
DENND6A;BmiRNA-1449-3p;3423;-115;21	*DTWD2*;BmiRNA-1641-5p;2436;-102;18
DENR;BmiRNA-1927-5p;1239;-113;19	*DTWD2*;BmiRNA-1169-3p;2708;-115;20
DENR;BmiRNA-531-5p;3021;-108;19	*DTWD2*;BmiRNA-2289-3p;2710;-115;20
DENR;BmiRNA-1641-5p;3022;-102;18	*DTX3L*;BmiRNA-2289-3p;4444;-115;20
DENR;BmiRNA-1608-5p;3023;-108;19	*DUSP19*;BmiRNA-1169-3p;3302;-115;20
DESI1;BmiRNA-754-5p;2049;-119;22	*DUSP19*;BmiRNA-1791-5p;1702;-102;19
DFFA;BmiRNA-531-5p;1724;-108;19	*DUSP19*;BmiRNA-2289-3p;3304;-115;20
DFFA;BmiRNA-1641-5p;1725;-102;18	*DUXA*;BmiRNA-531-5p;2377;-108;19
DFFA;BmiRNA-531-5p;2026;-108;19	*DUXA*;BmiRNA-1641-5p;1741;-102;18
DFFA;BmiRNA-1641-5p;2027;-102;18	*DUXA*;BmiRNA-1641-5p;2378;-102;18
DFFA;BmiRNA-1641-5p;2973;-102;18	*DYDC1*;BmiRNA-1449-3p;65**;-115;21

* - CDS; ** - 5'UTR.
