# Supplementary material for: Features of Complementary Interactions of BmiRNAs with mRNAs of Human Genes
Source: Int J Mol Sci. 2026 Sep 30;27(19):8773. doi: 10.3390/ijms27198773 (PMC13665148; doi:10.3390/ijms27198773)
Supplement: Supplementary file 1 [file ijms-27-08773-s001.zip › Table S5.pdf]

Table S5. Quantitative Characteristics of BmiRNA Binding to mRNAs of Genes Beginning with "E".

**Gene; BmiRNA; BS, nt; ΔG, kJ/mol; BmiRNA length, nt**	
E2F2;BmiRNA-1169-3p;4127;-115;20	*ENPP6*;BmiRNA-531-5p;3108;-108;19
E2F2;BmiRNA-2289-3p;4129;-115;20	*ENPP6*;BmiRNA-1641-5p;3109;-102;18
ECE1;BmiRNA-531-5p;3843;-108;19	*ENPP6*;BmiRNA-1608-5p;3110;3'UTR-108;19
ECE1;BmiRNA-1641-5p;3844;-102;18	*ENTPD1*;BmiRNA-1169-3p;4763;-115;20
ECT2;BmiRNA-1791-5p;248**;-102;19	*ENTPD1*;BmiRNA-2083-3p;4988;-106;20
EEF1A1;BmiRNA-1096-3p;2845;-108;20	*ENTPD4*;BmiRNA-531-5p;2492;-108;19
EEF2K;BmiRNA-531-5p;5892;-108;19	*ENTPD4*;BmiRNA-1641-5p;2493;-102;18
EEF2K;BmiRNA-1641-5p;5893;-102;18	*ENTPD7*;BmiRNA-2164-3p;3682;-113;20
EEF2K;BmiRNA-1189-3p;2987;-113;20	*EOGT*;BmiRNA-1189-3p;2051;-113;20
EEF2K;BmiRNA-1441-3p;2985;-123;22	*EOGT*;BmiRNA-1189-3p;2217;-113;20
EEF2K;BmiRNA-2164-3p;2986;-113;20	*EOGT*;BmiRNA-1441-3p;2049;-123;22
EFR3B;BmiRNA-1379-5p;4205;-102;19	*EOGT*;BmiRNA-1441-3p;2215;-123;22
EHD2;BmiRNA-1169-3p;2762;-115;20	*EOGT*;BmiRNA-2164-3p;2050;-113;20
EIF2AK2;BmiRNA-1096-3p;2635;-108;20	*EOGT*;BmiRNA-2164-3p;2216;-113;20
EIF2AK2;BmiRNA-1449-3p;2464;-115;21	*EOGT*;BmiRNA-2289-3p;2053;-115;20
EIF2S3;BmiRNA-1189-3p;1772;-113;20	*EOGT*;BmiRNA-2289-3p;2219;-115;20
EIF2S3;BmiRNA-1441-3p;1770;-123;22	*EPGN*;BmiRNA-754-5p;1647;-119;22
EIF2S3;BmiRNA-1791-5p;1818;-102;19	*ERC1*;BmiRNA-531-5p;6689;-108;19
EIF2S3;BmiRNA-2164-3p;1771;-113;20	*ERC1*;BmiRNA-1641-5p;6690;-102;18
EIF2S3;BmiRNA-2289-3p;1774;-115;20	*ERCC1*;BmiRNA-1791-5p;2666;-102;19
EIF4E;BmiRNA-2289-3p;4739;-115;20	*ERCC4*;BmiRNA-1169-3p;4414;-115;20
ELK4;BmiRNA-1189-3p;3723;-113;20	*ERCC4*;BmiRNA-2289-3p;4416;-115;20
ELK4;BmiRNA-1441-3p;3721;-123;22	*ERCC6L2*;BmiRNA-531-5p;3899;-108;19
ELK4;BmiRNA-2164-3p;3722;-113;20	*ERCC6L2*;BmiRNA-1641-5p;3900;-102;18
ELK4;BmiRNA-2289-3p;3725;-115;20	*ESYT2*;BmiRNA-531-5p;5595;-108;19
ELMSAN1;BmiRNA-1169-3p;6561;-115;20	*ESYT2*;BmiRNA-1641-5p;5596;-102;18
ELP3;BmiRNA-1189-3p;2676;-113;20	*ESYT2*;BmiRNA-1608-5p;5597;-108;19
ELP3;BmiRNA-1441-3p;2674;-123;22	*EVC*;BmiRNA-1169-3p;5367;-115;20
ELP3;BmiRNA-2164-3p;2675;-113;20	*EVC*;BmiRNA-2289-3p;5369;-115;20
ELP3;BmiRNA-2289-3p;2678;-115;20	*EVI5*;BmiRNA-1791-5p;4728;-102;19
EMC1;BmiRNA-754-5p;6432;-119;22	*EXD1*;BmiRNA-1927-5p;2437;-113;19
EMC1;BmiRNA-1169-3p;4840;-115;20	*EXOSC2*;BmiRNA-2200-3p;1594;-117;22
EMC1;BmiRNA-2289-3p;4842;-115;20	*EXOSC6*;BmiRNA-1449-3p;1282;-115;21
EMP2;BmiRNA-531-5p;2212;-108;19	*EXPH5*;BmiRNA-1189-3p;7133;-113;20
EMP2;BmiRNA-754-5p;2415;-119;22	*EXPH5*;BmiRNA-1441-3p;7131;-123;22
EMP2;BmiRNA-1641-5p;2213;-102;18	*EXPH5*;BmiRNA-1449-3p;8647;-115;21
EMP2;BmiRNA-1169-3p;3694;-115;20	*EXPH5*;BmiRNA-1791-5p;7188;-102;19
EMP2;BmiRNA-2289-3p;3696;-115;20	*EXPH5*;BmiRNA-1791-5p;8704;-102;19
ENPP1;BmiRNA-1189-3p;6280;-113;20	*EXPH5*;BmiRNA-1927-5p;7171;-113;19
ENPP1;BmiRNA-1441-3p;6278;-123;22	*EXPH5*;BmiRNA-2164-3p;7132;-113;20
ENPP1;BmiRNA-2164-3p;6279;-113;20	*EXPH5*;BmiRNA-2289-3p;7135;-115;20
ENPP1;BmiRNA-2289-3p;6282;-115;20	

* - CDS; ** - 5'UTR.
