# Supplementary material for: Features of Complementary Interactions of BmiRNAs with mRNAs of Human Genes
Source: Int J Mol Sci. 2026 Sep 30;27(19):8773. doi: 10.3390/ijms27198773 (PMC13665148; doi:10.3390/ijms27198773)
Supplement: Supplementary file 1 [file ijms-27-08773-s001.zip › Table S6.pdf]

Table S6. Quantitative Characteristics of BmiRNA Binding to mRNAs of Genes Beginning with "F".

Gene;BmiRNA; BS,nt; ΔG,kJ/mol; BmiRNA length,nt	
F11R;BmiRNA-1096-3p;3468;-108;20	*FBXO27*;BmiRNA-1641-5p;1765;-102;18
F11R;BmiRNA-1189-3p;3297;-113;20	*FBXO27*;BmiRNA-1641-5p;2061;-102;18
F11R;BmiRNA-1441-3p;3295;-123;22	*FBXO40*;BmiRNA-1791-5p;3701;-102;19
F11R;BmiRNA-2164-3p;3296;-113;20	*FBXO44*;BmiRNA-1169-3p;2989;-115;20
F2RL3;BmiRNA-531-5p;2094;-108;19	*FBXO44*;BmiRNA-2289-3p;2991;-115;20
F2RL3;BmiRNA-531-5p;2429;-108;19	*FBXO6*;BmiRNA-531-5p;1460;-108;19
F2RL3;BmiRNA-1608-5p;2096;-108;19	*FBXO6*;BmiRNA-1641-5p;1461;-102;18
F2RL3;BmiRNA-1641-5p;2095;-102;18	*FBXW2*;BmiRNA-2289-3p;5558;-115;20
F2RL3;BmiRNA-1641-5p;2430;-102;18	*FCAR*;BmiRNA-1189-3p;1198;-113;20
F2RL3;BmiRNA-2087-3p;2377;-102;19	*FCAR*;BmiRNA-1441-3p;1196;-123;22
FADS1;BmiRNA-531-5p;3793;-108;19	*FCAR*;BmiRNA-2164-3p;1197;-113;20
FADS1;BmiRNA-1641-5p;3794;-102;18	*FCF1*;BmiRNA-1791-5p;1714;-102;19
FAIM;BmiRNA-1441-3p;255**;-123;22	*FCF1*;BmiRNA-2289-3p;1328;-115;20
FAIM;BmiRNA-2164-3p;256**;-113;20	*FCF1*;BmiRNA-2289-3p;1661;-115;20
FAIM;BmiRNA-1189-3p;257**;-113;20	*FCGR2A*;BmiRNA-1189-3p;1509;-113;20
FAIM;BmiRNA-2289-3p;259**;-115;20	*FECH*;BmiRNA-531-5p;4428;-108;19
FAM105A;BmiRNA-1791-5p;3887;-102;19	*FECH*;BmiRNA-1641-5p;4429;-102;18
FAM105B;BmiRNA-1169-3p;5380;-115;20	*FGB*;BmiRNA-531-5p;2293;-108;19
FAM115A;BmiRNA-531-5p;3957;-108;19	*FGB*;BmiRNA-1608-5p;2295;-108;19
FAM115A;BmiRNA-1641-5p;3958;-102;18	*FGB*;BmiRNA-1641-5p;2294;-102;18
FAM117B;BmiRNA-1449-3p;3214;-115;21	*FGD4*;BmiRNA-754-5p;7690;-119;22
FAM122C;BmiRNA-1169-3p;535*; -115;20	*FGD6*;BmiRNA-531-5p;6027;-108;19
FAM122C;BmiRNA-2289-3p;537*; -115;20	*FGD6*;BmiRNA-1641-5p;6028;-102;18
FAM122C;BmiRNA-1791-5p;590*; -102;19	*FGL2*;BmiRNA-2087-3p;1872;-102;19
FAM124A;BmiRNA-1449-3p;3740;-115;21	*FHL2*;BmiRNA-1169-3p;453**;-115;20
FAM126B;BmiRNA-754-5p;4360;-119;22	*FHL2*;BmiRNA-2289-3p;455**;-115;20
FAM129A;BmiRNA-1096-3p;4595;-108;20	*FHL5*;BmiRNA-531-5p;5009;-108;19
FAM134C;BmiRNA-1096-3p;2147;-108;20	*FHL5*;BmiRNA-1641-5p;5010;-102;18
FAM134C;BmiRNA-1169-3p;1971;-115;20	*FKBP14*;BmiRNA-531-5p;2356;-108;19
FAM134C;BmiRNA-1169-3p;2300;-115;20	*FKBP14*;BmiRNA-531-5p;4003;-108;19
FAM134C;BmiRNA-2289-3p;2302;-115;20	*FKBP14*;BmiRNA-754-5p;2200;-119;22
FAM177A1;BmiRNA-531-5p;2345;-108;19	*FKBP14*;BmiRNA-1641-5p;2357;-102;18
FAM177A1;BmiRNA-1641-5p;2346;-102;18	*FKBP14*;BmiRNA-1641-5p;4004;-102;18
FAM18A;BmiRNA-1169-3p;1430;-115;20	*FKBP5*;BmiRNA-1189-3p;4876;-113;20
FAM18A;BmiRNA-1791-5p;1485;-102;19	*FKBP5*;BmiRNA-1189-3p;6373;-113;20
FAM18A;BmiRNA-2289-3p;1432;-115;20	*FKBP5*;BmiRNA-1441-3p;4874;-123;22
FAM18B2;BmiRNA-1169-3p;1136;-115;20	*FKBP*5;BmiRNA-1441-3p;6371;-123;22
FAM18B2;BmiRNA-1169-3p;1626;-115;20	*FKBP5*;BmiRNA-2164-3p;4875;-113;20
FAM18B2;BmiRNA-1169-3p;2639;-115;20	*FKBP5*;BmiRNA-2164-3p;6372;-113;20
FAM18B2;BmiRNA-2289-3p;1628;-115;20	*FKBP5*;BmiRNA-2289-3p;4878;-115;20
FAM18B2;BmiRNA-2289-3p;2641;-115;20	*FKBP5*;BmiRNA-2289-3p;6375;-115;20
FAM200A;BmiRNA-754-5p;2205;-119;22	*FLCN*;BmiRNA-1096-3p;3307;-108;20
FAM20B;BmiRNA-1169-3p;2734;-115;20	*FLCN*;BmiRNA-1449-3p;3133;-115;21
FAM20B;BmiRNA-2289-3p;2736;-115;20	*FLG2*;BmiRNA-531-5p;8654;-108;19
FAM210B;BmiRNA-531-5p;1966;-108;19	*FLG2*;BmiRNA-1641-5p;8655;-102;18
FAM210B;BmiRNA-1641-5p;1967;-102;18	*FLVCR1*;BmiRNA-531-5p;4057;-108;19
FAM212B;BmiRNA-754-5p;2093;-119;22	*FLVCR1*;BmiRNA-1641-5p;3705;-102;18
FAM219B;BmiRNA-1189-3p;2291;-113;20	*FLVCR1*;BmiRNA-1641-5p;4058;-102;18
FAM219B;BmiRNA-1441-3p;2289;-123;22	*FMN1*;BmiRNA-531-5p;6764;-108;19
FAM219B;BmiRNA-2164-3p;2290;-113;20	*FMN1*;BmiRNA-1641-5p;6765;-102;18
FAM40B;BmiRNA-1791-5p;3824;-102;19	*FNBP1*;BmiRNA-1096-3p;3235;-108;20
FAM40B;BmiRNA-1927-5p;3807;-113;19	*FOXJ3*;BmiRNA-1169-3p;3128;-115;20
FAM60A;BmiRNA-1189-3p;1104;-113;20	*FOXJ3*;BmiRNA-1927-5p;3166;-113;19
FAM60A;BmiRNA-2289-3p;1106;-115;20	*FOXJ3*;BmiRNA-2289-3p;3130;-115;20

FAM73A;BmiRNA-531-5p;2962;-108;19	*FOXK1*;BmiRNA-531-5p;10238;-108;19
FAM73A;BmiRNA-531-5p;3139;-108;19	*FOXK1*;BmiRNA-1169-3p;7920;-115;20
FAM73A;BmiRNA-1169-3p;3917;-115;20	*FOXK1*;BmiRNA-1169-3p;9226;-115;20
FAM73A;BmiRNA-1608-5p;2964;-108;19	*FOXK1*;BmiRNA-1608-5p;10240;-108;19
FAM73A;BmiRNA-1641-5p;2963;-102;18	*FOXK1*;BmiRNA-1641-5p;10239;-102;18
FAM73A;BmiRNA-1641-5p;3140;-102;18	*FOXK1*;BmiRNA-2289-3p;7922;-115;20
FAM76A;BmiRNA-1791-5p;298*;-102;19	*FOXK1*;BmiRNA-2289-3p;9228;-115;20
FAM84B;BmiRNA-531-5p;3855;-108;19	*FREM2*;BmiRNA-1449-3p;15467;-115;21
FAM84B;BmiRNA-1608-5p;3857;-108;19	*FRG2*;BmiRNA-1189-3p;1291;-113;20
FAM84B;BmiRNA-1641-5p;3856;-102;18	*FRG2*;BmiRNA-2289-3p;1293;-115;20
FANCC;BmiRNA-531-5p;2957;-108;19	*FSBP*;BmiRNA-1791-5p;2881;-102;19
FANCC;BmiRNA-1608-5p;2959;-108;19	*FUS*;BmiRNA-1449-3p;3247;-115;21
FANCC;BmiRNA-1641-5p;2958;-102;18	*FUS*;BmiRNA-2289-3p;2850;-115;20
FASTKD2;BmiRNA-2451-3p;5233;-102;18	*FUT1*;BmiRNA-1169-3p;2665;-115;20
FAT3;BmiRNA-1096-3p;15201;-108;20	*FUT1*;BmiRNA-2289-3p;2667;-115;20
FAT3;BmiRNA-1791-5p;15084;-102;19	*FUT2*;BmiRNA-1096-3p;2486;-108;20
FBLIM1;BmiRNA-531-5p;2354;-108;19	*FUT2*;BmiRNA-1791-5p;2370;-102;19
FBLIM1;BmiRNA-754-5p;1897;-119;22	*FUT2*;BmiRNA-2289-3p;2317;-115;20
FBLIM1;BmiRNA-1641-5p;2355;-102;18	*FUT4*;BmiRNA-1791-5p;4487;-102;19
FBXL13;BmiRNA-754-5p;2920;-119;22	*FXC1*;BmiRNA-1441-3p;2287;-123;22
FBXL18;BmiRNA-754-5p;3248;-119;22	*FXC1*;BmiRNA-2164-3p;2288;-113;20
FBXL18;BmiRNA-2289-3p;6344;-115;20	*FXN*;BmiRNA-531-5p;5988;-108;19
FBXL20;BmiRNA-531-5p;8889;-108;19	*FXN*;BmiRNA-1189-3p;5053;-113;20
FBXL20;BmiRNA-754-5p;8764;-119;22	*FXN*;BmiRNA-1441-3p;5051;-123;22
*FBXL*20;BmiRNA-1449-3p;7203;-115;21	*FXN*;BmiRNA-1641-5p;5989;-102;18
*FBXL*20;BmiRNA-1608-5p;8891;-108;19	*FXN*;BmiRNA-2164-3p;5052;-113;20
*FBXL*20;BmiRNA-1641-5p;8890;-102;18	*FXN*;BmiRNA-2289-3p;5055;-115;20
*FBXL*20;BmiRNA-2289-3p;7612;-115;20	*FZD2*;BmiRNA-1169-3p;3321;-115;20
FBXO27;BmiRNA-531-5p;1764;-108;19	*FZD2*;BmiRNA-2083-3p;3535;-106;20
FBXO27;BmiRNA-531-5p;2060;-108;19	*FZD3*;BmiRNA-1169-3p;6712;-115;20
FBXO27;BmiRNA-754-5p;1607;-119;22	*FZD3*;BmiRNA-1608-5p;10105;-108;19
FBXO27;BmiRNA-1608-5p;1766;-108;19	*FZD3*;BmiRNA-2289-3p;6714;-115;20

* - CDS; ** - 5'UTR.
