# Supplementary material for: Features of Complementary Interactions of BmiRNAs with mRNAs of Human Genes
Source: Int J Mol Sci. 2026 Sep 30;27(19):8773. doi: 10.3390/ijms27198773 (PMC13665148; doi:10.3390/ijms27198773)
Supplement: Supplementary file 1 [file ijms-27-08773-s001.zip › Table S7 .pdf]

**Table S7**. Quantitative Characteristics of BmiRNA Binding to mRNAs of Genes Beginning with "G".

| Gene; BmiRNA; BS, nt; ΔG, kJ/mol; BmiRNA length, nt | |
|---|---|
| *G6PC*; BmiRNA-1379-5p; 2389 ;-102; 19 | *GMEB1*; BmiRNA-2087-3p; 2600; -102; 19 |
| *G6PC2*; BmiRNA-1169-3p; 1814 ;-115; 20 | *GNAI3*; BmiRNA-1169-3p; 3592; -115; 20 |
| *GAGE1*; BmiRNA-1379-5p ;461*; -102; 19 | *GNAI3*; BmiRNA-2289-3p;3594;-115;20 |
| *GAL3ST4*; BmiRNA-1791-5p;2139;-102;19 | *GNAL*; BmiRNA-1169-3p;4527;-115;20 |
| *GAL3ST4*; BmiRNA-2289-3p;2086;-115;20 | *GNAL*; BmiRNA-1927-5p;4565;-113;19 |
| *GALM*; BmiRNA-2087-3p;2014;-102;19 | *GNAL*; BmiRNA-2289-3p;4529;-115;20 |
| *GALNT4*; BmiRNA-531-5p;3645;-108;19 | *GNE*; BmiRNA-531-5p;3025;-108;19 |
| *GALNT4*; BmiRNA-1641-5p;3646;-102;18 | *GNE*; BmiRNA-1189-3p;4462;-113;20 |
| *GALNT6*; BmiRNA-531-5p;2761;-108;19 | *GNE*; BmiRNA-1441-3p;4460;-123;22 |
| *GALNT6*; BmiRNA-1608-5p;2763;-108;19 | *GNE*; BmiRNA-1641-5p;3026;-102;18 |
| *GALNT6*; BmiRNA-1641-5p;2762;-102;18 | *GNE*; BmiRNA-2289-3p;4464;-115;20 |
| *GAMT*; BmiRNA-531-5p;1086;-108;19 | *GNG4*; BmiRNA-1791-5p;1325;-102;19 |
| *GAMT*; BmiRNA-1641-5p;1087;-102;18 | *GNG7*; BmiRNA-1791-5p;1346;-102;19 |
| *GAPVD1*; BmiRNA-1449-3p;6161;-115;21 | *GNL3L*; BmiRNA-531-5p;4155;-108;19 |
| *GATAD1*; BmiRNA-531-5p;1953;-108;19 | *GNL3L*; BmiRNA-1641-5p;4156;-102;18 |
| *GATAD1*; BmiRNA-531-5p;2626;-108;19 | *GNL3L*; BmiRNA-1791-5p;6231;-102;19 |
| *GATAD1*; BmiRNA-1641-5p;1954;-102;18 | *GNPTAB*; BmiRNA-1169-3p;5117;-115;20 |
| *GATAD1*; BmiRNA-1641-5p;2627;-102;18 | *GNPTAB*; BmiRNA-1927-5p;5155;-113;19 |
| *GATC*; BmiRNA-531-5p;1459;-108;19 | *GNPTAB*; BmiRNA-2289-3p;5119;-115;20 |
| *GATC*; BmiRNA-1189-3p;1635;-113;20 | *GNS*; BmiRNA-1791-5p;3129;-102;19 |
| *GATC*; BmiRNA-1441-3p;1633;-123;22 | *GOLGA2*; BmiRNA-2087-3p;3468;-102;19 |
| *GATC*; BmiRNA-1641-5p;1460;-102;18 | *GOLGA3;* BmiRNA-1169-3p;5639;-115;20 |
| *GATC*; BmiRNA-2164-3p;1634;-113;20 | *GOLGA3*; BmiRNA-1169-3p;7950;-115;20 |
| *GBP4*; BmiRNA-531-5p;4753;-108;19 | *GOLGA3*; BmiRNA-1791-5p;8005;-102;19 |
| *GBP4*; BmiRNA-1641-5p;4754;-102;18 | *GOLGA3*; BmiRNA-2289-3p;5641;-115;20 |
| *GBP4*; BmiRNA-2164-3p;3434;-113;20 | *GOLGA3*; BmiRNA-2289-3p;7952;-115;20 |
| *GCLM*; BmiRNA-531-5p;2579;-108;19 | *GOLGA6L1*; BmiRNA-2087-3p;2412;-102;19 |
| *GCLM*; BmiRNA-1641-5p;2580;-102;18 | *GOLGA6L6*; BmiRNA-2087-3p;2682;-102;19 |
| *GCNT2*; BmiRNA-531-5p;4285;-108;19 | *GOSR1*; BmiRNA-1169-3p;1460;-115;20 |
| *GCNT2*; BmiRNA-1641-5p;4286;-102;18 | *GOSR1*; BmiRNA-2289-3p;1462;-115;20 |
| *GCOM1*; BmiRNA-1169-3p;2477;-115;20 | *GPI*; BmiRNA-1169-3p;3693;-115;20 |
| *GCOM1*; BmiRNA-2289-3p;2479;-115;20 | *GPI*; BmiRNA-1927-5p;2646;-113;19 |
| *GDPD1*; BmiRNA-531-5p;1787;-108;19 | *GPLD1*; BmiRNA-1169-3p;3842;-115;20 |
| *GDPD1*; BmiRNA-531-5p;2052;-108;19 | *GPR12*; BmiRNA-531-5p;3804;-108;19 |
| *GDPD1*; BmiRNA-1641-5p;1788;-102;18 | *GPR12*; BmiRNA-1641-5p;3805;-102;18 |
| *GDPD1*; BmiRNA-2385-3p;1420;-104;21 | *GPR155*; BmiRNA-531-5p;6303;-108;19 |
| *GDPD1*; BmiRNA-2385-5p;1458;-102;21 | *GPR155*; BmiRNA-1641-5p;6304;-102;18 |
| *GFPT1*; BmiRNA-1367-5p;4737;-123;22 | *GPR37L1*; BmiRNA-1791-5p;2056;-102;19 |
| *GGA2*; BmiRNA-1169-3p;5570;-115;20 | *GPR4*; BmiRNA-1521-5p;2479;-108;20 |
| *GGA2*; BmiRNA-2289-3p;5572;-115;20 | *GPR63*; BmiRNA-1189-3p;3001;-113;20 |
| *GGCX*; BmiRNA-1189-3p;6883;-113;20 | *GPR63*; BmiRNA-1927-5p;263**;-113;19 |
| *GGCX*; BmiRNA-1441-3p;6881;-123;22 | *GPR65*; BmiRNA-1791-5p;2852;-102;19 |
| *GGCX*; BmiRNA-2289-3p;6885;-115;20 | *GRB2*; BmiRNA-2087-3p;1823;-102;19 |
| *GINS3*; BmiRNA-1169-3p;409*;-115;20 | *GREB1*; BmiRNA-1189-3p;7221;-113;20 |
| *GINS4*; BmiRNA-2087-3p;1086;-102;19 | *GREB1*; BmiRNA-1441-3p;7219;-123;22 |
| *GJC1*; BmiRNA-531-5p;6828;-108;19 | *GRINL1A*; BmiRNA-1169-3p;1940;-115;20 |
| *GJC1*; BmiRNA-1641-5p;6829;-102;18 | *GRINL1A*; BmiRNA-2289-3p;1942;-115;20 |
| *GJC1*; BmiRNA-1791-5p;4885;-102;19 | *GRIPAP1*; BmiRNA-531-5p;2781;-108;19 |
| *GK5*; BmiRNA-531-5p;6587;-108;19 | *GRIPAP1*; BmiRNA-1608-5p;2783;-108;19 |
| *GK5*; BmiRNA-1169-3p;4390;-115;20 | *GRIPAP1*; BmiRNA-1641-5p;2782;-102;18 |
| *GK5*; BmiRNA-1608-5p;6589;-108;19 | *GRM6*; BmiRNA-1379-5p;5392;-102;19 |
| *GK5*; BmiRNA-1641-5p;6588;-102;18 | *GRM6*; BmiRNA-2087-3p;3726;-102;19 |
| *GK5*; BmiRNA-2289-3p;4392;-115;20 | *GRSF1*; BmiRNA-2087-3p;4264;-102;19 |
| *GLB1L3*; BmiRNA-1169-3p;2374;-115;20 | *GSDMA*; BmiRNA-1189-3p;1729;-113;20 |

| | |
|---|---|
| *GLB1L3*; BmiRNA-2289-3p;2376;-115;20 | *GSDMA*; BmiRNA-1441-3p;1727;-123;22 |
| *GLG1*; BmiRNA-1169-3p;8027;-115;20 | *GSDMA*; BmiRNA-2289-3p;1731;-115;20 |
| *GLG1*; BmiRNA-2289-3p;8029;-115;20 | *GTF2E1*; BmiRNA-1791-5p;1774;-102;19 |
| *GLRX3*; BmiRNA-1189-3p;1101;-113;20 | *GTF2F1*; BmiRNA-531-5p;2487;-108;19 |
| *GLRX3*; BmiRNA-1441-3p;1099;-123;22 | *GTF2F1*; BmiRNA-1641-5p;2488;-102;18 |
| *GLRX3*; BmiRNA-2289-3p;1103;-115;20 | *GTF2H5*; BmiRNA-531-5p;2090;-108;19 |
| *GLT25D1*; BmiRNA-1189-3p;2388;-113;20 | *GTF2H5*; BmiRNA-1641-5p;2091;-102;18 |
| *GLT25D1*; BmiRNA-1441-3p;2386;-123;22 | *GTPBP3*; BmiRNA-1169-3p;2110;-115;20 |
| *GMEB1*; BmiRNA-531-5p;2653;-108;19 | *GTSE1*; BmiRNA-531-5p;2744;-108;19 |
| *GMEB1*; BmiRNA-1608-5p;2655;-108;19 | *GTSE1*; BmiRNA-1608-5p;2746;-108;19 |
| *GMEB1*; BmiRNA-1641-5p;2654; -102; 18 | *GTSE1*; BmiRNA-1641-5p;2745;-102;18 |

* - CDS; ** - 5'UTR.
