# Supplementary material for: Features of Complementary Interactions of BmiRNAs with mRNAs of Human Genes
Source: Int J Mol Sci. 2026 Sep 30;27(19):8773. doi: 10.3390/ijms27198773 (PMC13665148; doi:10.3390/ijms27198773)
Supplement: Supplementary file 1 [file ijms-27-08773-s001.zip › Table S8.pdf]

Table S8. Quantitative Characteristics of BmiRNA Binding to mRNAs of Genes Beginning with "H".

**Gene; BmiRNA; BS, nt; ΔG, kJ/mole; BmiRNA length, nt**	
H6PD;BmiR-1169-3p; 8761; -115; 20	*HOGA1*;BmiR-1791-5p; 1778; -102; 19
H6PD;BmiR-2289-3p; 8763; -115; 20	*HOOK3*;BmiR-531-5p; 7066; -108; 19
HAUS3;BmiR-1169-3p; 4168; -115; 20	*HOOK3*;BmiR-1169-3p; 10832; -115; 20
HAUS3;BmiR-2289-3p; 4170; -115; 20	*HOOK3*;BmiR-1189-3p; 5609; -113; 20
HAVCR2; BmiR-1791-5p; 1691; -102; 19	*HOOK3*;BmiR-1441-3p; 5607; -123; 22
HCAR1;BmiR-2289-3p; 1640;; -115; 20	*HOOK3*;BmiR-1641-5p; 7067; -102; 18
HEATR3;BmiR-1641-5p; 2566;; -102;18	*HOOK3*;BmiR-1641-5p; 12217; -102; 18
HEATR5A; BmiR-531-5p; 6990; -108; 19	*HOOK3*;BmiR-1791-5p; 7448; -102; 19
HEATR5A; BmiR-1641-5p; 6991; -102; 18	*HOOK3*;BmiR-2289-3p; 5611; -115; 20
HELZ2;BmiR-531-5p; 9491; -108; 19	*HOOK3*;BmiR-2289-3p; 10834; -115; 20
HELZ2;BmiR-1641-5p; 9492; -102; 18	*HPSE*;BmiR-531-5p; 2986; -108; 19
HES2;BmiR-531-5p; 3657; -108; 19	*HPSE*;BmiR-1641-5p; 2987; -102; 18
HES2;BmiR-1641-5p; 3658; -102; 18	*HRH1*;BmiR-531-5p; 3101; -108; 19
HIF3A; BmiR-1189-3p; 2991; -113; 20	*HRH1*;BmiR-1608-5p; 3103; -108; 19
HIF3A; BmiR-1441-3p; 2989; -123; 22	*HRH1*;BmiR-1641-5p; 3102; -102; 18
HIF3A; BmiR-2289-3p; 2993; -115; 20	*HSD11B1L*; BmiR-1791-5p; 556*; -102; 19
HINT3;BmiR-531-5p; 2849; -108; 19	*HSD17B13*; BmiR-1169-3p; 1897; -115; 20
HINT3;BmiR-1641-5p; 2850; -102; 18	*HSD17B7*;BmiR-531-5p; 1338; -108; 19
HIP1;BmiR-531-5p; 6835; -108; 19	*HSD17B7*;BmiR-1641-5p; 1339; -102; 18
HIP1;BmiR-1641-5p; 6836; -102; 18	*HUNK*;BmiR-1189-3p; 5447; -113; 20
HIST1; BmiR-2289-3p; 1932; -115; 20	*HUNK*;BmiR-1441-3p; 5445; -123; 22
HLA-E;BmiR-1641-5p; 1711; -102; 18	*HUNK*;BmiR-2289-3p; 5449; -115; 20
HMBS;BmiR-2365-5p; 1291; -127; 22	
* - CDS, ** - 5UTR	
