# Supplementary material for: Features of Complementary Interactions of BmiRNAs with mRNAs of Human Genes
Source: Int J Mol Sci. 2026 Sep 30;27(19):8773. doi: 10.3390/ijms27198773 (PMC13665148; doi:10.3390/ijms27198773)
Supplement: Supplementary file 1 [file ijms-27-08773-s001.zip › Table S9.pdf]

Table S9. Quantitative Characteristics of BmiRNA Binding to mRNAs of Genes Beginning with "I".

Gene; BmiRNA; BS, nt; ΔG, kJ/mole; BmiRNA length, nt	
IAPP;BmiR-1641-5p; 1031; -102; 18	*IPO9*;BmiR-2087-3p; 4953; -102; 19
IBA57;BmiR-531-5p(; 4187; -108; 19	*IQCC*;BmiR-531-5p; 2169; -108; 19
IBA57;BmiR-1074-5p; 4515; -123; 22	*IQCC*;BmiR-1641-5p; 2170; -102; 18
IBA57;BmiR-1608-5p; 4189; -108; 19	*IRAK1*; BmiR-1641-5p; 2935; -102; 18
IBA57;BmiR-1641-5p; 4188; -102; 18	*IRAK2*;BmiR-1189-3p; 2800; -113; 20
ICAM1;BmiR-1189-3p; 3031; -113; 20	*IRAK2*;BmiR-1441-3p; 2798; -123; 22
ICAM1;BmiR-1441-3p; 3029; -123; 22	*IRAK2*;BmiR-2289-3p; 2802; -115; 20
ICAM4;BmiR-531-5p; 1312; -108; 19	*IRAK3*;BmiR-2289-3p; 2927; -115; 20
ICAM4;BmiR-1641-5p; 1313; -102; 18	*IRAK4*;BmiR-531-5p; 2814; -108; 19
ICK;BmiR-1189-3p; 3838; -113; 20	*IRAK4*;BmiR-1169-3p; 3377; -115; 20
ICK;BmiR-1441-3p; 3836; -123; 22	*IRAK4*;BmiR-1641-5p; 2815; -102; 18
ICK;BmiR-2289-3p; 3840; -115; 20	*IRAK4*;BmiR-2289-3p; 3379; -115; 20
ICMT;BmiR-1791-5p; 3662; -102; 19	*IREB2*;BmiR-1927-5p; 4841; -113; 19
IDS;BmiR-531-5p; 5539; -108; 19	*IRF1*;BmiR-531-5p; 3052; -108; 19
IDS;BmiR-1641-5p; 5540; -102; 18	*IRF1*;BmiR-1641-5p; 3053; -102; 18
IFIT3;BmiR-1608-5p; 2093; -108; 19	*IRF6*;BmiR-1169-3p; 2933; -115; 20
IFLTD1; BmiR-1169-3p; 1030; -115; 20	*IRF6*;BmiR-2289-3p; 2935; -115; 20
IFNAR1;BmiR-1169-3p; 3837; -115; 20	*IRGQ*;BmiR-531-5p; 9149; -108; 19
IFNAR1;BmiR-2289-3p; 3839; -115; 20	*IRGQ*;BmiR-1169-3p; 6170; -115; 20
IGF1;BmiR-2289-3p; 6010; -115; 20	*IRGQ*;BmiR-1189-3p; 5357; -113; 20
IKZF3;BmiR-1169-3p; 2939; -115; 20	*IRGQ*;BmiR-1189-3p; 6746; -113; 20
IKZF3;BmiR-1169-3p; 4517; -115; 20	*IRGQ*;BmiR-1441-3p; 5355; -123; 22
IKZF3;BmiR-1379-5p; 3092; -102; 19	*IRGQ*;BmiR-1441-3p; 6744; -123; 22
IKZF3;BmiR-1927-5p; 4555; -113; 19	*IRGQ*;BmiR-1608-5p; 8759; -108; 19
IKZF3;BmiR-2289-3p; 4519; -115; 20	*IRGQ*;BmiR-1641-5p; 9150; -102; 18
IL10RB;BmiR-531-5p; 1640; -108; 19	*IRGQ*;BmiR-1791-5p; 2283; -102; 19
IL10RB;BmiR-1641-5p; 1641; -102; 18	*IRGQ*;BmiR-1927-5p; 2266; -113; 19
IL17RD;BmiR-1169-3p; 6135; -115; 20	*IRGQ*;BmiR-2289-3p; 5359; -115; 20
IL17RD;BmiR-2289-3p; 6137; -115; 20	*IRGQ*;BmiR-2289-3p; 6172; -115; 20
IL23R;BmiR-531-5p; 2770; -108; 19	*IRGQ*;BmiR-2289-3p; 6748; -115; 20
IL23R;BmiR-1608-5p; 2772; -108; 19	*ISG20L2*;BmiR-531-5p; 2009; -108; 19
IL23R;BmiR-1641-5p; 2771; -102; 18	*ISG20L2*;BmiR-1641-5p; 2010; -102; 18
IL2RA;BmiR-531-5p; 2309; -108; 19	*ISLR2*;BmiR-1791-5p; 3611; -102; 19
IL2RA;BmiR-1608-5p; 2311; -108; 19	*ISLR2*;BmiR-2289-3p; 3558; -115; 20
IL2RA;BmiR-1641-5p; 2310; -102; 18	*ISPD*; BmiR-531-5p; 4425; -108; 19
IL31RA;BmiR-1791-5p; 325**-102; 19	*ISPD*; BmiR-1608-5p; 4427; -108; 19
IL6R;BmiR-531-5p; 3271; -108; 19	*ISPD*; BmiR-1641-5p; 4426; -102; 18
IL6R;BmiR-1641-5p; 3272; -102; 18	*ITGA2*;BmiR-531-5p; 6154; -108; 19
IMPAD1; BmiR-1791-5p; 3396; -102; 19	*ITGA2*;BmiR-1641-5p; 6155; -102; 18
INIP;BmiR-531-5p; 1475; -108; 19	*ITGAX*;BmiR-1927-5p; 3975; -113; 19
INIP;BmiR-1641-5p; 1476; -102; 18	*ITPRIPL2*;BmiR-531-5p; 4493; -108; 19
INIP;BmiR-2087-3p; 1423; -102; 19	*ITPRIPL2*;BmiR-1169-3p; 6190; -115; 20
INMT;BmiR-1169-3p; 1404; -115; 20	*ITPRIPL2*;BmiR-1641-5p; 4494; -102; 18
INMT;BmiR-2289-3p; 1406; -115; 20	*ITPRIPL2*;BmiR-2289-3p; 6192; -115; 20
INTS2;BmiR-1169-3p; 4913; -115; 20	*IYD*;BmiR-531-5p; 1887; -108; 19
IPCEF1; BmiR-1169-3p; 5582; -115; 20	*IYD*;BmiR-1608-5p; 1889; -108; 19
IPCEF1; BmiR-2289-3p; 5584; -115; 20	*IYD*;BmiR-1641-5p; 1888; -102; 18

* - CDS, ** - 5'UTR.
